# Structural Congeners of Izenamides Responsible for Cathepsin D Inhibition: Insights from Synthesis-Derived Elucidation †

**DOI:** 10.3390/md21050281

**Published:** 2023-04-28

**Authors:** Hyun Su Kim, Hyejin Kong, Taewoo Kim, Changjin Lim, Seungbeom Lee, Seok-Ho Kim, Young-Ger Suh

**Affiliations:** 1 College of Pharmacy and Institute of Pharmaceutical Sciences, CHA University, 120 Haeryong-ro, Pocheon 11160, Republic of Korea; khs8812@snu.ac.kr (H.S.K.); konghj9443@naver.com (H.K.); taewookim@snu.ac.kr (T.K.); lastchaos21c@snu.ac.kr (S.L.); 2 School of Pharmacy, Jeonbuk National University, Jeonju 54896, Republic of Korea; limcj@jbnu.ac.kr; 3 College of Pharmacy, Kangwon National University, Chuncheon 24341, Republic of Korea; ksh3410@kangwon.ac.kr

**Keywords:** marine cyanobacteria, izenamides, cathepsin D, linear depsipeptides, statine

## Abstract

This study aimed to elucidate the structural congeners of natural izenamides A, B, and C (**1**–**3**) responsible for cathepsin D (CTSD) inhibition. Structurally modified izenamides were synthesized and biologically evaluated, and their biologically important core structures were identified. We confirmed that the natural statine (Sta) unit (3*S*,4*S*)-γ-amino-β-hydroxy acid is a requisite core structure of izenamides for inhibition of CTSD, which is closely related to the pathophysiological roles in numerous human diseases. Interestingly, the statine-incorporated izenamide C variant (**7**) and 18-*epi*-izenamide B variant (**8**) exhibited more potent CTSD-inhibitory activities than natural izenamides.

## 1. Introduction

Marine cyanobacteria are promising natural sources for drug discovery, as they produce diverse secondary metabolites with various biological relevance. Especially, they can produce numerous modified peptides or depsipeptides with various selectivity profiles and therapeutic potential for protease inhibition [1].

Cathepsin D (CTSD) is a lysosomal aspartic protease that plays critical roles in various physiological and pathological processes [2,3,4,5]. It is involved in metabolic proteolysis, energy metabolism, polypeptide hormone and antigen processing, fibrinolysis, activation of enzyme precursors, apoptotic cell death, and maintenance of intracellular homeostasis [2,6,7,8,9,10,11]. CTSD is widely expressed in various cells of the human body [2,12], with particularly high abundance in the brain. CTSD dysfunction results in the impaired degradation of disease-linked proteins, and is strongly implicated in the pathogenesis of numerous human diseases, especially neurodegenerative disease, such as Alzheimer’s disease, Parkinson’s disease, Huntington’s disease, neuronal ceroid lipofuscinosis, and progressive disorders of the central nervous system [2,4,13,14]. CTSD is also associated with tumor progression, invasion, and metastasis in human malignancies, especially breast cancers, and is therefore a useful biomarker of breast cancer with poor prognosis [2,14,15,16]. Furthermore, CTSD inhibition can potentially hinder influenza virus replication by modulating host-cell autophagic/apoptotic responses [17]. Given the critical role of CTSD in human diseases, it is considered an attractive molecular target for the treatment of a wide range of diseases. However, novel therapeutic modulators of CTSD have not been sufficiently investigated, highlighting the urgent need for such modulators.

**Figure 1:**
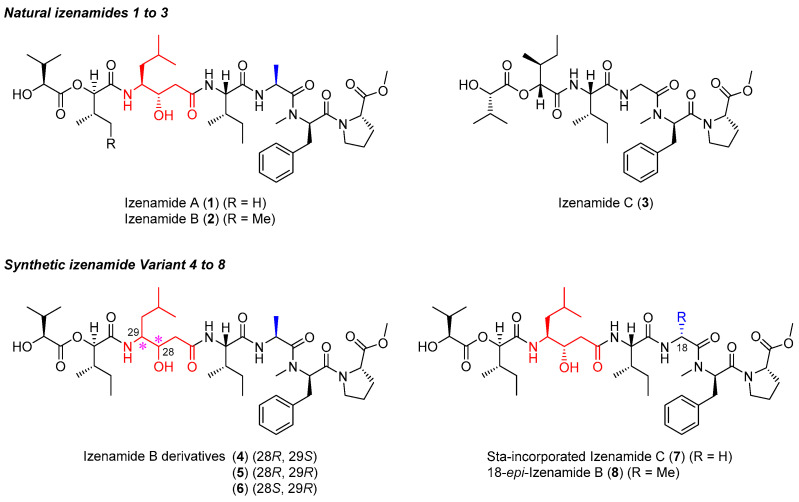
Structures of natural izenamides (**1**–**3**) and synthetic izenamide variants (**4**–**8**).

In 2018, izenamides A-C (**1**–**3**), which are linear depsipeptides, were first isolated from the marine cyanobacterium 1605-5 by Suenaga et al [18] (Figure 1). Izenamides A (**1**) and B (**2**) contain four amino acids (Pro-*O*-Me, *N*-Me-Phe, Ala, and Ile); two *α*-hydroxy acids (two valic acids for **1**, and one valic acid and one isoleucic acid for **2**); and a γ-amino-β-hydroxy acid moiety known as statine [(3*S*,4*S*)-4-amino-3-hydroxy-6-methylheptanoic acid, Sta]. Izenamide C (**3**) also contains Pro-*O*-Me, *N*-Me-Phe, Ile, and valic acid; however, it contains Gly in place of Ala in **1** and **2**, as well as isoleucic acid. Unlike izenamides A (**1**) and B (**2**), izenamide C (**3**) lacks a Sta unit in the peptide backbone. Interestingly, izenamides A (**1**) and B (**2**) show moderate inhibitory activities against CTSD in vitro, whereas izenamide C (**3**) does not show CTSD inhibition. From a structural standpoint, modified peptidic CTSD inhibitors, including pepstatin A [19,20], tasiamides [21,22,23], grassystatins [24,25], and phormidepistatin [26], also possess Sta and Sta-like moieties for the inhibition of aspartic proteases, suggesting the role of the Sta unit (in **1** and **2**) in CTSD inhibition.

In a previous study, we reported the first total syntheses of izenamides A–C (**1**–**3**) and the structural confirmation of **2**. Since then, we have been interested in elucidating the pharmacophore of izenamides through systematic structure investigation. We have been particularly interested in the Sta unit, because izenamides C (**3**) lacks CTSD inhibition [27] without Sta. We anticipated that the core component responsible for the biological activity of izenamides could be identified through structural variation, followed by biological evaluation. In addition, we desired to develop an optimal izenamide variant structure for enhanced CTSD inhibition. The strategy involved initial studies of the Sta unit, including its stereochemical effects on CTSD inhibition by the most potent izenamide, B (**2**). The study also involved investigating the Ala units of **1** and **2** and their stereochemical effects on CTSD inhibition.

This approach involved the initial preparation of izenamide B variants (**4**, **5**, and **6**) consisting of an unnatural diastereomeric Sta unit. A natural Sta-incorporated izenamide C variant (**7**) was also prepared to confirm the Sta effects of **1** and **2** on CTSD inhibition. To investigate the stereochemical effect of alanine on izenamide B (**2**), 18-*epi*-izenamide B (**8**) consisting of an unnatural d-Ala was prepared (Figure 1). Here, the design and synthesis of izenamide variants and their biological evaluation as CTSD inhibitors are described. In particular, the core structure of izenamides for CTSD inhibition and the optimized structure for improved CTSD-inhibitory activity were identified. In addition, the efficient synthesis of izenamide variants with minimized epimerization of the stereocenters was explored by employing a versatile and diverse synthetic route to prevent 2,5-diketopiperazine (DKP) formation.

## 2. Results and Discussion

### 2.1. Synthetic Strategy for Izenamide Variants (**4**–**8**)

**Scheme 1:**
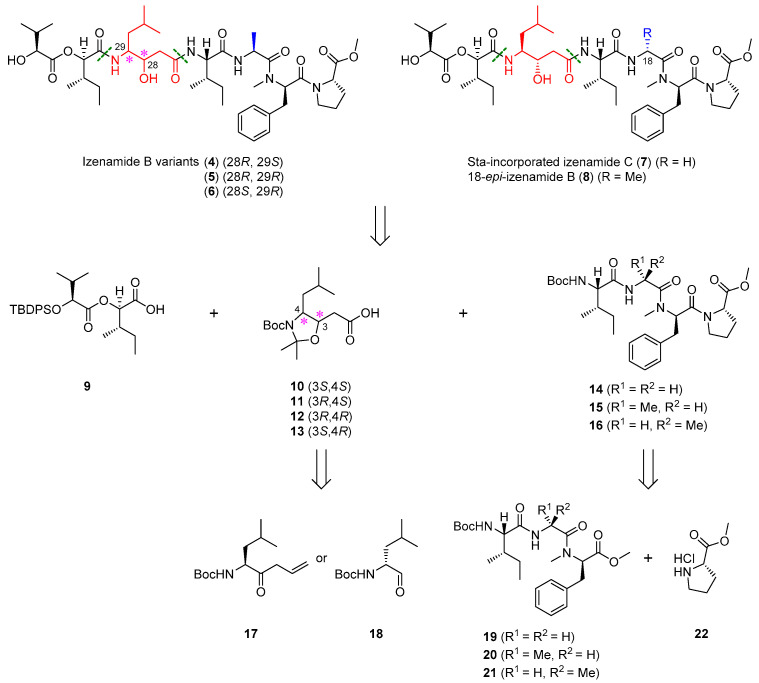
Synthetic approach for the preparation of izenamide variants (**4**–**8**).

The synthetic strategy for the novel izenamide variants (**4**–**6**) is outlined in Scheme 1, focusing on the diastereomeric modification of the natural Sta and alanine units in izenamide B (**2**). The envisioned approach involves the convergent assembly of three fragments: tetrapeptides **14**–**16**, diastereomeric Sta precursors **11**–**13**, and acid **9**. Tetrapeptides **14**–**16** were anticipated to be derived from tripeptide **19**–**21**. The diastereomers of Sta precursors **11**–**13** as key fragments would be conveniently prepared from the known ketone **17** or aldehyde **18**. Similarly, izenamide variants (**7**) and (**8**) could be synthesized by the amide coupling of tetrapeptide **14** or **15**, natural Sta precursor **10**, and acid **9**. Tetrapeptide **15** was prepared from tripeptide **20**. Fragments **9**, **10**, **14**, and **16** were readily prepared as previously described [27].

### 2.2. Synthesis of Diastereomeric Sta Precursors (**11**–**13**)

**Scheme 2:**
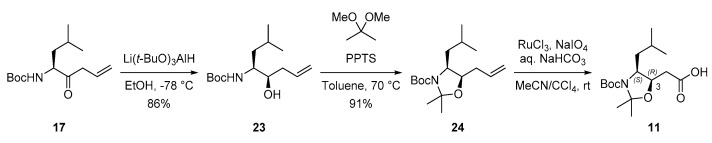
Preparation of C3-epimeric Sta precursor (3*R*,4*S*)-**11**.

The synthesis of variants **4**–**6** was initiated by preparing diastereomeric Sta precursors **11**–**13** to investigate the stereochemical effects of the two stereocenters in the Sta unit on CTSD inhibition. First, the C3-epimeric Sta precursor (3*R*,4*S*)-**11** was synthesized, as illustrated in Scheme 2. The diastereoselective reduction of ketone **17 [28]** with Li(*t*-BuO)_3_AlH exclusively afforded alcohol **23 [28]**, which was subsequently protected with 2,2-dimethoxypropane in the presence of catalytic PPTS. Finally, the desired C3-epimeric Sta precursor (3*R*,4*S*)-**11** was obtained by the RuO_4_-mediated oxidation of the terminal alkene of **24**.

**Scheme 3:**
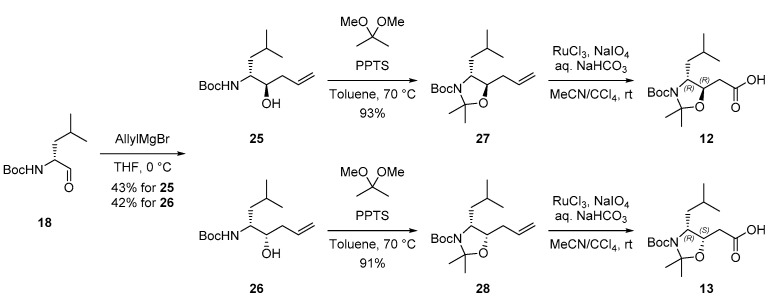
Synthesis of Sta precursors (3*R*,4*R*)-**12** and (3*S*,4*R*)-**13**.

Following the preparation of intermediate (3*R*,4*S*)-**11**, we focused on the synthesizing unnatural Sta precursors (3*R*,4*R*)-**12** and (3*S*,4*R*)-**13**, which serve as key fragments for izenamide B variants (**5**) and (**6**). Commercially available aldehyde **18** was reacted with the Grignard reagent [29], which led to the nucleophilic addition of allylMgBr to *N*-Boc-d-leucinal **18** at 0 °C. Diastereomeric mixtures of alcohols **25** and **26** were obtained in 43% and 42% yield, respectively. The structure and stereochemistry of both alcohol **25** and **26** were confirmed by comparison of their spectral data with those of the corresponding enantiomers, including the opposite optical rotation [30]. Acetonide protection of optically pure allylic alcohols **25** and **26** followed by the Ru-mediated oxidation of the terminal alkenes afforded the desired diastereomeric Sta precursors (3*R*,4*R*)-**12** and (3*S*,4*R*)-**13**, respectively (Scheme 3).

### 2.3. Completion of Izenamide Variant Synthesis (**4**–**8**)

**Scheme 4:**
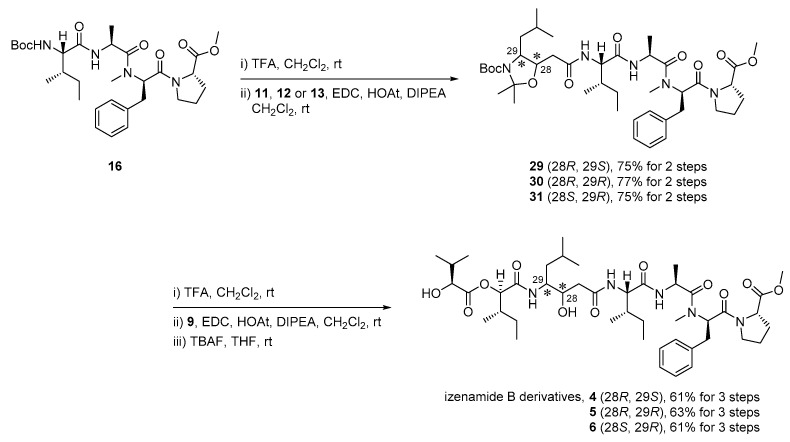
Synthesis of izenamide B variants possessing an unnatural Sta unit (**4**–**6**).

Using diastereomeric Sta precursors **11**–**13**, we assembled fragments for the izenamide B variants (**4**–**6**), as shown in Scheme 4. Boc-deprotection of tetrapeptide **16** with TFA and then amide coupling with the diastereomeric Sta precursors **11**–**13** afforded pentamers **29**–**31** in yields of 75–77% over two steps. Global deprotection of pentamers **29**–**31** with TFA and EDC-mediated coupling of the resulting amines with acid **9** produced the corresponding heptamers. Finally, the desired izenamide B variants (**4**–**6**) were obtained by desilylation in yields of 61–63% over three steps.

To investigate the role of the stereochemistry of the Ala unit of **2** in CTSD inhibition, we synthesized Sta-incorporated izenamide C (**7**) and 18-*epi*-izenamide B (**8**). The incorporation of a natural Sta unit into izenamide C (**3**), which contains a Gly unit instead of an Ala unit, was achieved at the corresponding Sta position in natural izenamide B (**2**). Additionally, the preparation of 18-*epi*-izenamide B (**8**) involved the substitution of unnatural d-Ala unit.

**Scheme 5:**
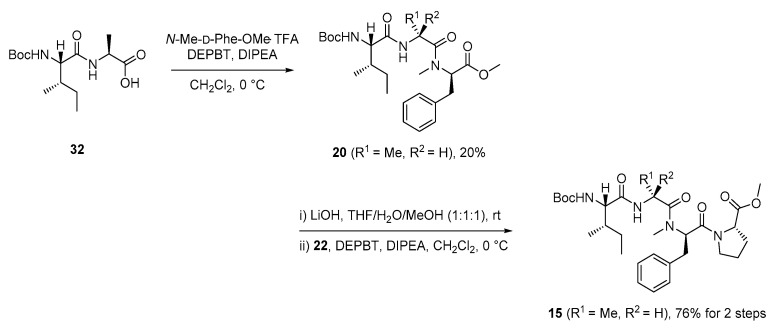
Preparation of tetrapeptide **15**.

Tripeptide **20** was synthesized by coupling the known acid **32** [27] with *N*-Me-d-Phe-OMe·TFA salt [27]. The ester hydrolysis of tripeptide **20** and subsequent amidation of the resulting acid with **22** afforded tetrapeptide **15** in a yield of 76% over two steps (Scheme 5).

**Scheme 6:**
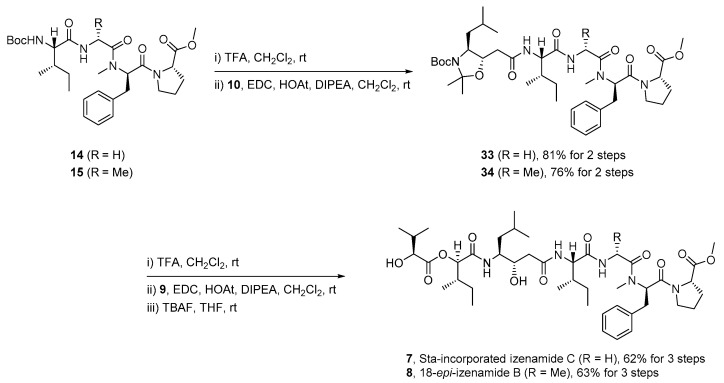
Completion of the synthesis of Sta-incorporated izenamide C (**7**) and 18-*epi*-izenamide B (**8**).

Tetrapeptide **14**, acid **9**, and natural Sta precursor **10** were prepared following a previously reported synthetic protocol [27]. The synthesis of izenamide variants **7** and **8** (Scheme 6) was completed by Boc-deprotection of tetrapeptides **14** and **15** and subsequent EDC-mediated amide coupling with acid **10**. Pentamer intermediates **33** and **34** were obtained in yields of 81% and 76%, respectively, in two steps. The global deprotection of pentamers **33** and **34** and subsequent amide coupling of the resulting amines with acid **9** produced the corresponding heptamers, which were subjected to desilylation to afford Sta-incorporated izenamide C (**7**) and 18-*epi*-izenamide B (**8**) in yields of 62% and 63% over three steps, respectively (All spectral data including ^1^H, ^13^C NMR and HRMS of izenamides (**4**–**8**) was in Supplementary Materials).

### 2.4. CTSD-Inhibitory Activities of Izenamides (**1**–**3**) and Their Variants (**4**–**8**)

After the successful synthesis of izenamide variants (**4**–**8**), the cathepsin D (CTSD)-inhibitory activities of the five izenamide variants and previously synthesized izenamides A–C (**1**–**3**) were evaluated. The CTSD-inhibition assay was performed at Eurofins Panlabs Discovery Services by measuring the percentage inhibition at concentrations of 0.5 and 1.0 μM (Table 1). Synthetic izenamides A (**1**) and B (**2**) exhibited similar inhibitory activities against CTSD, while synthetic izenamide C (**3**), which lacks the natural Sta unit, exhibited no CTSD inhibition, consistent with previous report [18].

The natural Sta units of izenamides are essential for binding to CTSD [18]. Co-crystal structural analysis and docking studies predicted that the hydroxy group of the natural Sta in pepstatin A or izenamide A interacts with Asp residues at the active site of CTSD [31]. However, the effects of the stereochemistry of the two stereocenters in the Sta unit on CTSD inhibition remains unexplored. Thus, the stereochemical effects of the Sta unit of the izenamides on their CTSD-inhibitory activity were examined. As shown in Table 1, izenamide B (**2**) exhibits moderate inhibitory effects against CTSD (8.12% at 0.5 μM and 14.76% at 1.0 μM), whereas the unnatural Sta-incorporated izenamide B variants (**4**–**6**) exhibit no CTSD-inhibitory activities. These results confirm that natural (3*S*,4*S*)-Sta is essential for CTSD by izenamides.

Notably, the izenamide C variant (**7**) exhibits more potent CTSD-inhibitory activities (26.99% at 0.5 μM and 36.07% at 1.0 μM) than those of the natural izenamide B (**2**). The Sta-incorporated izenamide C variant (**7**) possesses the same peptide sequence as the biologically active **1** and **2**, with the exception of alanine being replaced by glycine. The improved CTSD inhibition of the izenamide C variant (**7**), in contrast to the moderate inhibitory activity of the natural Sta-containing izenamide B (**2**) against CTSD, indicates that glycine is more appropriate for achieving adequate CTSD-inhibitory activity. These results confirm the crucial role played by the natural Sta unit as a key pharmacophore in the inhibition of CTSD by izenamides.

The sole difference in structure between **2** and **7** was the absence of a methyl substituent on the Gly unit at C18 in the izenamide C variant (**7**). Therefore, the optimal stereochemistry of the Ala unit in izenamide B (**2**) for CTSD inhibition was investigated. The 18-*epi*-izenamide B (**8**) was prepared, as shown in Scheme 6. Surprisingly, the 18-*epi*-izenamide B (**8**) showed the best CTSD-inhibitory effects (40.96% at 1.0 μM) compared to those of **2** and **7** (14.76% and 36.07% at 1.0 μM, respectively).

Overall, this study elucidated the crucial role of the natural Sta unit of izenamides in CTSD inhibition, with the importance of its stereochemistry. The results also suggested that the stereochemistry of the natural Sta unit is critical for its appropriate conformation to interact with Asp residues in the active binding pocket of CTSD, as described previously [18,29]. Additionally, the improved CTSD inhibition observed with the epimeric C18 unit of **8** suggested that it enhances interaction with CTSD.

## 3. Materials and Methods

### 3.1. General Information

Unless noted otherwise, all starting materials and reagents were obtained from commercial suppliers and were used without further purification. Tetrahydrofuran was distilled from sodium benzophenone ketyl. Dichloromethane, chloroform and acetonitrile were freshly distilled from calcium hydride. All solvents used for routine isolation of products and chromatography were reagent grade and glass-distilled. Reaction flasks were dried at 100 °C. Air- and moisture-sensitive reactions were performed under argon atmosphere. Flash column chromatography was performed using silica gel 60 (230–400 mesh, Merck, Darmstadt, Germany) with the indicated solvents. Thin-layer chromatography was performed using 0.25 mm silica gel plates (Merck, Darmstadt, Germany). High-resolution mass spectra (HRMS) were recorded by JMS-700 (JEOL, Tokyo, Japan) and Q-TOF 6530 MS (Agilent, Santa Clara, CA, USA). Optical rotations were measured with JASCO P-2000 digital polarimeter (JASCO, Easton, MD, USA) at ambient temperature using a 10 mm cylindrical cell. Infrared spectra were recorded on a JASCO FT-IR-4200 spectrometer (JASCO, Easton, MD, USA). ^1^H and ^13^C NMR spectra were recorded using BRUKER AVANCE-400 (Bruker, Billerica, MA, USA) and BRUKER AVANCE-800 (Bruker, Billerica, MA, USA). Chemical shifts are expressed in parts per million (ppm, *δ*) downfield from tetramethylsilane and are referenced to the deuterated solvent (CHCl_3_ or MeOH). ^1^H NMR data were reported in the order of chemical shift, multiplicity (s, singlet; d, doublet; t, triplet; q, quartet; quint, quintet; dt, doublet of triplet; dq, doublet of quartet; dqu, doublet of quintet; ddd, doublet of doublet of doublet; qd, quartet of doublet, m, multiplet and/or multiple resonances), coupling constant in hertz (Hz) and number of protons.

### 3.2. Experimental Part

*tert*-Butyl (4*S*,5*R*)-5-allyl-4-isobutyl-2,2-dimethyloxazolidine-3-carboxylate (**24**). To a solution of ketone **17** (2.20 g, 8.62 mmol) in EtOH (86 mL) was added Li(*t*-BuO)_3_AlH (3.29 g, 12.92 mmol) at −78 °C. After stirring for 1 h at the same temperature, the reaction mixture was quenched with Rochelle’s solution and extracted with Et_2_O. The combined organic layer was dried over MgSO_4_ and concentrated in vacuo. The residue was filtered through a short column of silica gel to afford alcohol **23 [28]** (2.30 g, 86%) as a colorless oil.

To a solution of the alcohol **23** (600 mg, 2.33 mmol) in toluene (8 mL) were added 2,2-dimethoxypropane (3.44 mL, 28.0 mmol) and pyridinium *p*-toluenesulfonate (58.6 mg, 0.23 mmol) at 70 °C. After stirring for 12 h at the same temperature, the reaction mixture was concentrated in vacuo. The residue was purified by flash column chromatography on silica gel (EtOAc/*n*-hexane = 1:20) to afford oxazolidine **24** (623 mg, 91%) as a colorless oil. [α${]}_{D}^{20}$= +1.74 (c 1.00, CHCl_3_); ^1^H NMR (800 MHz, CDCl_3_) *δ* 5.80–5.76 (m, 1H), 5.14 (s, 0.5H), 5.12 (s, 0.5H), 5.08 (d, *J* = 9.4 Hz, 1H), 4.03 (d, *J* = 5.2 Hz, 1H), 3.99 (d, *J* = 5.0 Hz, 0.5H), 3.82 (d, *J* = 5.1 Hz, 0.5 H), 2.35 (dt, *J* = 14.1, 6.9 Hz, 1H), 2.27 (dt, *J* = 13.2, 6.4 Hz, 1H), 1.65-1.60 (m, 1H), 1.53 (d, *J* = 28.5 Hz, 3H), 1.50 (d, *J* = 13.9 Hz, 3H), 1.45 (s, 9H), 1.43–1.36 (m, 1H), 1.33–1.21 (m, 1H), 0.94–0.89 (m, 6H); ^13^C NMR (200 MHz, CDCl_3_, a mixture of rotamers) Major rotamer *δ* 151.9, 134.3, 117.0, 92.7, 79.6, 76.6, 57.1, 39. 7, 33.4, 28.6 (3C), 27.4, 24.6, 23.8, 23.5, 22.9, minor rotamer *δ* 152.5, 134.2, 117.0, 92.2, 79.6, 76.2, 57.1, 39.4, 33.3, 28.4 (3C), 28.2, 25.2, 24.8, 23.1, 23.0; IR (thin film, neat) ν_max_ 3448, 3079, 2956, 2870, 1698, 1502, 1455, 1366, 1253, 1176, 1114, 754 cm^−1^; LR-MS (FAB+) *m/z* 298 (M + H)^+^; HR-MS (FAB+) calcd for C_17_H_32_NO_3_ (M + H)^+^ 298.2382; found 298.2374.

To a solution of oxazolidine **24** (600 mg, 1.91 mmol) in a mixture of CCl_4_, CH_3_CN, and H_2_O (1:1:2, 20 mL) were added NaHCO_3_ (1.18 g, 14.1 mmol), NaIO_4_ (2.59 g, 12.1 mmol) and RuCl_3_ (42 mg, 0.20 mmol) at room temperature. After stirring for 12 h, the reaction mixture was quenched with 1*N* HCl and extracted with EtOAc. The combined organic layer was dried over MgSO_4_ and concentrated in vacuo. The residue was filtered through a short column of silica gel to afford (3*R*,4*S*)-statine derivative **11**, which was used for the next step without further purification.

*tert*-Butyl (4*R*,5*R*)-5-allyl-4-isobutyl-2,2-dimethyloxazolidine-3-carboxylate (**27**). To a solution of aldehyde **18** (1.70 g, 7.90 mmol) in THF (27 mL) was added AllylMgBr solution (1.0 M in Et_2_O, 16.0 mL, 16.0 mmol) at 0 °C. After stirring for 12 h, the reaction mixture was quenched with 1*N* HCl and extracted with EtOAc. The combined organic layer was dried over MgSO_4_ and concentrated in vacuo. The residue was purified by flash column chromatography on silica gel (EtOAc/*n*-hexane = 1:10 to 1:5) to afford alcohol **25** (874 mg, 43%) as a colorless oil and **26** (854 mg, 42%) as white solid [28,30].

*tert*-Butyl ((4*R*,5*R*)-5-hydroxy-2-methyloct-7-en-4-yl)carbamate (**25**). [α${]}_{D}^{20}$= +23.15 (c 3.30, MeOH) (*ent*-**25**, lit. [α${]}_{D}^{23}$= −29.70 (c 3.30, MeOH) [30]); ^1^H NMR (400 MHz, CDCl_3_) *δ* 5.82 (m, 1H), 5.16-5.10 (m, 2H), 4.66 (m, 1H), 3.65-3.53 (m, 2H), 2.32-2.15 (m, 3H), 1.64 (m, 1H), 1.47 (m, 1H), 1.42 (s, 9H), 1.29 (m, 1H), 0.91 (d, *J* = 1.8 Hz, 3H), 0.90 (d, *J* = 1.8 Hz, 3H); ^13^C NMR (100 MHz, CDCl_3_) *δ* 156.2, 134.6, 118.3, 79.1, 72.7, 52.0, 41.8, 39.1, 28.4 (3C), 24.8, 23.2, 22.1; LR-MS (ESI+) *m/z* 158 (M − Boc + 2H)^+^; HR-MS (ESI+) calcd for C_9_H_20_NO (M − Boc + 2H)^+^ 158.1539; found 158.1536.

*tert*-Butyl ((4*R*,5*S*)-5-hydroxy-2-methyloct-7-en-4-yl)carbamate (**26**). [α${]}_{D}^{20}$= +12.11 (c 0.36, MeOH) (*ent*-**26**, lit. [α${]}_{D}^{23}$= −13.90 (c 0.36, MeOH) [30]); ^1^H NMR (400 MHz, CDCl_3_) *δ* 5.89-5.80 (m, 1H), 5.15-5.11 (m, 2H), 4.65-4.56 (brs, 1H), 3.65 (brs, 2H), 2.20 (m, 2H), 1.66 (m, 1H), 1.44 (s, 9H), 1.33-1.26 (m, 2H), 0.93 (d, *J* = 6.8 Hz, 3H), 0.90 (d, *J* = 6.8 Hz, 3H); ^13^C NMR (100 MHz, CDCl_3_) *δ* 156.6, 135.1, 118.2, 80.1, 74.1, 53.5, 38.8, 38.2, 28.6 (3C), 25.0, 23.9, 21.8; LR-MS (ESI+) *m/z* 158 (M − Boc + 2H)^+^; HR-MS (ESI+) calcd for C_9_H_20_NO (M − Boc + 2H)^+^ 158.1539; found 158.1534.

To a solution of alcohol **25** (530 mg, 2.06 mmol) in toluene (7 mL) were added 2,2-dimethoxypropane (3.06 mL, 25.0 mmol) and pyridinium *p*-toluenesulfonate (53 mg, 0.21 mmol) at 70 °C. After stirring for 12 h at the same temperature, the reaction mixture was concentrated in vacuo. The residue was purified by flash column chromatography on silica gel (EtOAc/*n*-hexane = 1:20) to afford oxazolidine **27** (580 mg, 93%) as a colorless oil. [α${]}_{D}^{20}$= −8.03 (*c* 1.00, CHCl_3_); ^1^H NMR (800 MHz, CDCl_3_) *δ* 5.77 (ddt, *J* = 17.2, 10.2, 7.0 Hz, 1H), 5.13 (s, 0.5H), 5.09 (s, 0,5H), 5.08 (d, *J* = 10.4 Hz, 1H), 3.86 (t, *J* = 6.4 Hz, 1H), 3.76 (s, 0.5H), 3.63 (s, 0.5H), 2.40–2.35 (m, 1H), 2.30–2.27 (m, 1H), 1.70–1.61 (m, 1H), 1.60–1.53 (m, 3H), 1.50–1.42 (m, 14H), 0.90 (d, *J* = 6.4 Hz, 6H); ^13^C NMR (200 MHz, CDCl_3_, a mixture of rotamers) Major rotamer *δ* 151.8, 134.2, 117.8, 93.8, 80.2, 60.1, 42.8, 40.4, 28.8, 28.5 (3C), 28.4, 27.8, 25.5, 24.0, 21.3, minor rotamer *δ* 156.2, 134.6, 117.8, 79.1, 72.7, 52.0, 41.9, 39.1, 28.5, 28.4 (3C), 25.5, 24.8, 23.2, 22.2, 21.3; IR (thin film, neat) *ν*_max_ 2958, 2871, 1701, 1468, 1388, 1366, 1255, 1176, 1082, 856 cm^−1^; LR-MS (FAB+) *m/z* 298 (M + H)^+^; HR-MS (FAB+) calcd for C_17_H_32_NO_3_ (M + H)^+^ 298.2382; found 298.2397.

To a solution of oxazolidine **27** (500 mg, 1.68 mmol) in a mixture of CCl_4_, CH_3_CN, and H_2_O (1:1:2, 16 mL) were added NaHCO_3_ (1.0 g, 11.9 mmol), NaIO_4_ (2.2 g, 10.3 mmol), and RuCl_3_ (35 mg, 0.17 mmol) at room temperature. After stirring for 12 h, the reaction mixture was quenched with 1*N* HCl and extracted with EtOAc. The combined organic layer was dried over MgSO_4_ and concentrated in vacuo. The residue was filtered through a short column of silica gel to afford (3*R*,4*R*)-statine derivative **12**, which was used for the next step without further purification.

*tert*-Butyl (4*R*,5*S*)-5-allyl-4-isobutyl-2,2-dimethyloxazolidine-3-carboxylate (**28**). To a solution of alcohol **26** (500 mg, 1.94 mmol) in toluene (7 mL) were added 2,2-dimethoxypropane (2.94 mL, 24.0 mmol) and pyridinium *p*-toluenesulfonate (50 mg, 0.20 mmol) at 70 °C. After stirring for 12 h at the same temperature, the reaction mixture was concentrated in vacuo. The residue was purified by flash column chromatography on silica gel (EtOAc/*n*-hexane = 1:20) to afford oxazolidine **28** (536 mg, 91%) as a colorless oil. [α${]}_{D}^{20}$= −6.12 (*c* 1.00, CHCl_3_); ^1^H NMR (800 MHz, CDCl_3_) *δ* 5.80–5.72 (m, 1H), 5.10 (s, 0.5H), 5.08 (s, 0.5H), 5.04 (d, *J* = 9.8 Hz, 1H), 3.99 (d, *J* = 5.2 Hz, 1H), 3.96 (d, *J* = 5.1 Hz, 0.5H), 3.79 (d, *J* = 5.2 Hz, 0.5H), 2.31 (dt, *J* = 14.3, 7.0 Hz, 1H), 2.25-2.21 (m, 1H), 1.60–1.56 (m, 1H), 1.50 (d, *J* = 28.0 Hz, 3H), 1.46 (d, *J* = 13.2 Hz, 3H), 1.41 (s, 9H), 1.38–1.31 (m, 1H), 1.30–1.18 (m, 1H), 0.91–0.85 (m, 6H); ^13^C NMR (200 MHz, CDCl_3_, a mixture of rotamers) Major rotamer *δ* 151.8, 134.3, 117.0, 92.6, 79.5, 76.5, 57.1, 39.6, 33.3, 28.5 (3C), 27.3, 24.6, 23.7, 23.4, 22.8, minor rotamer *δ* 152.4, 134.9, 117.7, 92.1, 79.5, 76.1, 57.1, 39.3, 33.3, 28.4 (3C), 28.2, 25.1, 24.7, 23.1, 23.0; IR (thin film, neat) *ν*_max_ 2957, 2870, 1698, 1456, 1366, 1254, 1178, 1114, 864 cm^−1^; LR-MS (FAB+) *m/z* 298 (M + H^+^); HR-MS (FAB+) calcd for C_17_H_32_NO_3_ (M + H^+^) 298.2382; found 298.2386.

To a solution of oxazolidine **28** (400 mg, 1.34 mmol) in a mixture of CCl_4_, CH_3_CN, and H_2_O (1:1:2, 16 mL) were added NaHCO_3_ (900 mg, 10.7 mmol), NaIO_4_ (1.9 g, 8.9 mmol), and RuCl_3_ (31 mg, 0.15 mmol) at room temperature. After stirring for 12 h, the reaction mixture was quenched with 1*N* HCl and extracted with EtOAc. The combined organic layer was dried over MgSO_4_ and concentrated in vacuo. The residue was filtered through a short column of silica gel to afford (3*S*,4*R*)-statine derivative **13**, which was used for the next step without further purification.

(28*R*,29*S*)-Statine derivative-l-Ile-l-Ala-NMe-d-Phe-l-Pro-OMe, Pentamer (**29**). To a solution of tetrapeptide **16** (140 mg, 0.24 mmol) in CH_2_Cl_2_ (1.6 mL) was added TFA (0.4 mL) dropwise at room temperature. After stirring for 1 h, the reaction mixture was concentrated in vacuo. The residue was used in the next step without further purification. To a solution of the above amine salt (0.24 mmol) in CH_2_Cl_2_ (2.4 mL) was added (3*R*,4S)-statine **11** (100 mg, 0.32 mmol), DIPEA (0.21 mL, 1.20 mmol), HOAt (43 mg, 0.32 mmol), and EDC·HCl (93 mg, 0.49 mmol) at room temperature. After stirring for 12 h, the reaction mixture was quenched with 1N HCl and extracted with CH_2_Cl_2_. The combined organic layer was dried over MgSO_4_ and concentrated in vacuo. The residue was purified by flash column chromatography on silica gel (EtOAc/*n*-hexane = 1:1) to give pentamer **29** (141 mg, 75% for 2 steps) as white solid. [α${]}_{D}^{20}$= +19.52 (c 1.00, CHCl_3_); ^1^H NMR (800 MHz, CDCl_3_, a mixture of rotamers) Major rotamer δ 7.22-7.18 (m, 4H), 7.16 (t, *J* = 7.2 Hz, 1H), 6.92 (d, *J* = 8.0 Hz, 1H), 6.30 (d, *J* = 6.5 Hz, 1H), 5.61 (dd, *J* = 9.5, 6.4 Hz, 1H), 4.82–4.78 (m, 1H), 4.52 (d, *J* = 8.8 Hz, 1H), 4.42 (t, *J* = 7.4 Hz, 1H), 4.23 (dd, *J* = 7.5, 5.2 Hz, 1H), 3.84 (ddd, *J* = 9.7, 5.6, 2.4 Hz, 1H), 3.71 (s, 3H), 3.64–3.61 (m, 1H), 3.39 (ddd, *J* = 10.6, 6.8, 4.2 Hz, 1H), 3.21–3.16 (m, 2H), 3.00 (s, 3H), 2.93 (dd, *J* = 14.4, 9.6 Hz, 1H), 2.37 (d, *J* = 12.9 Hz, 1H), 2.32–2.29 (m, 1H), 2.23–2.19 (m, 1H), 1.99–1.95 (m, 1H), 1.93–1.89 (m, 1H), 1.88–1.77 (m, 3H), 1.72–1.63 (m, 2H), 1.49–1.42 (m, 3H), 1.41 (s, 9H), 1.39–1.36 (m, 2H), 1.32–1.28 (m, 1H), 1.08 (ddd, *J* = 13.4, 9.6, 7.3 Hz, 1H), 0.94 (d, *J* = 6.7 Hz, 3H), 0.91 (d, *J* = 6.5 Hz, 3H), 0.88 (d, *J* = 6.9 Hz, 3H), 0.86 (t, *J* = 7.4 Hz, 3H), 0.80 (d, *J* = 6.9 Hz, 3H); ^13^C NMR (200 MHz, CDCl_3_, a mixture of rotamers) Major rotamer δ 172.7, 172.3, 172.3, 170.2, 168.0, 156.2, 136.6, 129.5 (2C), 128.2 (2C), 126.7, 94.2, 79.6, 72.4, 59.4, 58.4, 55.8, 53.2, 52.2, 47.1, 44.8, 40.7, 39.3, 36.6, 34.7, 30.5, 28.8, 28.4 (5C), 25.4, 24.8 (2C), 23.8, 21.6, 17.8, 15.8, 11.7; IR (thin film, neat) ν_max_ 3308, 2958, 1749, 1633, 1453, 1365, 1265, 1174, 749 cm^−1^; LR-MS (FAB+) m/z 732 (M − C(CH_3_)_2_ + 3H)^+^; HR-MS (FAB+) calcd for C_38_H_62_N_5_O_9_ (M − C(CH_3_)_2_ + 3H)^+^ 732.4548; found 732.4568.

(28*R*,29*R*)-Statine derivative-l-Ile-l-Ala-NMe-d-Phe-l-Pro-OMe, Pentamer (**30**). To a solution of tetrapeptide **16** (150 mg, 0.26 mmol) in CH_2_Cl_2_ (1.6 mL) was added TFA (0.4 mL) dropwise at room temperature. After stirring for 1 h, the reaction mixture was concentrated in vacuo. The residue was used in the next step without further purification. To a solution of the above amine salt (0.26 mmol) in CH_2_Cl_2_ (2.4 mL) were added (3*R*,4*R*)-statine derivative **12** (123 mg, 0.39 mmol), DIPEA (0.23 mL, 1.30 mmol), HOAt (46 mg, 0.34 mmol), and EDC·HCl (100 mg, 0.52 mmol) at room temperature. After stirring for 12 h, the reaction mixture was quenched with 1N HCl and extracted with CH_2_Cl_2_. The combined organic layer was dried over MgSO_4_ and concentrated in vacuo. The residue was purified by flash column chromatography on silica gel (EtOAc/*n*-hexane = 1:1) to give pentamer **30** (155 mg, 77% for 2 steps) as white solid. [α${]}_{D}^{20}$= +70.45 (c 0.20, CHCl_3_); ^1^H NMR (800 MHz, CDCl_3_, a mixture of rotamers) Major rotamer δ 7.21–7.17 (m, 4H), 7.16–7.14 (m, 1H), 6.91 (d, *J* = 8.1 Hz, 1H), 6.07 (d, *J* = 8.3 Hz, 1H), 5.60 (dd, *J* = 9.2, 6.6 Hz, 1H), 4.85–4.80 (m, 1H), 4.69 (d, *J* = 9.8 Hz, 1H), 4.43 (t, *J* = 7.5 Hz, 1H), 4.26 (dd, *J* = 8.3, 5.0 Hz, 1H), 3.96 (d, *J* = 10.3 Hz, 1H), 3.71 (s, 3H), 3.63–3.60 (m, 1H), 3.40–3.38 (m, 1H), 3.22-3.17 (m, 2H), 3.01 (s, 3H), 2.92 (dd, *J* = 14.3, 9.4 Hz, 1H), 2.38 (dd, *J* = 13.8, 10.5 Hz, 1H), 2.31 (dd, *J* = 13.9, 2.1 Hz, 1H), 2.21–2.20 (m, 1H), 1.99–1.96 (m, 1H), 1.92–1.90 (m, 1H), 1.86–1.80 (m, 3H), 1.68–1.64 (m, 2H), 1.53–1.49 (m, 1H), 1.45–1.44 (m, 3H), 1.43 (s, 9H), 1.40–1.39 (m, 1H), 1.33–1.32 (m, 1H), 1.10–1.07 (m, 1H), 0.93 (d, *J* = 6.7 Hz, 3H), 0.93 (d, *J* = 6.5 Hz, 3H), 0.88 (d, *J* = 6.7 Hz, 3H), 0.87 (t, *J* = 7.3 Hz, 3H), 0.81 (d, *J* = 6.9 Hz, 3H); ^13^C NMR (200 MHz, CDCl_3_, a mixture of rotamers) Major rotamer δ 172.8, 172.5, 172.3, 170.1, 167.9, 156.0, 136.6, 129.5 (2C), 128.2 (2C), 126.7, 95.1, 79.2, 70.9, 59.4, 58.2, 55.8, 52.4, 52.2, 47.0, 44.8, 41.9, 41.5, 36.7, 34.7, 30.5, 29.7, 28.8, 28.4 (3C), 28.4, 25.4, 24.8, 24.7, 23.1, 22.3, 17.9, 15.7, 11.7; IR (thin film, neat) ν_max_ 3306, 2927, 1749, 1635, 1454, 1365, 1266, 1174, 700 cm^−1^; LR-MS (FAB+) m/z 732 (M − C(CH_3_)_2_ + 3H)^+^; HR-MS (FAB+) calcd for C_38_H_62_N_5_O_9_ (M − C(CH_3_)_2_ + 3H)^+^ 732.4548; found 732.4546.

(28*S*,29*R*)-Statine derivative-l-Ile-l-Ala-NMe-d-Phe-l-Pro-OMe, Pentamer (**31**). To a solution of tetrapeptide **16** (142 mg, 0.25 mmol) in CH_2_Cl_2_ (1.6 mL) was added TFA (0.4 mL) dropwise at room temperature. After stirring for 1 h, the reaction mixture was concentrated in vacuo. The residue was used in the next step without further purification. To a solution of the above amine salt (0.25 mmol) in CH_2_Cl_2_ (2.4 mL) were added (3*S*,4*R*)-statine derivative **13** (117 mg, 0.37 mmol), DIPEA (0.21 mL, 1.20 mmol), HOAt (44 mg, 0.32 mmol), and EDC·HCl (95 mg, 0.50 mmol) at room temperature. After stirring for 12 h, the reaction mixture was quenched with 1N HCl and extracted with CH_2_Cl_2_. The combined organic layer was dried over MgSO_4_ and concentrated in vacuo. The residue was purified by flash column chromatography on silica gel (EtOAc/*n*-hexane = 1:1) to afford pentamer **31** (143 mg, 75% for 2 steps) as white solid. [α${]}_{D}^{20}$= +72.55 (c 0.20, CHCl_3_); ^1^H NMR (800 MHz, CDCl_3_, a mixture of rotamers) Major rotamer δ 7.23–7.19 (m, 4H), 7.16 (t, *J* = 7.2 Hz, 1H), 6.74 (s, 1H), 6.49 (dd, *J* = 102.1, 7.2 Hz, 1H), 5.66 (m, 1H), 4.77–4.72 (m, 1H), 4.53 (d, *J* = 8.0 Hz, 1H), 4.45–4.42 (m, 1H), 4.22–4.18 (m, 1H), 3.84–3.80 (m, 1H), 3.72 (s, 3H), 3.64–3.59 (m, 1H), 3.49–3.43 (m, 1H), 3.24–3.18 (m, 2H), 2.99 (s, 3H), 2.97–2.93 (m, 1H), 2.38–2.32 (m, 2H), 2.22–2.19 (m, 1H), 1.93–1.90 (m, 1H), 1.85–1.81 (m, 3H), 1.65–1.61 (m, 2H), 1.54–1.49 (m, 2H), 1.45 (s, 3H), 1.42 (s, 9H), 1.29–1.26 (m, 2H), 1.12–1.10 (m, 1H), 0.91 (d, *J* = 6.7 Hz, 3H), 0.88 (d, *J* = 6.5 Hz, 3H), 0.87 (d, *J* = 7.2 Hz, 3H), 0.85 (t, *J* = 7.6 Hz, 3H), 0.83 (d, *J* = 6.9 Hz, 3H); ^13^C NMR (200 MHz, CDCl_3_, a mixture of rotamers) Major rotamer δ 172.4, 172.1, 170.0, 168.1, 168.1, 156.7, 136.7, 129.5 (2C), 128.3 (2C), 126.7, 92.7, 80.0, 72.1, 59.3, 57.8, 55.6, 53.3, 52.2, 47.0, 45.1, 39.4, 38.9, 37.3, 34.7, 30.3, 29.7, 28.8, 28.4 (4C), 25.3, 24.8, 23.6, 21.5, 17.6, 15.4, 14.1, 11.4; IR (thin film, neat) ν_max_ 2926, 1635, 1366, 1175, 781 cm^−1^; LR-MS (FAB+) m/z 732 (M − C(CH_3_)_2_ + 3H)^+^; HR-MS (FAB+) calcd for C_38_H_62_N_5_O_9_ (M − C(CH_3_)_2_ + 3H)^+^ 732.4548; found 732.4526.

(28*R*, 29*S*)-Izenamide B variant (**4**). To a solution of pentamer **29** (41 mg, 0.05 mmol) in CH_2_Cl_2_ (0.8 mL) was added TFA (0.2 mL) dropwise at room temperature. After stirring for 1 h, the reaction mixture was concentrated in vacuo. The residue was used in the next step without further purification. To a solution of the above amine salt (0.05 mmol) in CH_2_Cl_2_ (1.0 mL) were added acid **9** (27 mg, 0.05 mmol), DIPEA (0.05 mL, 0.30 mmol), HOAt (9 mg, 0.07 mmol) and EDC·HCl (20 mg, 0.10 mmol) at room temperature. After stirring for 12 h, the reaction mixture was quenched with 1*N* HCl and extracted with CH_2_Cl_2_. The combined organic layer was dried over MgSO_4_ and concentrated in vacuo. The residue was filtered through a short column of silica gel. To a solution of the above heptamer (0.05 mmol) in THF (1.0 mL) was added TBAF (1 M in THF, 0.20 mL, 0.20 mmol) at room temperature. After stirring for 6 h, the reaction mixture was quenched with 1*N* HCl and extracted with CH_2_Cl_2_. The combined organic layer was dried over MgSO_4_ and concentrated in vacuo. The residue was purified by flash column chromatography on silica gel (acetone/*n*-hexane = 1:3) to afford (28*R*, 29*S*)-izenamide B variant **4** (27 mg, 61% for 3 steps) as white solid. [α${]}_{D}^{20}$= +3.07 (c 1.00, MeOH); ^1^H NMR (800 MHz, CD_3_OD, a mixture of rotamers) Major rotamer δ 7.23–7.21 (m, 4H), 7.16–7.15 (m, 1H), 5.70 (dd, *J* = 10.0, 5.9 Hz, 1H), 4.95 (d, *J* = 4.1 Hz, 1H), 4.69 (q, *J* = 7.0 Hz, 1H), 4.42-4.40 (m, 1H), 4.20 (d, *J* = 6.6 Hz, 1H), 4.11 (d, *J* = 4.3 Hz, 1H), 3.90 (ddd, *J* = 11.5, 7.0, 2.9 Hz, 1H), 3,79 (ddd, *J* = 9.9, 7.1, 2.9 Hz, 1H), 3.71 (s, 3H), 3.47–3.44 (m, 1H), 3.38–3.35 (m, 1H), 3.11 (dd, *J* = 14.3, 5.8 Hz, 1H), 3.06 (s, 3H), 2.93 (dd, *J* = 14.3, 10.0 Hz, 1H), 2.44 (dd, *J* = 14.3, 2.9 Hz, 1H), 2.35 (dd, *J* = 14.3, 9.6 Hz, 1H), 2.27–2.24 (m, 1H), 2.11–2.07 (m, 1H), 1.98–1.95 (m, 1H), 1.92–1.90 (m, 1H), 1.86–1.85 (m, 1H), 1.82–1.80 (m, 1H), 1.57–1.51 (m, 2H), 1.46–1.43 (m, 4H), 1.33–1.29 (m, 4H), 1.17–1.14 (m, 1H), 1.01 (d, *J* = 6.9 Hz, 3H), 0.97 (d, *J* = 6.8 Hz, 3H), 0.94 (d, *J* = 7.4 Hz, 3H), 0.93 (d, *J* = 6.5 Hz, 3H), 0.90 (d, *J* = 6.8 Hz, 3H), 0.89 (d, *J* = 7.0 Hz, 3H), 0.87 (t, *J* = 7.4 Hz, 3H), 0.82 (d, *J* = 7.0 Hz, 3H); ^13^C NMR (200 MHz, CD_3_OD, a mixture of rotamers) Major rotamer *δ* 175.3, 174.7, 174.6, 173.9, 173.2, 172.0, 170.3, 138.3, 130.8 (2C), 129.3 (2C), 127.6, 77.9, 76.5, 73.1, 61.0, 59.2, 57.2, 53.1, 52.6, 46.4, 41.3, 40.2, 38.6, 38.0, 35.6, 33.3, 31.1, 30.4, 30.0, 27.3, 26.2, 26.0, 25.7, 24.3, 21.6, 19.4, 17.0, 16.9, 16.0, 14.7, 11.9, 11.7; IR (thin film, neat) ν_max_ 3646, 3307, 2925, 2858, 2360, 1746, 1643, 1456, 1054, 1016, 850 cm^−1^; LR-MS (FAB+) m/z 846 (M + H)^+^; HR-MS (FAB+) calcd for C_44_H_72_N_5_O_11_ (M + H)^+^ 846.5228; found 846.5220.

(28*R*, 29*R*)-Izenamide B variant (**5**). To a solution of pentamer **30** (50 mg, 0.06 mmol) in CH_2_Cl_2_ (0.8 mL) was added TFA (0.2 mL) dropwise at room temperature. After stirring for 1 h, the reaction mixture was concentrated in vacuo. The residue was used in the next step without further purification. To a solution of the above amine salt (0.06 mmol) in CH_2_Cl_2_ (1.0 mL) were added acid **9** (33 mg, 0.06 mmol), DIPEA (0.06 mL, 0.30 mmol), HOAt (11 mg, 0.08 mmol), and EDC·HCl (25 mg, 0.13 mmol) at room temperature. After stirring for 12 h, the reaction mixture was quenched with 1*N* HCl and extracted with CH_2_Cl_2_. The combined organic layer was dried over MgSO_4_ and concentrated in vacuo. The residue was filtered through a short column of silica gel. To a solution of the above heptamer (0.06 mmol) in THF (1.0 mL) was added TBAF (1 M in THF, 0.20 mL, 0.20 mmol) at room temperature. After stirring for 6 h, the reaction mixture was quenched with 1*N* HCl and extracted with CH_2_Cl_2_. The combined organic layer was dried over MgSO_4_ and concentrated in vacuo. The residue was purified by flash column chromatography on silica gel (acetone/*n*-hexane = 1:3) to afford (28*R*, 29*R*)-izenamide B variant **5** (35 mg, 63% for 3 steps) as a white solid. [α${]}_{D}^{20}$= −40.20 (*c* 0.25, MeOH); ^1^H NMR (800 MHz, CD_3_OD, a mixture of rotamers) Major rotamer *δ* 7.24–7.21 (m, 4H), 7.16–7.15 (m, 1H), 5.68 (dd, *J* = 9.9, 6.0 Hz, 1H), 4.89 (m, 1H), 4.66 (q, *J* = 7.0 Hz, 1H), 4.42-4.40 (m, 1H), 4.20 (d, *J* = 6.8 Hz, 1H), 4.05 (d, *J* = 2.7 Hz, 1H), 3.99-3.96 (m, 2H), 3.71 (s, 3H), 3.47-3.44 (m, 1H), 3.40-3.38 (m, 1H), 3.11 (dd, *J* = 14.3, 5.9 Hz, 1H), 3.06 (s, 3H), 2.93 (dd, *J* = 14.3, 10.0 Hz, 1H), 2.35-2.29 (m, 2H), 2.27-2.24 (m, 1H), 1.97-1.93 (m, 1H), 1.87-1.85 (m, 3H), 1.82-1.78 (m, 1H), 1.63-1.59 (m, 1H), 1.58-1.54 (m, 1H), 1.52-1.42 (m, 3H), 1.35-1.31 (m, 5H), 1.18-1.14(m, 1H), 0.95 (d, *J* = 7.5 Hz, 3H), 0.93 (d, *J* = 6.7 Hz, 6H), 0.91 (d, *J* = 6.6 Hz, 3H), 0.89 (d, *J* = 6.8 Hz, 3H), 0.87 (t, *J* = 7.5 Hz, 3H), 0.83 (d, *J* = 7.0 Hz, 3H), 0.80 (d, *J* = 6.9 Hz, 3H); ^13^C NMR (200 MHz, CD_3_OD, a mixture of rotamers) Major rotamer *δ* 176.9, 174.6, 174.0, 173.9, 173.5, 173.2, 170.3, 138.3, 130.8 (2C), 129.3 (2C), 127.6, 74.8, 71.4, 60.9, 59.0, 57.2, 52.6, 52.0, 48.5, 46.5, 42.0, 41.9, 39.3 (2C), 38.2, 35.6 (2C), 31.1, 30.0, 27.4, 26.2, 25.9, 25.7, 23.7, 22.3, 16.8, 16.0, 13.5 (2C), 12.2 (2C), 11.6; IR (thin film, neat) *ν*_max_ 3311, 2922, 2853, 1633, 1573, 1461, 1261, 1138, 802, 676 cm^−1^; LR-MS (FAB+) *m/z* 846 (M + H)^+^; HR-MS (FAB+) calcd for C_44_H_72_N_5_O_11_ (M + H)^+^ 846.5228; found 846.5224.

(28*S*,29*R*)-Izenamide B variant (**6**). To a solution of pentamer **31** (45 mg, 0.06 mmol) in CH_2_Cl_2_ (0.8 mL) was added TFA (0.2 mL) dropwise at room temperature. After stirring for 1 h, the reaction mixture was concentrated in vacuo. The residue was used in the next step without further purification. To a solution of the above amine salt (0.06 mmol) in CH_2_Cl_2_ (1.0 mL) were added acid **9** (30 mg, 0.06 mmol), DIPEA (0.05 mL, 0.30 mmol), HOAt (10 mg, 0.08 mmol), and EDC·HCl (22 mg, 0.11 mmol) at room temperature. After stirring for 12 h, the reaction mixture was quenched with 1*N* HCl and extracted with CH_2_Cl_2_. The combined organic layer was dried over MgSO_4_ and concentrated in vacuo. The residue was filtered through a short column of silica gel. To a solution of the above heptamer (0.06 mmol) in THF (1.0 mL) was added TBAF (1 M in THF, 0.20 mL, 0.20 mmol) at room temperature. After stirring for 6 h, the reaction mixture was quenched with 1*N* HCl and extracted with CH_2_Cl_2_. The combined organic layer was dried over MgSO_4_ and concentrated in vacuo. The residue was purified by flash column chromatography on silica gel (acetone/*n*-hexane = 1:3) to afford (28*S*,29*R*)-izenamide B variant **6** (30 mg, 61% for 3 steps) as a white solid. [α${]}_{D}^{20}$= +7.53 (*c* 1.00, MeOH); ^1^H NMR (800 MHz, CD_3_OD, a mixture of rotamers) Major rotamer *δ* 7.24–7.20 (m, 4H), 7.16–7.14 (m, 1H), 5.68 (dd, *J* = 9.9, 6.0 Hz, 1H), 5.00 (d, *J* = 4.0 Hz, 0.5H), 4.86 (overlapped, 0.5H), 4.66-4.63 (m, 1H), 4.41 (dd, *J* = 8.1, 6.9 Hz, 1H), 4.20 (dd, *J* = 7.2, 5.3 Hz, 1H), 4.17 (d, *J* = 4.1 Hz, 0.5H), 4.01 (d, *J* = 2.8 Hz, 0.5H), 3.94–3.92 (m, 1H), 3.86 (ddd, *J* = 9.4, 6.1, 3.5 Hz, 0.5H), 3.77 (ddd, *J* = 9.7, 6.8, 3.0 Hz, 0.5H), 3.71 (s, 3H), 3.48–3.45 (m, 1H), 3.42–3.37 (m, 1H), 3.11 (dd, *J* = 14.3, 5.9 Hz, 1H), 3.05 (d, *J* = 1.5 Hz, 3H), 2.93 (dd, *J* = 14.2, 10.0 Hz, 1H), 2.39–2.34 (m, 1H), 2.33–2.31 (m, 0.5H), 2.28–2.24 (m, 1.5H), 2.13–2.10 (m, 0.5H), 2.04–1.99 (m, 0.5H), 1.94–1.91 (m, 1.5H), 1.88–1.83 (m, 2.5H), 1.79–1.72 (m, 1.5H), 1.67–1.57 (m, 1.5H), 1.50-1.41(m, 5H), 1.33–1.26 (m, 2H), 1.16-1.11 (m, 1H), 1.02 (d, *J* = 6.9 Hz, 1.5H), 0.96 (t, *J* = 6.6 Hz, 3H), 0.94-0.92 (m, 4.5H), 0.90 (dd, *J* = 6.9, 2.5 Hz, 3H), 0.88 (d, *J* = 6.7 Hz, 3H), 0.87-0.85 (m, 4.5H), 0.83 (dd, *J* = 7.0, 1.8 Hz, 3H), 0.80 (d, *J* = 6.9 Hz, 1.5H); ^13^C NMR (200 MHz, CD_3_OD, a mixture of rotamers) Major rotamer *δ* 176.9, 175.3, 174.5, 174.0, 173.2, 172.1, 170.3, 138.3, 130.8 (2C), 129.3 (2C), 127.6, 78.0, 76.7, 74.9, 72.5, 60.9, 58.8, 57.2, 52.6, 46.5, 41.1, 40.0, 39.3, 38.3, 35.6, 33.2, 31.0, 30.0, 27.4, 26.2, 25.9, 25.7, 24.2, 21.7, 19.5, 16.8, 16.7, 15.9, 14.8, 12.2, 12.0, 11.5; IR (thin film, neat) *ν*_max_ 3326, 2961, 1747, 1639, 1530, 1455, 1176, 1033, 700 cm^−1^; LR-MS (FAB+) *m/z* 846 (M + H)^+^; HR-MS (FAB+) calcd for C_44_H_72_N_5_O_11_ (M + H)^+^ 846.5228; found 846.5230.

Boc-l-Ile-d-Ala-NMe-d-Phe-OMe (**20**). To a solution of Boc-*N*Me-d-Phe-OMe [27] (660 mg, 2.30 mmol) in CH_2_Cl_2_ (9.0 mL) was added TFA (1.5 mL) dropwise at room temperature. After stirring for 1 h, the reaction mixture was concentrated in vacuo. The residue was used in the next step without further purification. To a solution of the above amine salt (2.30 mmol) in CH_2_Cl_2_ (11 mL) were added Boc-l-Ile-l-Ala-OH **32** (816 mg, 2.70 mmol), DIPEA (1.2 mL, 6.90 mmol) and DEPBT (1.30 g, 4.34 mmol) at 0 °C. After stirring for 12 h at the same temperature, the reaction mixture was quenched with 1*N* HCl and extracted with CH_2_Cl_2_. The combined organic layer was dried over MgSO_4_ and concentrated in vacuo. The residue was purified by flash column chromatography on silica gel (EtOAc/*n*-hexane = 1:1) to afford Boc-l-Ile-d-Ala-*N*Me-d-Phe-OMe tripeptide **20** (218 mg, 20% for 2 steps) as white solid. [α${]}_{D}^{20}$= +48.23 (c 1.00, CHCl_3_); ^1^H NMR (800 MHz, CDCl_3_, a mixture of rotamers) Major rotamer *δ* 7.25 (t, *J* = 7.5 Hz, 2H), 7.19 (t, *J* = 7.4 Hz, 1H), 7.14 (d, *J* = 7.3 Hz, 2H), 6.65 (d, *J* = 5.6 Hz, 1H), 5.05 (dd, *J* = 10.9, 5.3 Hz, 1H), 5.00 (d, *J* = 8.0 Hz, 1H), 4.73 (quint, *J* = 6.8 Hz, 1H), 3.99-3.94 (m, 1H), 3.71 (s, 3H), 3.36 (dd, *J* = 14.7, 5.3 Hz, 1H), 3.05 (dd, *J* = 14.7, 10.9 Hz, 1H), 2.82 (s, 3H), 1.84-1.80 (m, 1H), 1.42 (s, 9H), 1.39-1.33 (m, 1H), 1.25 (d, *J* = 6.8 Hz, 3H), 1.08-1.02 (m, 1H), 0.87 (d, *J* = 7.0 Hz, 3H), 0.87 (t, *J* = 7.3 Hz, 3H); ^13^C NMR (200 MHz, CDCl_3_, a mixture of rotamers) Major rotamer *δ* 172.5, 170.8, 170.3, 136.7, 128.8 (2C), 128.6 (2C), 126.9, 79.8, 59.5, 59.0, 52.4, 45.5, 37.9, 34.3, 33.4, 28.3 (3C), 28.3, 24.7, 18.2, 15.5, 11.7; IR (thin film, neat) ν_max_ 3308, 2967, 1743, 1714, 1638, 1497, 1366, 1172, 1017, 750 cm^-1^; LR-MS (ESI+) *m/z* 478 (M + H)^+^; HR-MS (ESI+) calcd for C_25_H_40_N_3_O_6_ (M + H)^+^ 478.2912; found 478.2915.

Boc-l-Ile-d-Ala-NMe-d-Phe-l-Pro-OMe (**15**). To a solution Boc-l-Ile-d-Ala-*N*Me-d-Phe-OMe **20** (210 mg, 0.44 mmol) in a mixture of THF, MeOH and H_2_O (1:1:1, 4 mL) was added LiOH·H_2_O (34 mg, 0.81 mmol) at room temperature. After stirring for 2 h, the reaction mixture was quenched with 1*N* HCl and extracted with EtOAc. The combined organic layer was dried over MgSO_4_ and concentrated in vacuo. The residue was used in the next step without further purification. To a solution of the above acid (0.44 mmol) in CH_2_Cl_2_ (4 mL) were added l-Pro-OMe·HCl **22** (81 mg, 0.49 mmol), DIPEA (0.23 mL, 1.3 mmol), and DEPBT (615 mg, 2.06 mmol) at 0 °C. After stirring for 12 h at the same temperature, the reaction mixture was quenched with 1*N* HCl and extracted with CH_2_Cl_2_. The combined organic layer was dried over MgSO_4_ and concentrated in vacuo. The residue was purified by flash column chromatography on silica gel (EtOAc/*n*-hexane =1:1) to give Boc-l-Ile-d-Ala-*N*Me-d-Phe-l-Pro-OMe tetrapeptide **15** (170 mg, 76% for 2 steps) as white solid. [α${]}_{D}^{20}$= +37.07 (*c* 1.00, CHCl_3_); ^1^H NMR (800 MHz, CDCl_3_, a mixture of rotamers) Major rotamer *δ* 7.23-7.21 (m, 3H), 7.18-7.14 (m, 2H), 6.63 (dd, *J* = 14.4, 6.7 Hz, 1H), 5.53 (dd, *J* = 8.0, 6.9 Hz, 1H), 5.02 (d, *J* = 8.2 Hz, 1H), 4.83-4.80 (m, 1H), 4.40 (dd, *J* = 8.5, 5.5 Hz, 1H), 3.96-3.90 (m, 1H), 3.68 (s, 3H), 3.59-3.51 (m, 1H), 3.39-3.34 (m, 1H), 3.29 (dt, *J* = 10.5, 6.1 Hz, 1H), 3.27 (dd, *J* = 13.7, 8.5 Hz, 1H), 3.02 (s, 3H), 2.77 (dd, *J* = 13.6, 6.5 Hz, 1H), 2.16-2.09 (m, 1H), 1.91-1.86 (m, 1H), 1.85-1.76 (m, 3H), 1.42 (s, 9H), 1.29 (d, *J* = 6.8 Hz, 3H), 1.11-0.99 (m, 1H), 0.88 (d, *J* = 6.7 Hz, 3H), 0.87 (t, *J* = 6.6 Hz, 3H); ^13^C NMR (200 MHz, CDCl_3_, a mixture of rotamers) Major rotamer *δ* 172.5, 172.1, 170.3, 167.8, 155.6, 137.0, 129.5 (2C), 128.3 (2C), 126.6, 79.8, 59.2, 58.8, 56.1, 52.2, 46.6, 45.6, 37.8, 34.7, 30.5, 28.8, 28.3 (3C), 25.0, 18.5, 17.5, 15.5, 11.6; IR (thin film, neat) ν_max_ 3298, 2969, 1748, 1712, 1637, 1497, 1365, 1173, 1044, 870 cm^-1^; LR-MS (ESI+) *m/z* 576 (M + H)^+^; HR-MS (ESI+) calcd for C_30_H_47_N_4_O_7_ (M + H)^+^ 576.3471; found 576.3473.

(28*R*,29*S*)-Statine derivative-l-Ile-Gly-NMe-d-Phe-l-Pro-OMe, Pentamer (**33**). To a solution of Boc-NMe-l-Ile-Gly-NMe-d-Phe-OMe **14** (140 mg, 0.25 mmol) in CH_2_Cl_2_ (1.6 mL) was added TFA (0.4 mL) dropwise at room temperature. After stirring for 1 h, the reaction mixture was concentrated in vacuo. The residue was used in the next step without further purification. To a solution of the above amine salt (0.25 mmol) in CH_2_Cl_2_ (2.4 mL) were added (3*S*,4*S*)-natural statine **10** (118 mg, 0.37 mmol), DIPEA (0.2 mL, 1.14 mmol), HOAt (44 mg, 0.32 mmol), and EDC·HCl (95 mg, 0.50 mmol) at room temperature. After stirring for 12 h, the reaction mixture was quenched with 1N HCl and extracted with CH_2_Cl_2_. The combined organic layer was dried over MgSO_4_ and concentrated in vacuo. The residue was purified by flash column chromatography on silica gel (EtOAc/*n*-hexane = 1:1) to afford pentamer **33** 153 mg (81% for 2 steps) as white solid. [α${]}_{D}^{20}$= +54.65 (c 0.20, CHCl_3_); ^1^H NMR (800 MHz, CDCl_3_, a mixture of rotamers) Major rotamer δ 7.24–7.21 (m, 2H), 7.20–7.19 (m, 2H), 7.16 (t, *J* = 7.1 Hz, 1H), 6.72–6.68 (m, 2H), 5.55 (t, *J* = 7.6 Hz, 1H), 4.40–4.37 (m, 2H), 4.22–4.20 (m, 1H), 4.06 (dd, *J* = 17.6, 4.4 Hz, 1H), 3.85 (dd, *J* = 18.9, 4.0 Hz, 1H), 3.70 (s, 3H), 3.64–3.58 (m, 1H), 3.37–3.31 (m, 2H), 3.25 (dd, *J* = 13.7, 8.0 Hz, 1H), 2.95 (s, 3H), 2.80 (dd, *J* = 13.7, 7.1 Hz, 1H), 2.61–2.55 (m, 1H), 2.46 (d, *J* = 14.4 Hz, 1H), 2.14–2.11 (m, 1H), 1.95–1.89 (m, 2H), 1.87–1.78 (m, 3H), 1.75-1.65 (m, 3H), 1.65–1.56 (m, 5H), 1.55–1.52 (m, 1H), 1.46 (s, 9H), 1.12–1.07 (m, 1H), 0.90 (d, *J* = 6.9 Hz, 3H), 0.90–0.89 (m, 6H), 0.88 (t, *J* = 7.5 Hz, 3H); ^13^C NMR (200 MHz, CDCl_3_, a mixture of rotamers) Major rotamer δ 172.5, 170.8, 170.0, 167.9, 167.7, 151.6, 136.9, 129.4 (2C), 128.4 (2C), 126.7, 94.6, 79.9, 68.1, 60.9, 58.9, 57.5, 56.2, 52.2, 46.8, 41.2, 37.4, 35.0, 29.6, 28.8, 28.5 (6C), 25.4, 25.0, 24.7, 24.0 (2C), 21.3, 15.6, 11.5; IR (thin film, neat) ν_max_ 3309, 2958, 1747, 1634, 1388, 1254, 1175, 1088, 701 cm^−1^; LR-MS (ESI+) *m*/*z* 758 (M + H)^+^; HR-MS (ESI+) calcd for C_40_H_64_N_5_O_9_ (M + H)^+^ 758.4704; found 758.4713.

(28*R*,29*S*)-Statine derivative-l-Ile-d-Ala-NMe-d-Phe-l-Pro-OMe, Pentamer (**34**), To a solution of tetrapeptide **15** (140 mg, 0.24 mmol) in CH_2_Cl_2_ (1.6 mL) was added TFA (0.4 mL) dropwise at room temperature. After stirring for 1 h, the reaction mixture was concentrated in vacuo. The residue was used in the next step without further purification. To a solution of the above amine salt (0.24 mmol) in CH_2_Cl_2_ (2.4 mL) were added (3*S*,4*S*)-natural statine **10** (100 mg, 0.32 mmol), DIPEA (0.2 mL, 1.14 mmol), HOAt (43 mg, 0.32 mmol), and EDC·HCl (93 mg, 0.49 mmol) at room temperature. After stirring for 12 h, the reaction mixture was quenched with 1N HCl and extracted with CH_2_Cl_2_. The combined organic layer was dried over MgSO_4_ and concentrated in vacuo. The residue was purified by flash column chromatography on silica gel (EtOAc/*n*-hexane = 1:1) to give pentamer **34** (143 mg, 76% for 2 steps) as white solid. [α${]}_{D}^{20}$= +23.40 (c 0.20, CHCl_3_); ^1^H NMR (800 MHz, CDCl_3_, a mixture of rotamers) Major rotamer δ 7.24-7.21 (m, 4H), 7.18-7.16 (m, 1H), 6.69 (d, *J* = 6.2 Hz, 1H), 6.42 (d, *J* = 6.4 Hz, 1H), 5.52 (dd, *J* = 8.4, 6.5 Hz, 1H), 4.79 (dq, *J* = 6.9, 6.8 Hz, 1H), 4.41 (dd, *J* = 8.6, 5.3 Hz, 1H), 4.32–4.28 (m, 1H), 3.96 (d, *J* = 9.7 Hz, 1H), 3.70 (s, 3H), 3.60–3.53 (m, 1H), 3.35 (dt, *J* = 10.3, 6.2 Hz, 1H), 3.32–3.30 (m, 1H), 3.29 (dd, *J* = 13.6, 8.7 Hz, 1H), 3.05 (s, 3H), 2.78 (dd, *J* = 13.6, 6.4 Hz, 1H), 2.47 (dd, *J* = 14.8, 10.3 Hz, 1H), 2.34 (d, *J* = 15.2 Hz, 1H), 2.15–2.11 (m, 1H), 1.96–1.76 (m, 11H), 1.65–1.60 (m, 1H), 1.55–1.50 (m, 1H), 1.48–1.46 (m, 1H), 1.43 (s, 9H), 1.32 (d, *J* = 6.7 Hz, 3H), 1.15–1.07 (m, 1H), 0.90 (t, *J* = 7.0 Hz, 3H), 0.90 (d, *J* = 6.8 Hz, 9H); ^13^C NMR (200 MHz, CDCl_3_, a mixture of rotamers) Major rotamer δ 172.6 (2C), 172.2, 169.6, 167.8, 156.3, 137.1, 129.6 (2C), 128.4 (2C), 126.6, 93.4, 79.3, 70.5, 58.7, 57.3, 56.3, 52.3, 52.2, 46.6, 45.9, 41.6, 40.1, 37.5, 34.7, 30.9, 30.6, 28.8, 28.4 (4C), 25.1, 25.0, 24.8, 23.1, 22.2, 18.3, 15.4, 11.5; IR (thin film, neat) ν_max_ 3298, 2928, 1647, 1453, 1261, 1199, 800 cm^−1^; LR-MS (FAB+) *m*/*z* 732 (M − C(CH_3_)_2_ + 3H)^+^; HR-MS (FAB+) calcd for C_38_H_62_N_5_O_9_ (M − C(CH_3_)_2_ + 3H)^+^ 732.4548; found 732.4561.

Sta-incorporated-izenamide C (**7**). To a solution of pentamer **33** (56 mg, 0.07 mmol) in CH_2_Cl_2_ (0.8 mL) was added TFA (0.2 mL) dropwise at room temperature. After stirring for 1 h, the reaction mixture was concentrated in vacuo. The residue was used in the next step without further purification. To a solution of the above amine salt (0.07 mmol) in CH_2_Cl_2_ (1.0 mL) were added acid **9** (38 mg, 0.07 mmol), DIPEA (0.063 mL, 0.36 mmol), HOAt (13 mg, 0.10 mmol), and EDC·HCl (28 mg, 0.15 mmol) at room temperature. After stirring for 12 h, the reaction mixture was quenched with 1*N* HCl and extracted with CH_2_Cl_2_. The combined organic layer was dried over MgSO_4_ and concentrated in vacuo. The residue was filtered through a short column of silica gel. To a solution of heptamer (0.07 mmol) in THF (1.0 mL) was added TBAF (1 M in THF, 0.20 mL, 0.20 mmol) at room temperature. After stirring for 6 h, the reaction mixture was quenched by 1*N* HCl and extracted with CH_2_Cl_2_. The combined organic layer was dried over MgSO_4_ and concentrated in vacuo. The residue was purified by flash column chromatograph on silica gel (acetone/*n*-hexane = 1:3) to afford the Sta-introduced izenamide C **7** (38 mg, 62% for 3 steps) as white solid. [α${]}_{D}^{20}$= −28.30 (c 0.20, MeOH); ^1^H NMR (800 MHz, CD_3_OD, a mixture of rotamers) Major rotamer *δ* 7.29–7.24 (m, 1H), 7.22–7.20 (m, 3H), 7.16–7.14 (m, 1H), 5.56 (t, *J* = 7.5 Hz, 1H), 4.96 (d, *J* = 4.2 Hz, 1H), 4.36 (dd, *J* = 8.4, 5.6 Hz, 1H), 4.29 (d, *J* = 6.7 Hz, 1H), 4.12 (d, *J* = 16.8 Hz, 1H), 4.12 (d, *J* = 4.4 Hz, 1H), 4.04–3.98 (m, 2H), 3.89 (d, *J* = 16.9 Hz, 1H), 3.70 (s, 3H), 3.43 (dt, *J* = 10.4, 6.9 Hz, 1H), 3.40–3.37 (m, 1H), 3.20 (dd, *J* = 13.7, 8.0 Hz, 1H), 3.02 (s, 3H), 2.83 (dd, *J* = 13.7, 7.1 Hz, 1H), 2.44–2.35 (m, 2H), 2.21–2.16 (m, 1H), 2.12–2.08 (m, 1H), 1.95–1.88 (m, 3H), 1.87–1.80 (m, 2H), 1.60–1.52 (m, 3H), 1.49-1.43 (m, 1H), 1.34–1.27 (m, 2H), 1.21–1.16 (m, 1H), 1.01 (d, *J* = 6.9 Hz, 3H), 0.98 (d, *J* = 6.9 Hz, 3H), 0.95 (t, *J* = 7.4 Hz, 3H), 0.94 (d, *J* = 7.0 Hz, 3H), 0.92 (d, *J* = 6.4 Hz, 3H), 0.92 (d, *J* = 6.8 Hz, 3H), 0.90 (t, *J* = 7.5 Hz, 3H), 0.89 (d, *J* = 6.5 Hz, 3H); ^13^C NMR (200 MHz, CD_3_OD, a mixture of rotamers) Major rotamer *δ* 176.4, 174.9, 174.7, 174.7, 172.9, 171.1, 171.0, 139.4, 131.3 (2C), 130.2 (2C), 128.4, 79.1, 77.2, 72.2, 61.4, 60.1, 58.6, 53.4, 53.1, 49.1, 42.7, 42.3, 42.2, 39.2, 38.8, 36.5, 34.1, 31.4, 30.7, 28.0, 26.8, 26.8, 26.6, 24.6, 23.0, 20.1, 17.8, 16.8, 15.5, 12.7, 12.5; IR (thin film, neat) ν_max_ 3329, 2965, 1744, 1647, 1456, 1033, 810 cm^−1^; LR-MS (ESI+) *m/z* 758 (M + H)^+^; HR-MS (ESI+) calcd for C_43_H_70_N_5_ O_11_(M + H)^+^ 832.5066; found 832.5079.

18-*epi*-izenamide B (**8**). To a solution of pentamer **34** (48 mg, 0.06 mmol) in CH_2_Cl_2_ (0.8 mL) was added TFA (0.8 mL) dropwise at room temperature. After stirring for 1 h, the reaction mixture was concentrated in vacuo. The residue was used in the next step without further purification. To a solution of the above amine salt (0.06 mmol) in CH_2_Cl_2_ (1.0 mL) were added acid **9** (32 mg, 0.06 mmol), DIPEA (0.05 mL, 0.30 mmol), HOAt (11 mg, 0.08 mmol), and EDC·HCl (24 mg, 0.13 mmol) at room temperature. After stirring for 12 h, the reaction mixture was quenched with 1*N* HCl and extracted with CH_2_Cl_2_. The combined organic layer was dried over MgSO_4_ and concentrated in vacuo. The residue was filtered through a short column of silica gel. To a solution of heptamer (0.06 mmol) in THF (1.0 mL) was added TBAF (1 M in THF, 0.20 mL, 0.20 mmol) at room temperature. After stirring for 6 h, the reaction mixture was quenched 1*N* HCl and extracted with CH_2_Cl_2_. The combined organic layer was dried over MgSO_4_ and concentrated in vacuo. The residue was purified by flash column chromatography on silica gel (acetone/*n*-hexane = 1:3) to give 18-*epi*-izenamide B **8** (33 mg, 63% for 3 steps) as white solid. [α${]}_{D}^{20}$= −52.70 (c 0.10, MeOH); ^1^H NMR (800 MHz, CD_3_OD, a mixture of rotamers) Major rotamer *δ* 7.24–7.20 (m, 4H), 7.17–7.14 (m, 1H), 5.53 (dd, *J* = 9.0, 5.8 Hz, 1H), 4.97 (d, *J* = 4.1 Hz, 1H), 4.83 (q, *J* = 7.0 Hz, 1H), 4.35 (dd, *J* = 8.6, 5.1 Hz, 1H), 4.27 (d, *J* = 6.7 Hz, 1H), 4.13 (d, *J* = 4.5 Hz, 1H), 4.03–3.99 (m, 2H), 3.70 (s, 3H), 3.42–3.38 (m, 1H), 3.36–3.33 (m, 1H), 3.25 (dd, *J* = 13.5, 9.0 Hz, 1H), 3.11 (s, 3H), 2.79 (dd, *J* = 13.5, 5.7 Hz, 1H), 2.42 (qd, *J* = 14.6, 6.8 Hz, 2H), 2.19–2.13 (m, 1H), 2.12–2.07 (m, 1H), 1.97–1.93 (m, 1H), 1.91–1.80 (m, 4H), 1.61–1.55 (m, 2H), 1.52 (ddd, *J* = 13.6, 7.5, 3.5 Hz, 1H), 1.46 (dq, *J* = 6.5, 6.4 Hz, 1H), 1.32 (d, *J* = 6.9 Hz, 3H), 1.32–1.25 (m, 2H), 1.18 (ddd, *J* = 13.6, 9.5, 7.4 Hz, 1H), 1.01 (d, *J* = 6.9 Hz, 3H), 0.97 (d, *J* = 6.8 Hz, 3H), 0.95 (t, *J* = 7.5 Hz, 3H), 0.92 (d, *J* = 6.7 Hz, 3H), 0.92 (d, *J* = 6.9 Hz, 3H), 0.92 (d, *J* = 6.5 Hz, 3H), 0.90 (t, *J* = 7.5 Hz, 3H), 0.88 (d, *J* = 6.5 Hz, 3H); ^13^C NMR (200 MHz, CD_3_OD, a mixture of rotamers) Major rotamer *δ* 176.4, 175.2, 174.9, 174.7, 173.9, 172.7, 170.9, 139.5, 131.3 (2C), 130.2 (2C), 128.4, 79.0, 77.3, 72.3, 61.2, 59.9, 58.7, 53.5, 53.0, 48.9, 48.0, 42.3, 42.1, 39.2, 39.1, 36.4, 34.1, 32.2, 30.7, 28.1, 26.9, 26.6, 26.6, 24.6, 23.0, 20.1, 18.4, 17.9, 16.8, 15.5, 12.7, 12.6; IR (thin film, neat) ν_max_ 3680, 2966, 2844, 1637, 1346, 1055, 1033, 691 cm^−1^; LR-MS (ESI+) *m/z* 846 (M + H)^+^; HR-MS (ESI+) calcd for C_44_H_72_N_5_O_11_ (M + H)^+^ 846.5223; found 846.5230.

### 3.3. Cathepsin D (CTSD) Human Pepsin Aspartic Peptidase Enzymatic Assay

Human liver cathepsin D was used for the enzymatic essay. Test compound or vehicle was preincubated with 190 mU/mL enzyme in modified acetate buffer pH 4.0 for 15 minutes at 37 °C. The reaction was initiated by addition of 20 μM MOCAc-Gly-Lys-Pro-Ile-Leu-Phe-Phe-Arg-Leu-Lys(Dnp)-d-Arg NH_2_ for another 10-minute incubation period. Determination of the amount of MOCAc formed was read spectrofluorimetrically at 340 nm/400. Compounds were screened at 0.5 and 1.0 μM. Cathepsin-D-Human-Pepsin-Aspartic-Peptidase-Enzymatic-Assay-Panlabs (eurofinsdiscovery.com, accessed on 22 February 2022).

## 4. Conclusions

In this study, a range of structural variants of izenamide depsipeptides were designed and synthesized to elucidate core structures responsible for CTSD inhibition. The divergent syntheses of four diastereomers of the Sta unit of izenamide B and evaluation of CTSD inhibition by the izenamide variants were conducted. The results confirmed the essential role of the natural (3*S*,4*S*)-Sta unit of izenamides A (**1**) and B (**2**) in CTSD inhibition. The incorporation of the natural Sta unit into izenamide C (**3**) led us to identify an izenamide C variant (**7**) that exhibited more potent CTSD inhibition than izenamide B (**2**). Moreover, the most potent 18-*epi*-izenamide B (**8**), possessing an unnatural d-Ala in izenamide B (**2**), was identified based on the substituent effects of the alanine unit. These results highlighted the important role of the Ala or Gly unit, in addition to the natural Sta unit of izenamides, in CTSD inhibition. Overall, these findings provide valuable information for the further development of novel depsipeptide-based CTSD inhibitors.

## Figures and Tables

**Table 1:** Cathepsin D inhibitory activities of synthetic izenamides A–C (**1**–**3**) and their variants (**4**–**8**).

Stereochemistry of Sta	Compounds	Inhibition of CTSD (%) ^a^	
		0.5 μM	1.0 μM
natural (28*S*,29*S*)	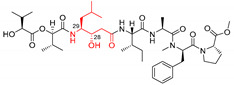 izenamide A (**1**)	10.13	15.55
natural (28*S*,29*S*)	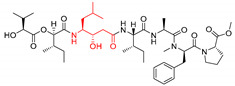 izenamide B (**2**)	8.12	14.76
none	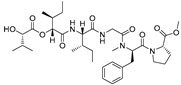 izenamide C (**3**)	-	N.A. ^b^
(28*R*,29*S*)	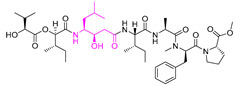 izenamide B variant (**4**)	N.A.	
(28*R*,29*R*)	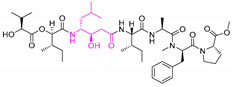 izenamide B variant (**5**)	N.A.	
(28*S*,29*R*)	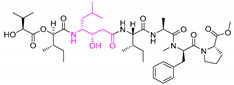 izenamide B variant (**6**)	N.A.	
natural (28*S*,29*S*)	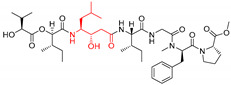 Sta-incorporated izenamide C (**7**)	26.99	36.07
natural (28*S*,29*S*)	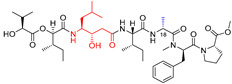 18-*epi*-izenamide B (**8**)	37.29	40.96

^a^ The cathepsin D human pepsin aspartic peptidase enzymatic assay was performed at Eurofins Panlabs Discovery Services (New Taipei City, Taiwan) by measuring the percentage inhibition at two concentration points (0.5 and 1.0 μM), and pepstatin A was used as positive control (IC_50_ 0.00204 μM); experiments were performed in duplicate at the indicated concentrations. ^b^ N.A., not active.

## Data Availability

The original data presented in the study are included in the article/Supplementary Materials.

## Supplementary Materials

The following supporting information can be downloaded at: https://www.mdpi.com/article/10.3390/md21050281/s1, the ^1^H and ^13^C NMR spectra of **24**, **27**, **28**, **29**, **30**, **31**, **4**, **5**, **6**, **20**, **15**, **33**, **34**, **7** and **8**.
