# Supplementary material for: Structural Congeners of Izenamides Responsible for Cathepsin D Inhibition: Insights from Synthesis-Derived Elucidation
Source: Mar Drugs. 2023 Apr 28;21(5):281. doi: 10.3390/md21050281 (PMC10223608; doi:10.3390/md21050281)
Supplement: Supplementary file 1 [file marinedrugs-21-00281-s001.zip › marinedrugs-2361954-supplementary.pdf]

Supporting Information

Structural Congeners of Izenamides Resposible for Cathepsin D Inhibition: Insights from Synthesis-Derived Elucidation

Hyun Su Kim [1], Hyejin Kong [1], Taewoo Kim [1], Changjin Lim [2], Seungbeom Lee [1], Seok-Ho Kim [3] and Young-Ger Suh [1,*]

[1] College of Pharmacy and Institute of Pharmaceutical Sciences, CHA University, 120 Haeryong-ro, Pocheon 11160, Gyeonggi-do, Republic of Korea
[2] School of Pharmacy, Jeonbuk National University, Jeonju 54896, Republic of Korea
[3] College of Pharmacy, Kangwon National University, Chuncheon, Gangwon-do 24341, Republic of Korea

List of Contents

I. ^{1}H NMR and ^{13}C NMR spectra S1-S17

II. Comparison of ^{1}H, ^{13}C NMR spectra of acetonide **24**, **27**, and **28** S18-S19

III. Comparison of ^{1}H, ^{13}C NMR spectra of Izenamide B Variants (**4**-**6**) and (**8**) S20-S22

IV. HRMS spectra of Izenamide Variants (**4**-**8**) S23-S28

I. ^{1}H NMR and ^{13}C NMR spectra

^{1}H NMR and ^{13}C NMR spectra of **24**

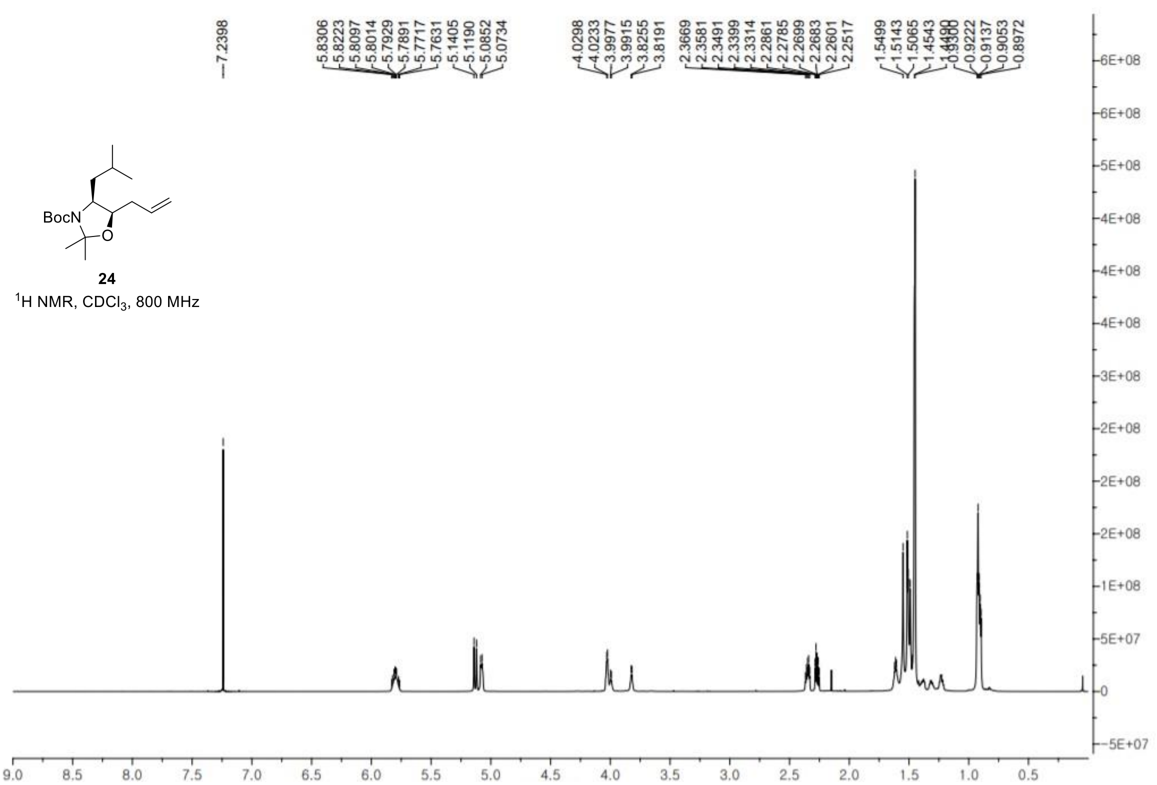

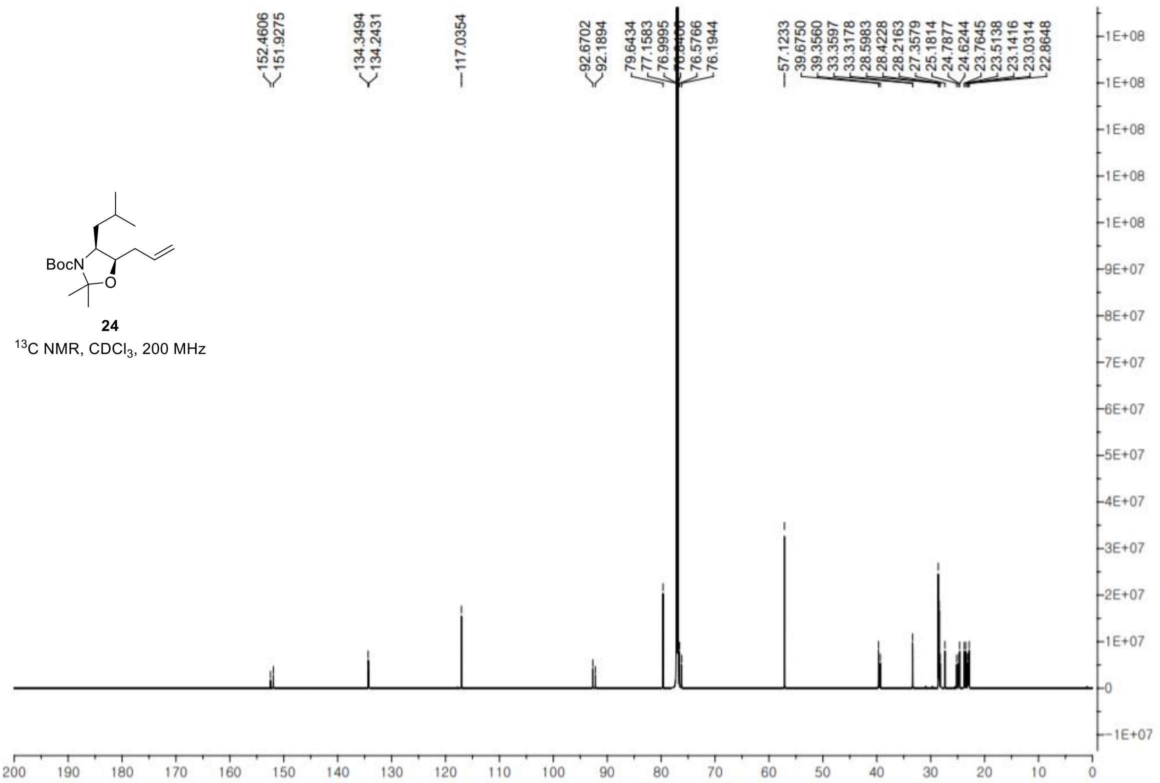

^{1}H NMR and ^{13}C NMR spectra of **25**

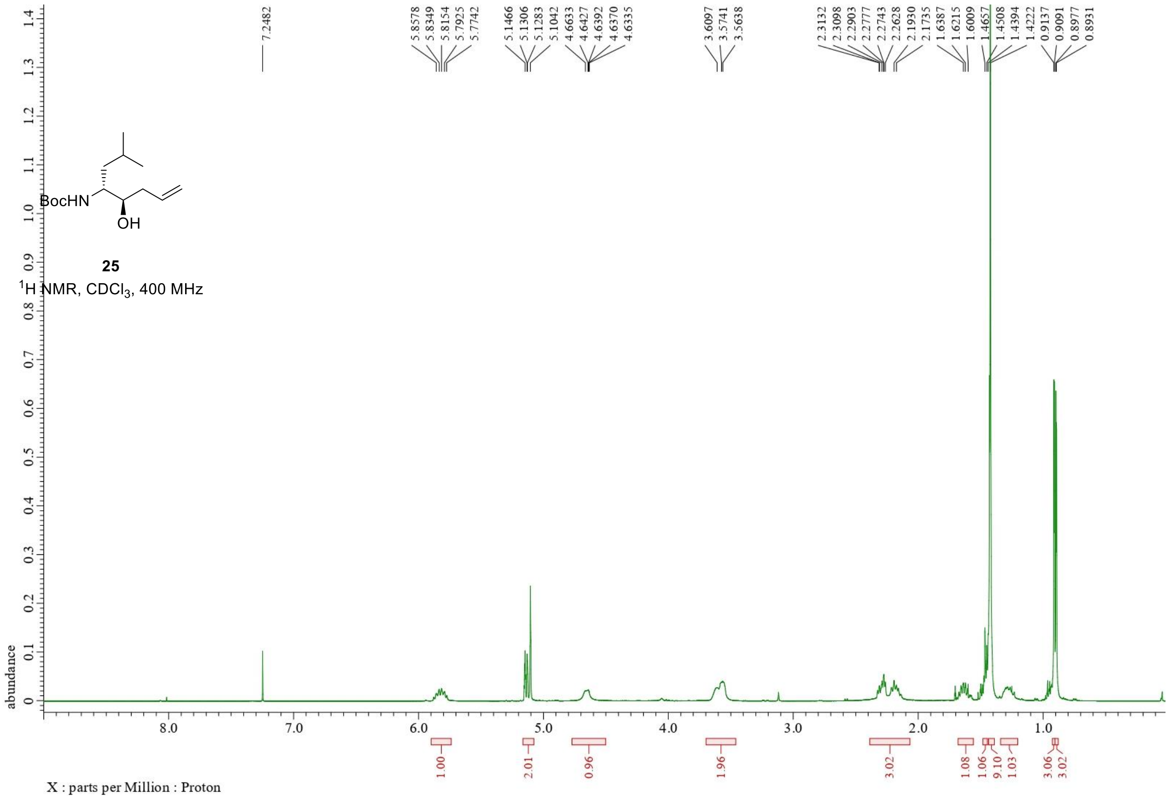

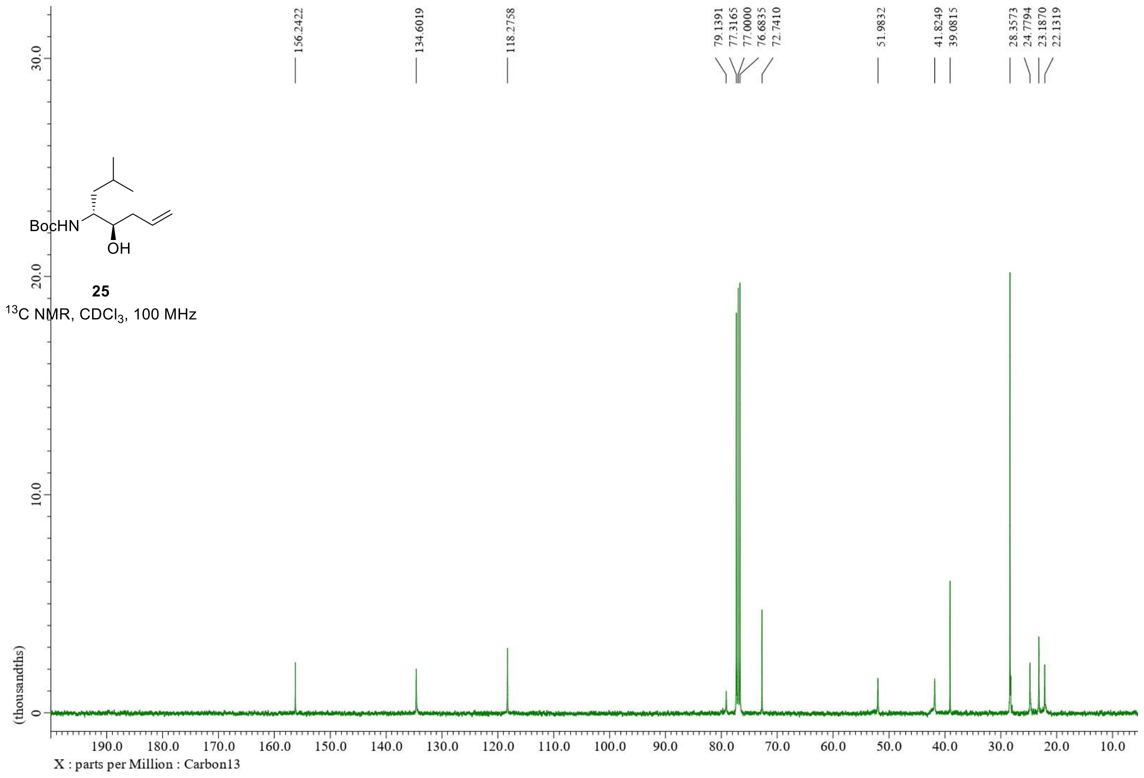

^{1}H NMR and ^{13}C NMR spectra of **26**

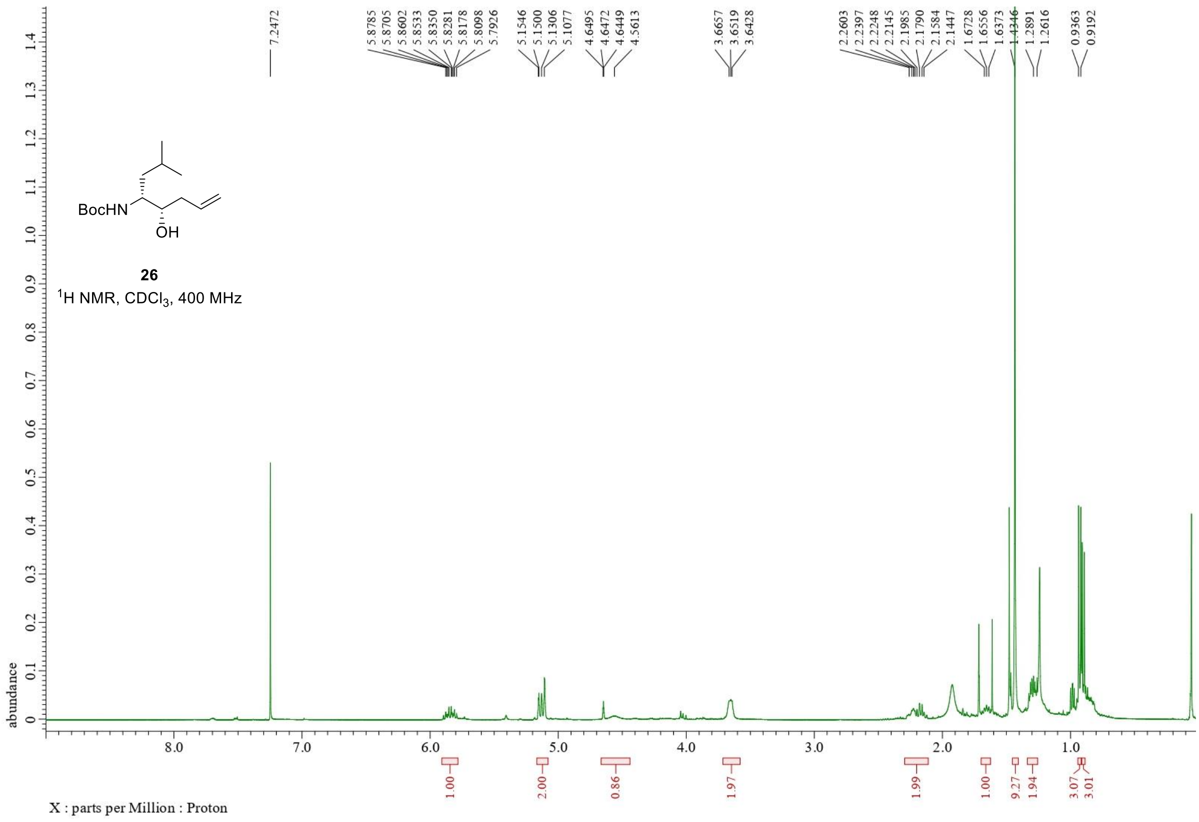

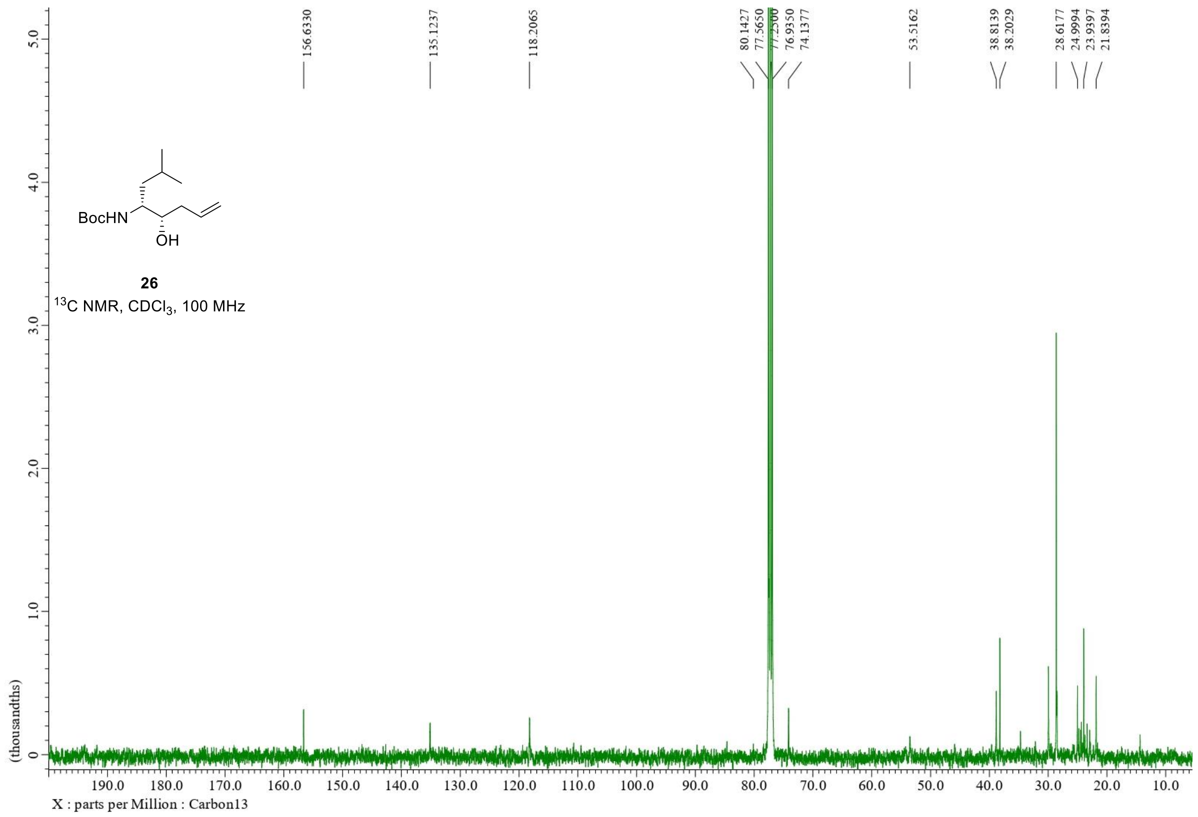

^{1}H NMR and ^{13}C NMR spectra of **27**

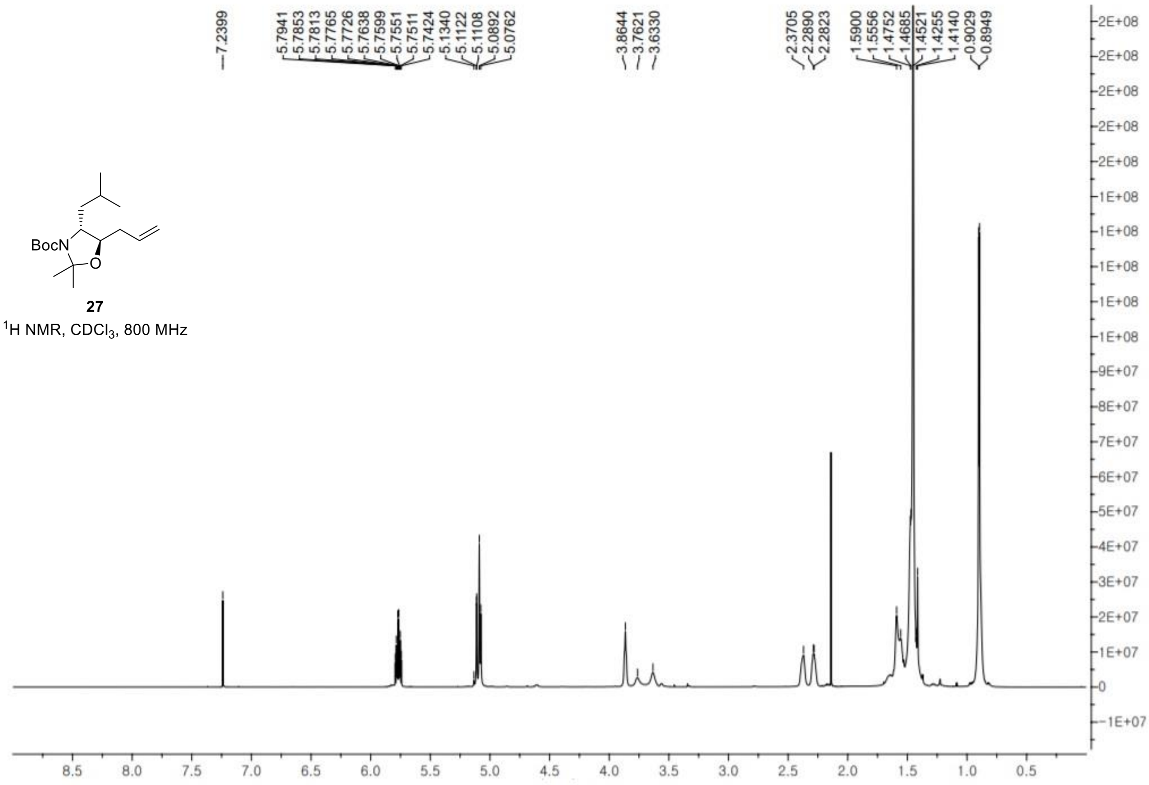

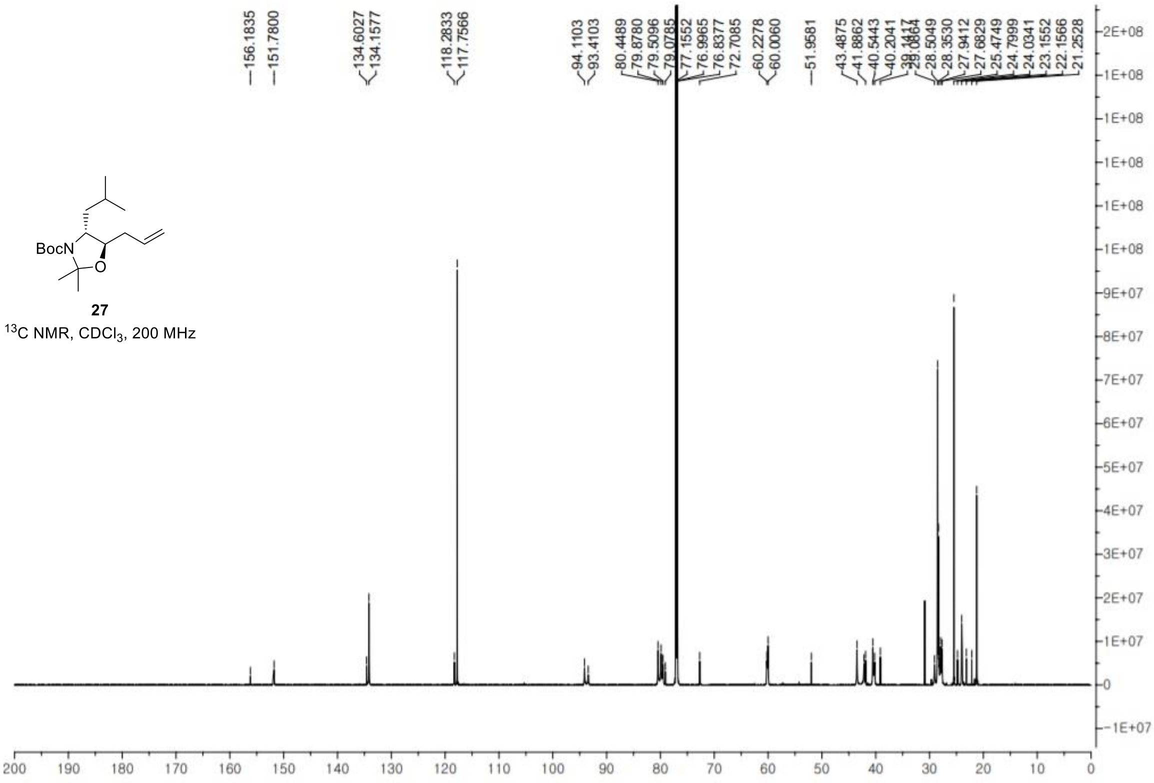

^{1}H NMR and ^{13}C NMR spectra of **28**

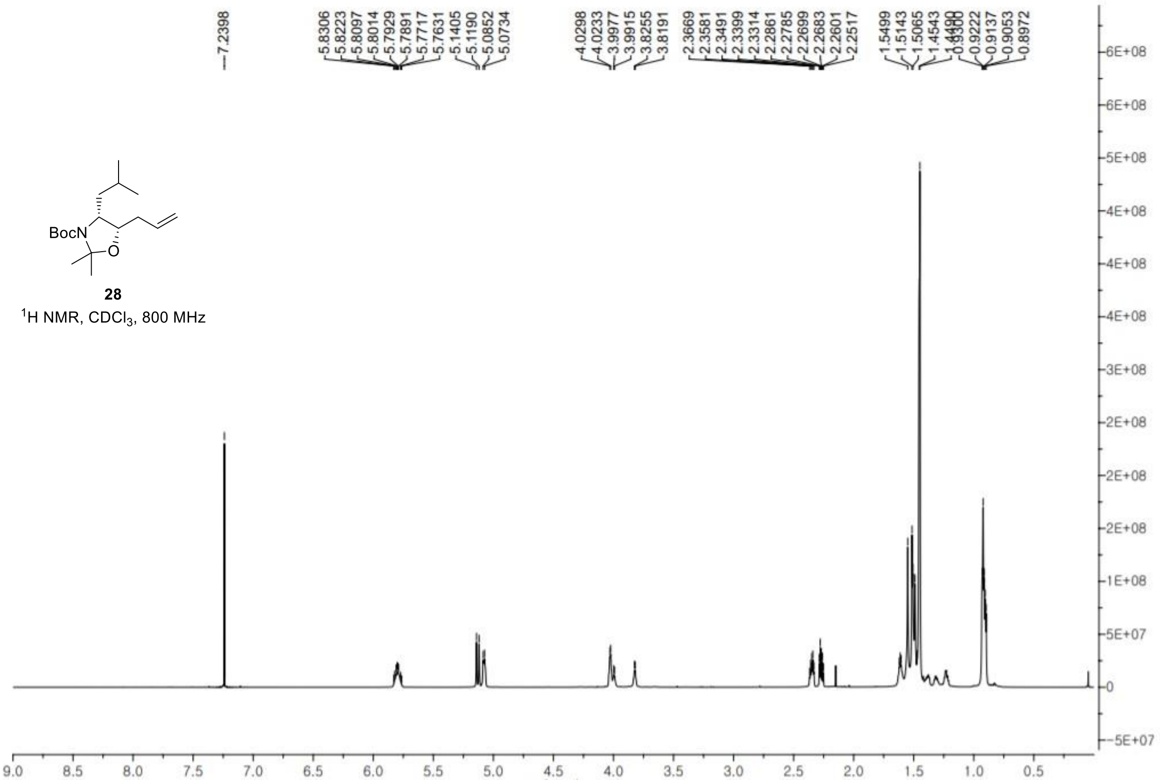

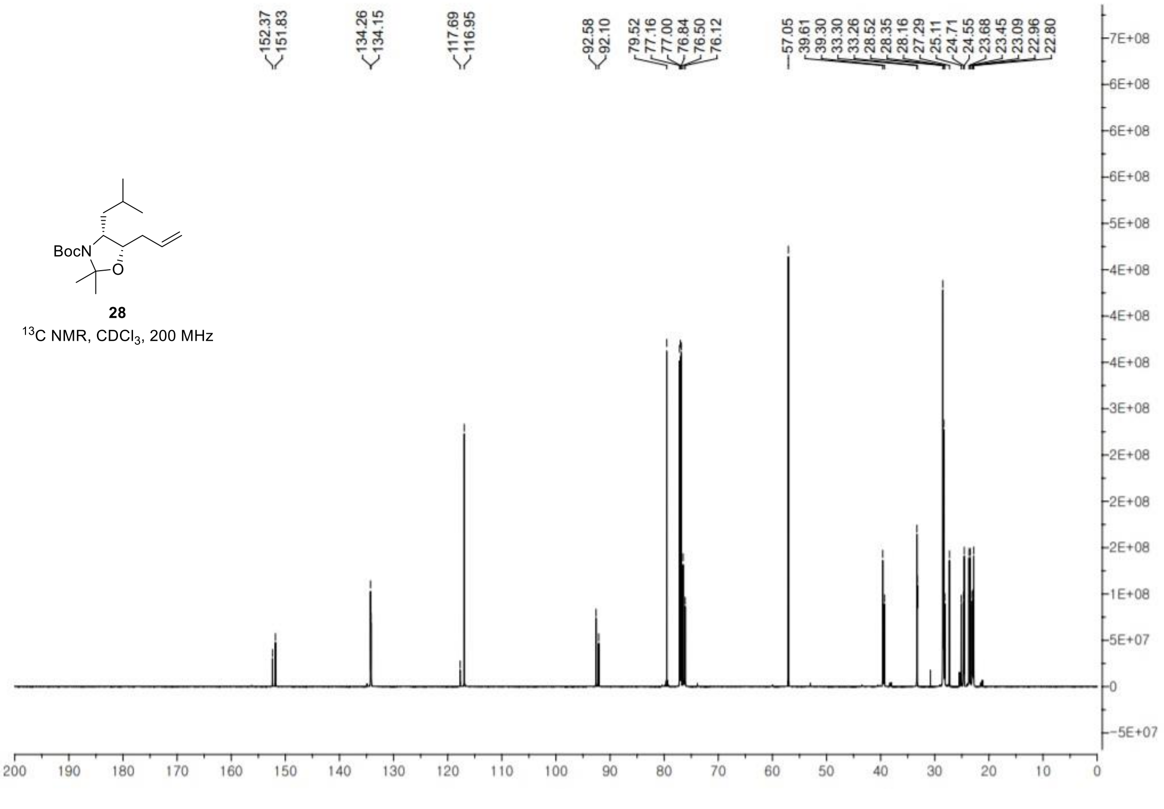

^{1}H NMR and ^{13}C NMR spectra of **29**

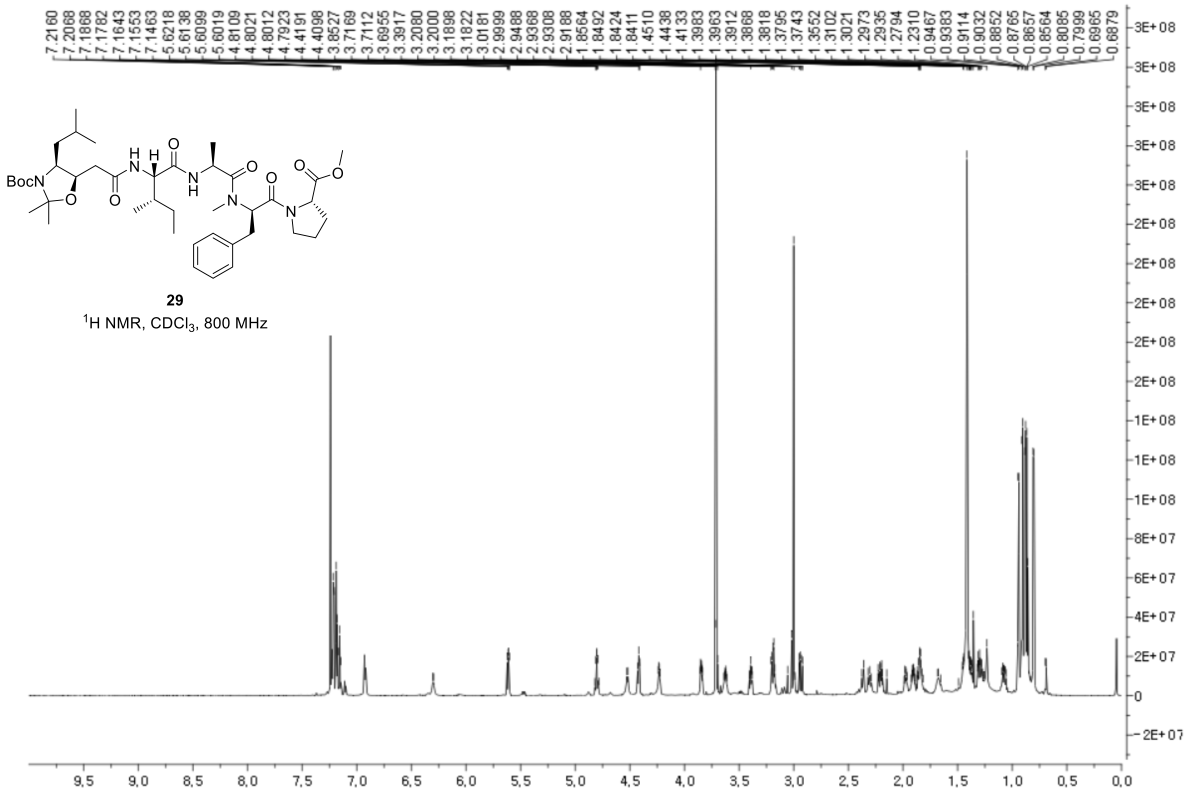

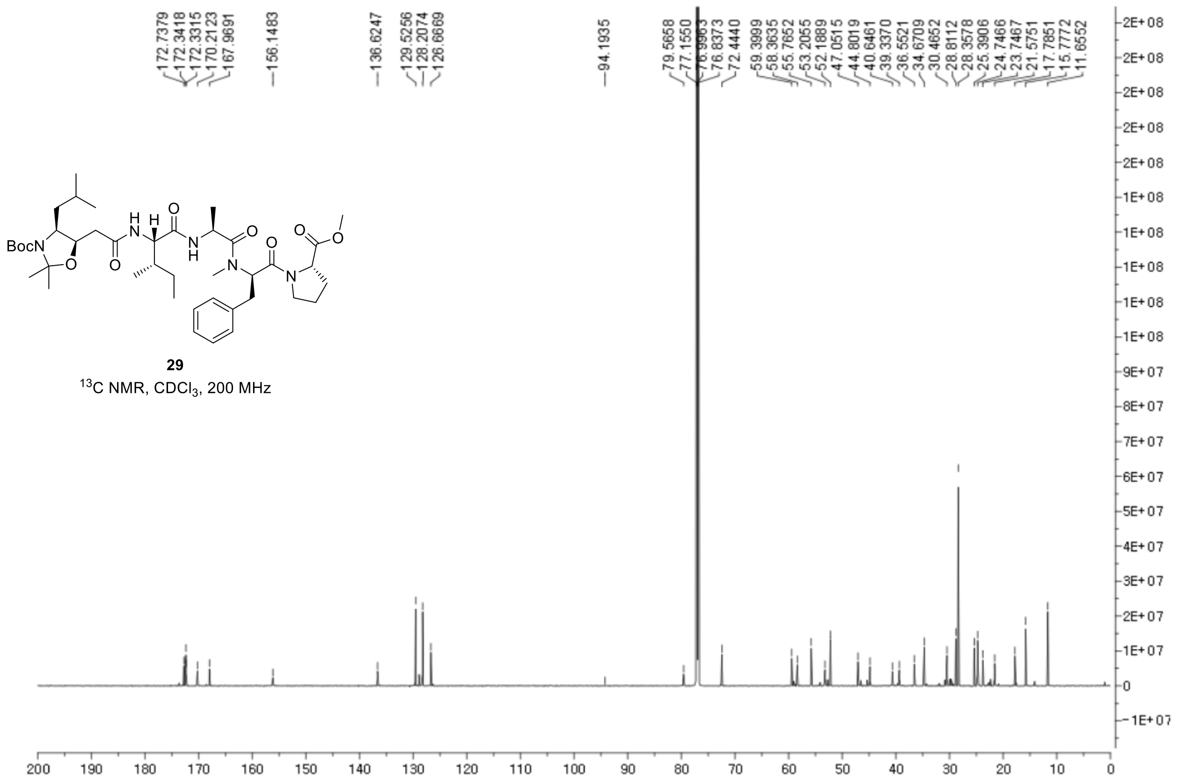

^{1}H NMR and ^{13}C NMR spectra of **30**

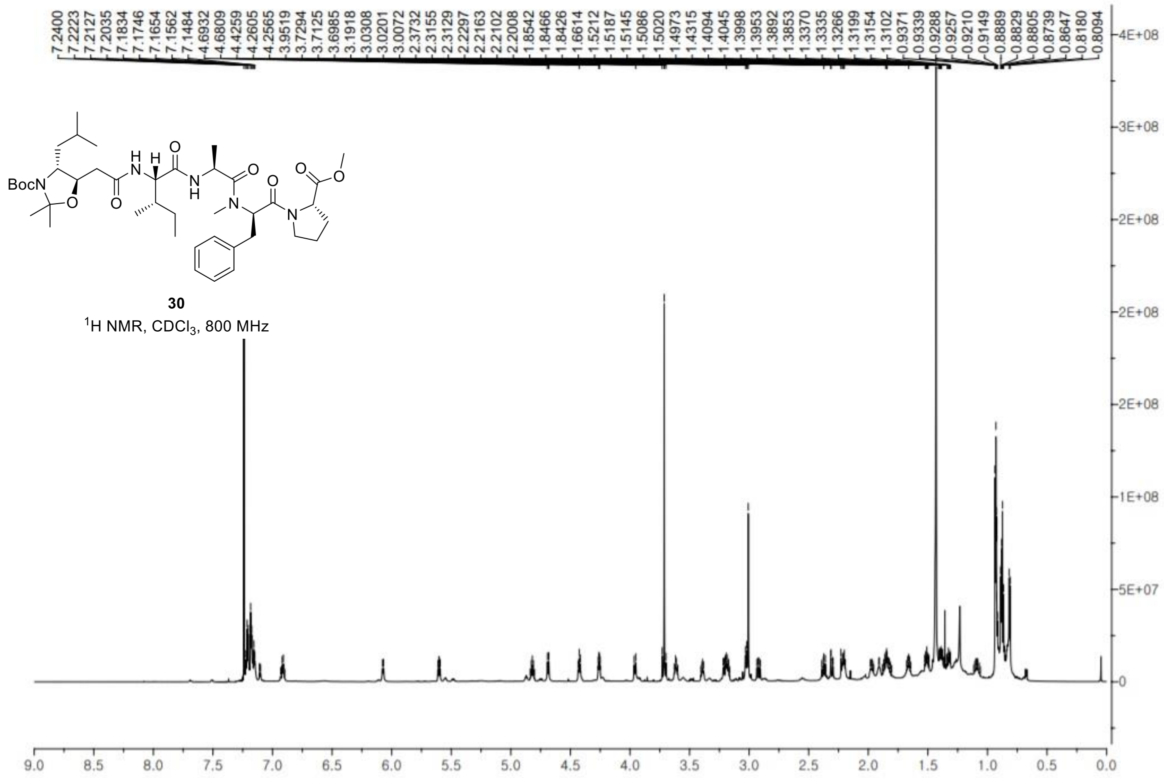

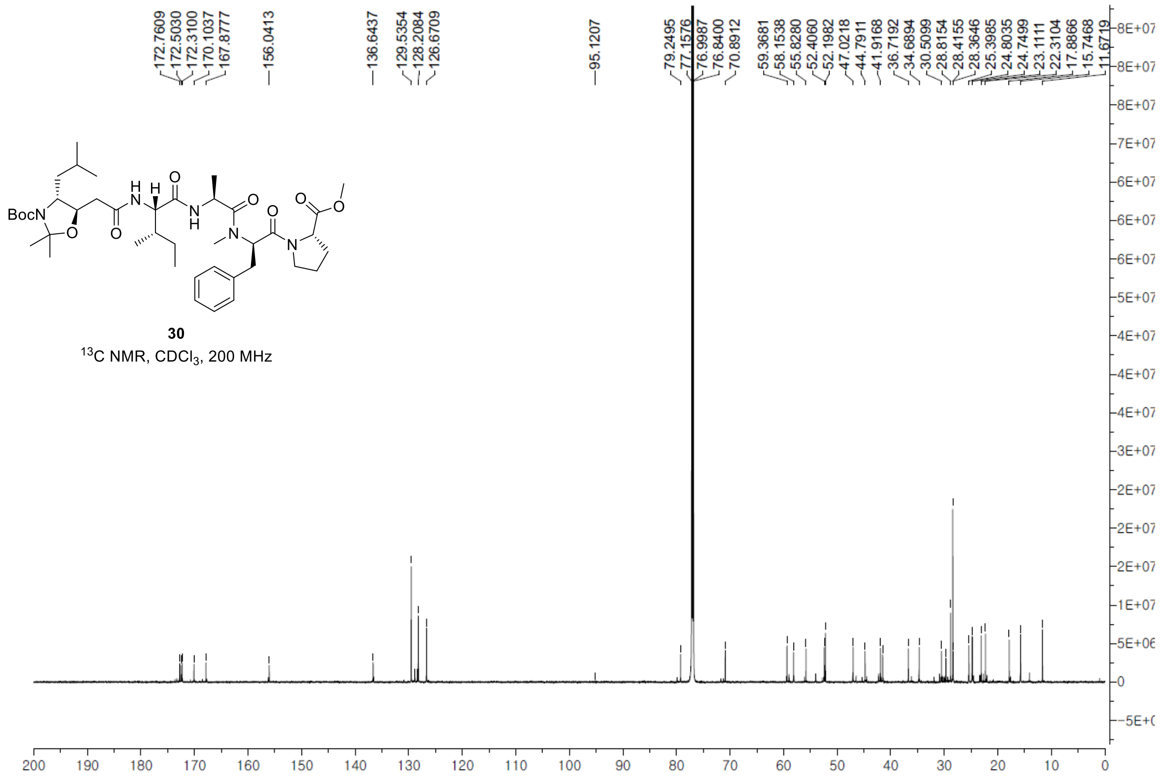

^{1}H NMR and ^{13}C NMR spectra of **31**

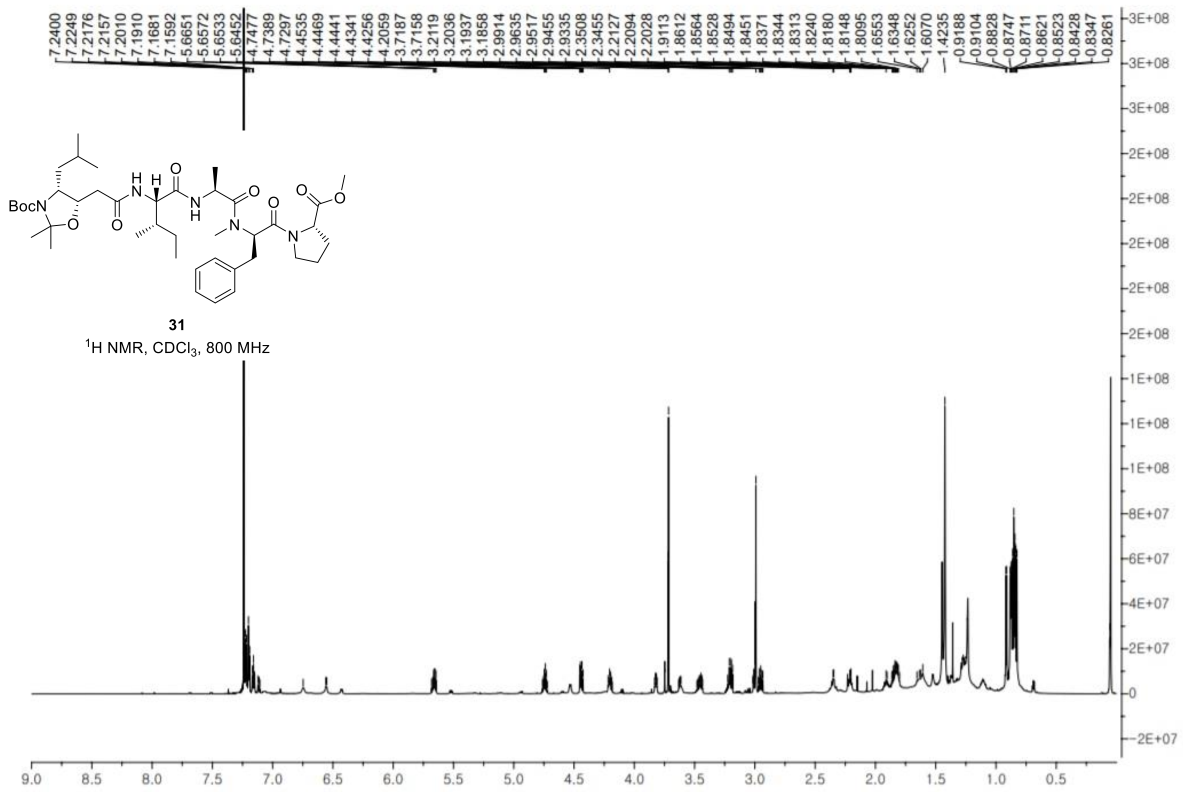

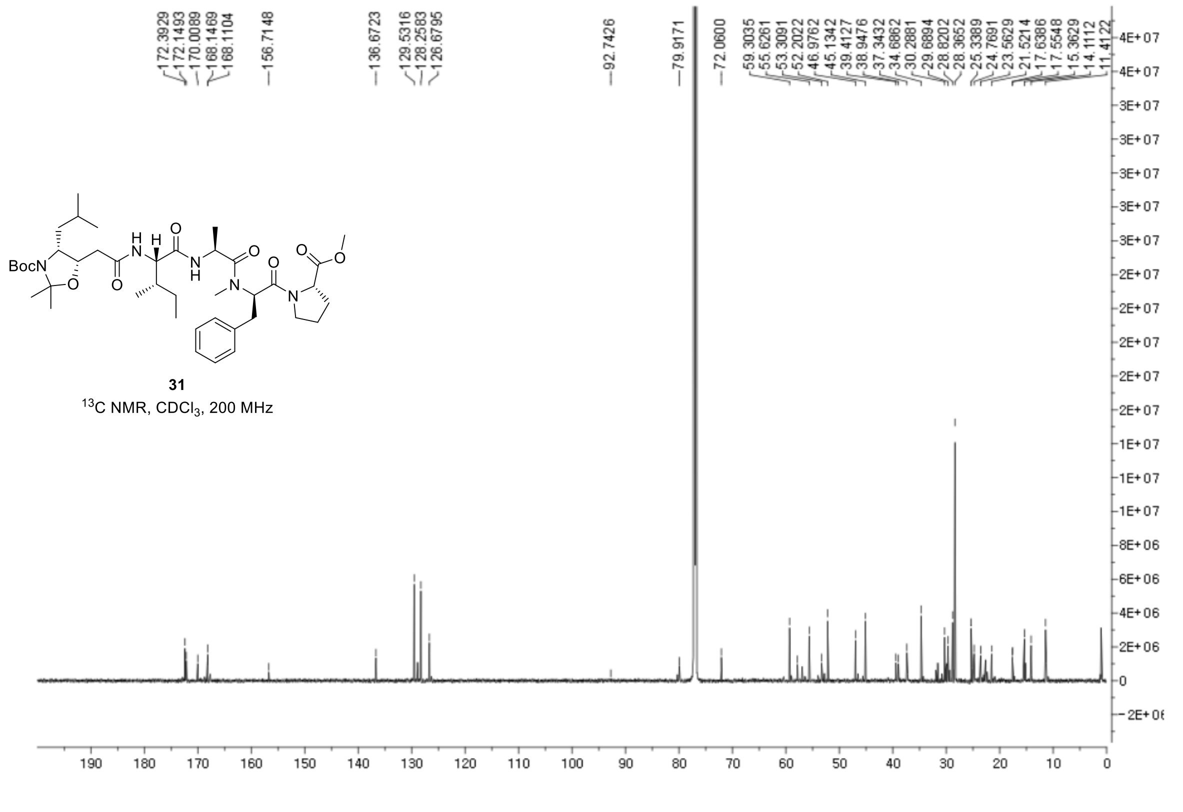

^{1}H NMR and ^{13}C NMR spectra of izenamide B variant (**4**)

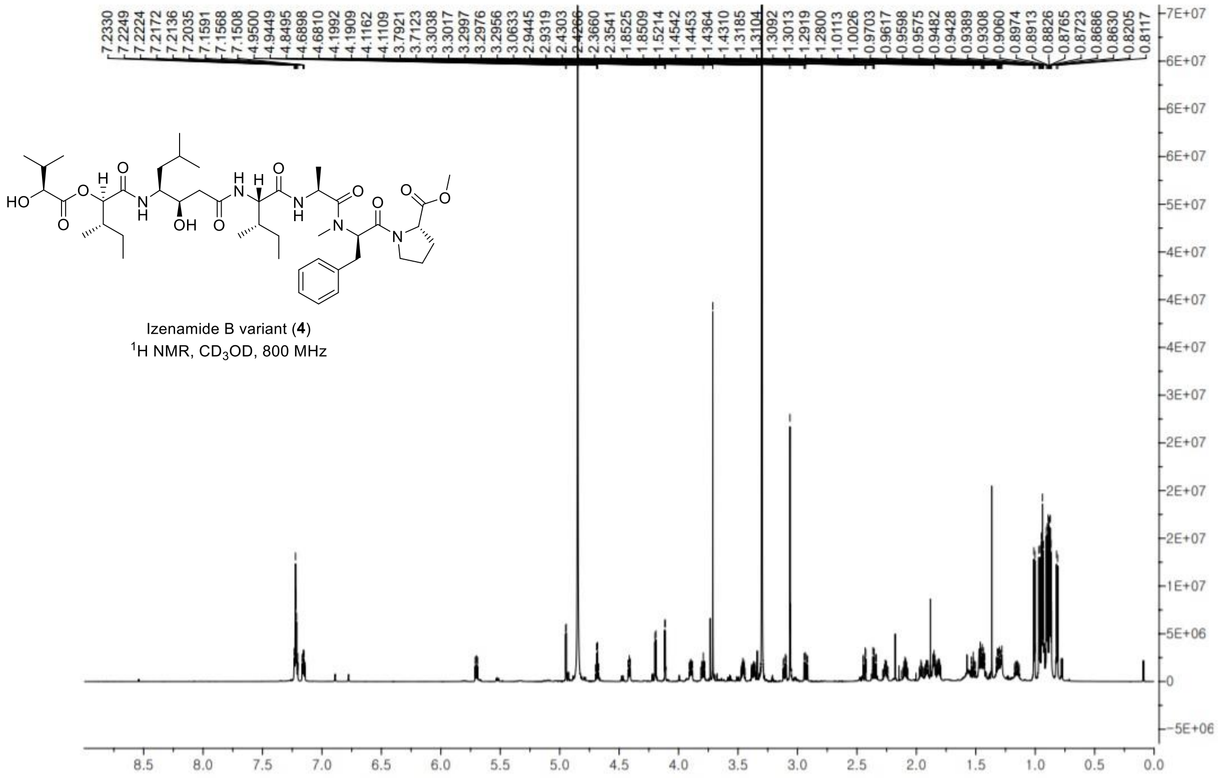

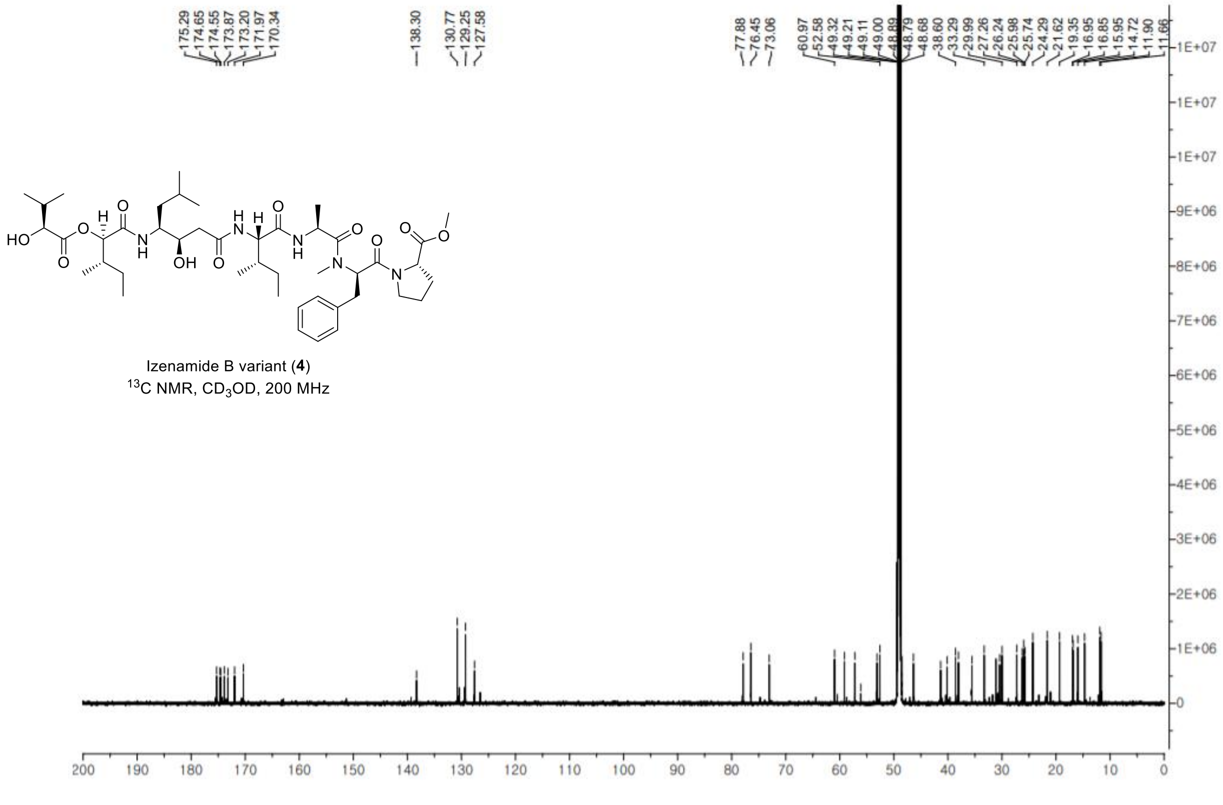

^{1}H NMR and ^{13}C NMR spectra of izenamide B variant (**5**)

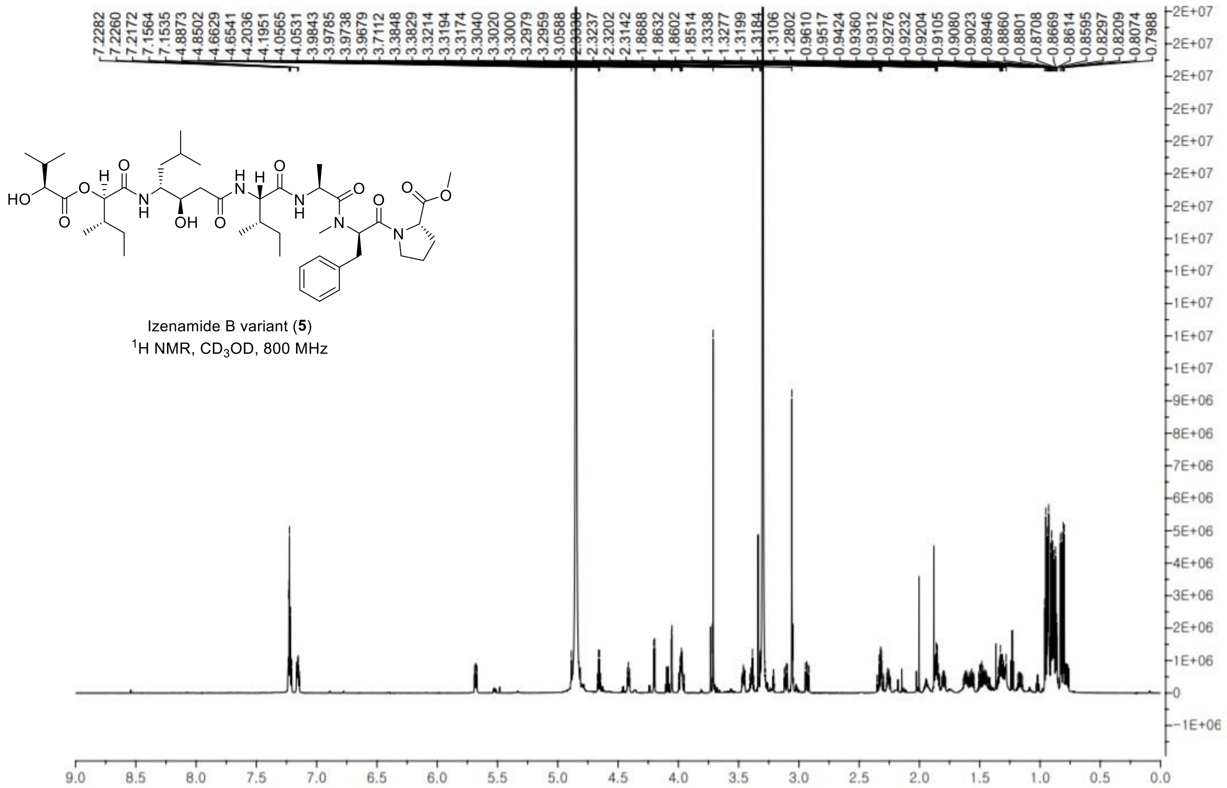

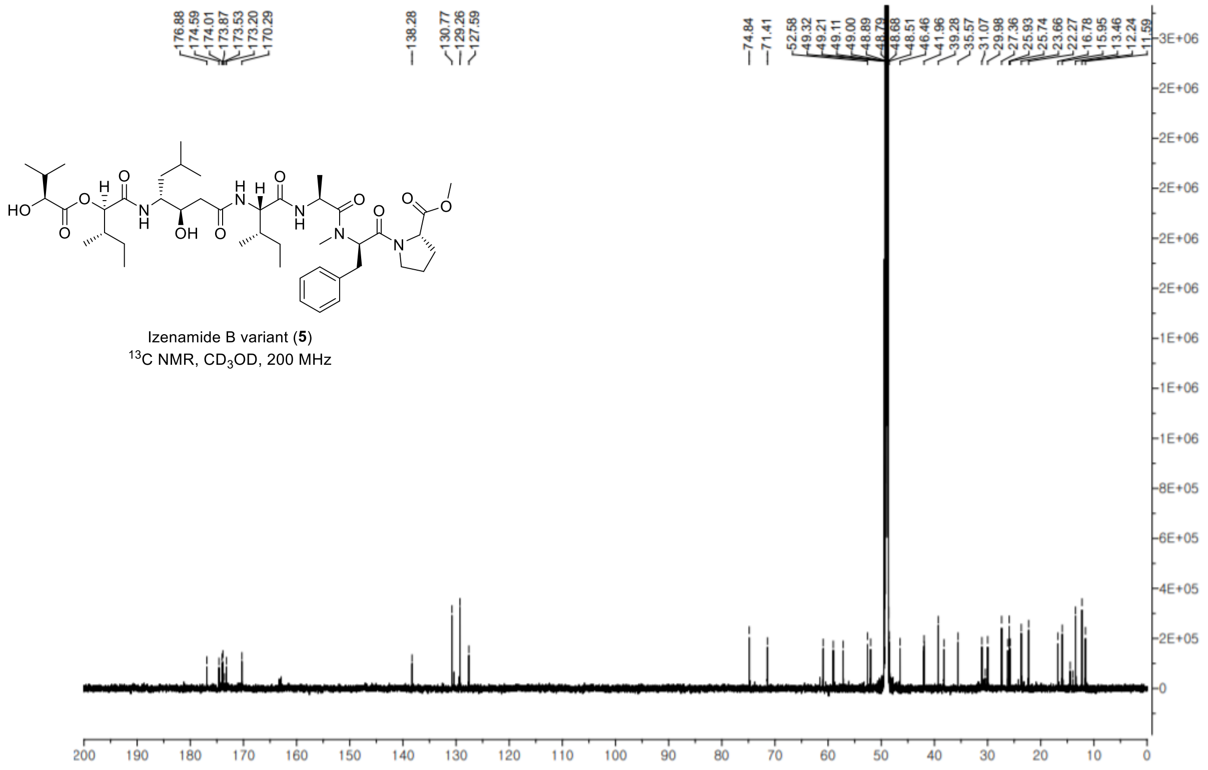

^{1}H NMR and ^{13}C NMR spectra of izenamide B variant (**6**)

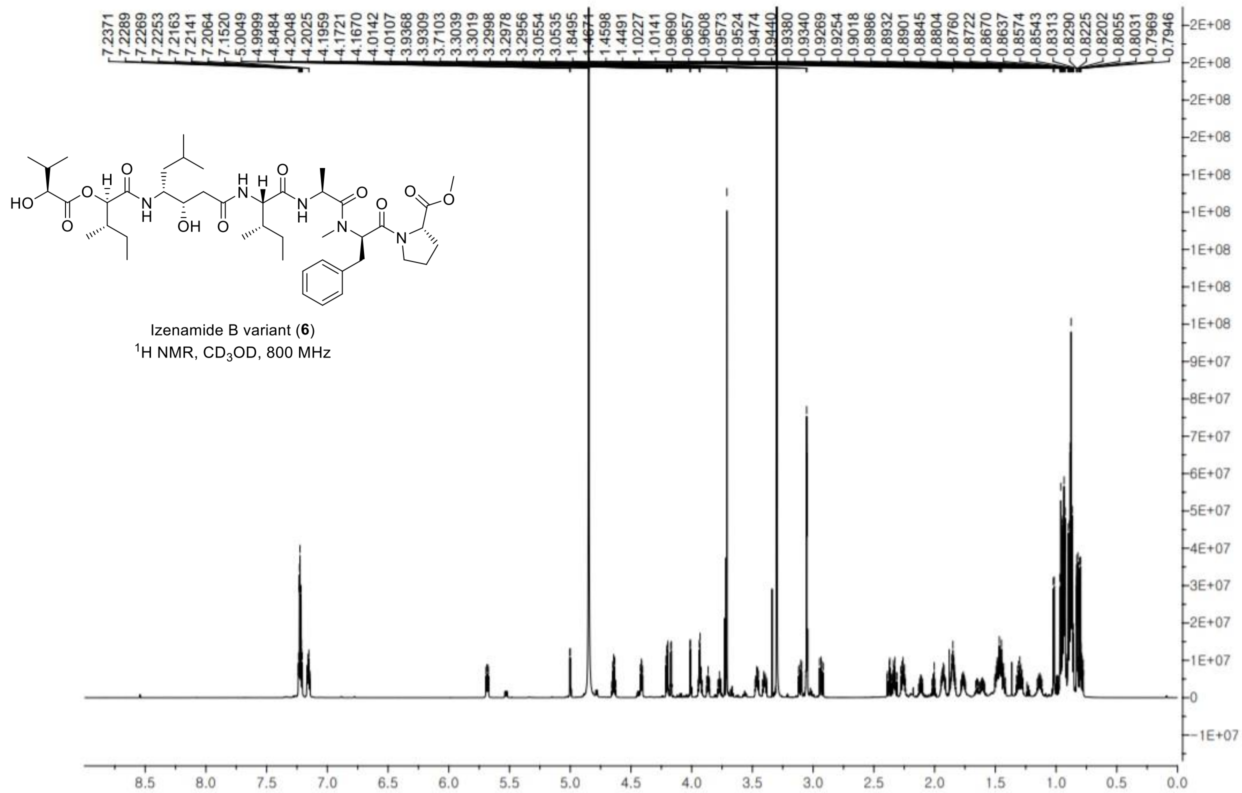

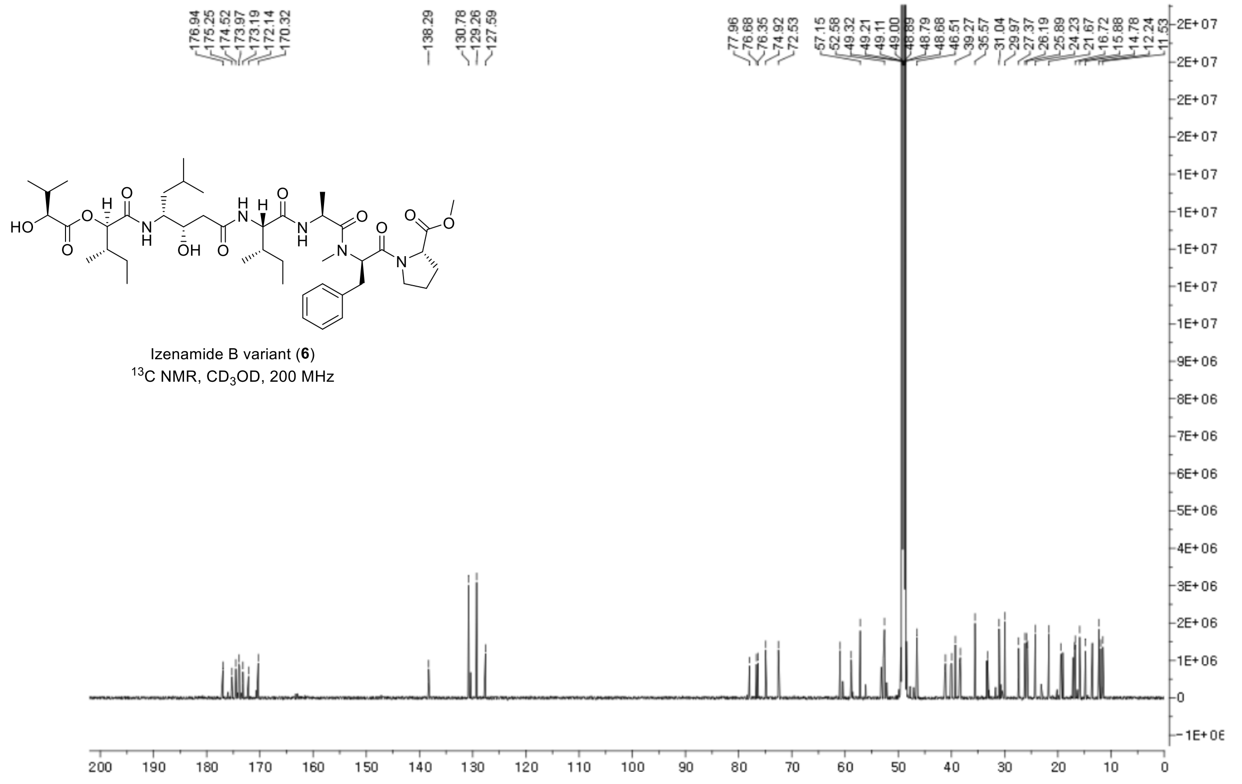

^{1}H NMR and ^{13}C NMR spectra of **20**

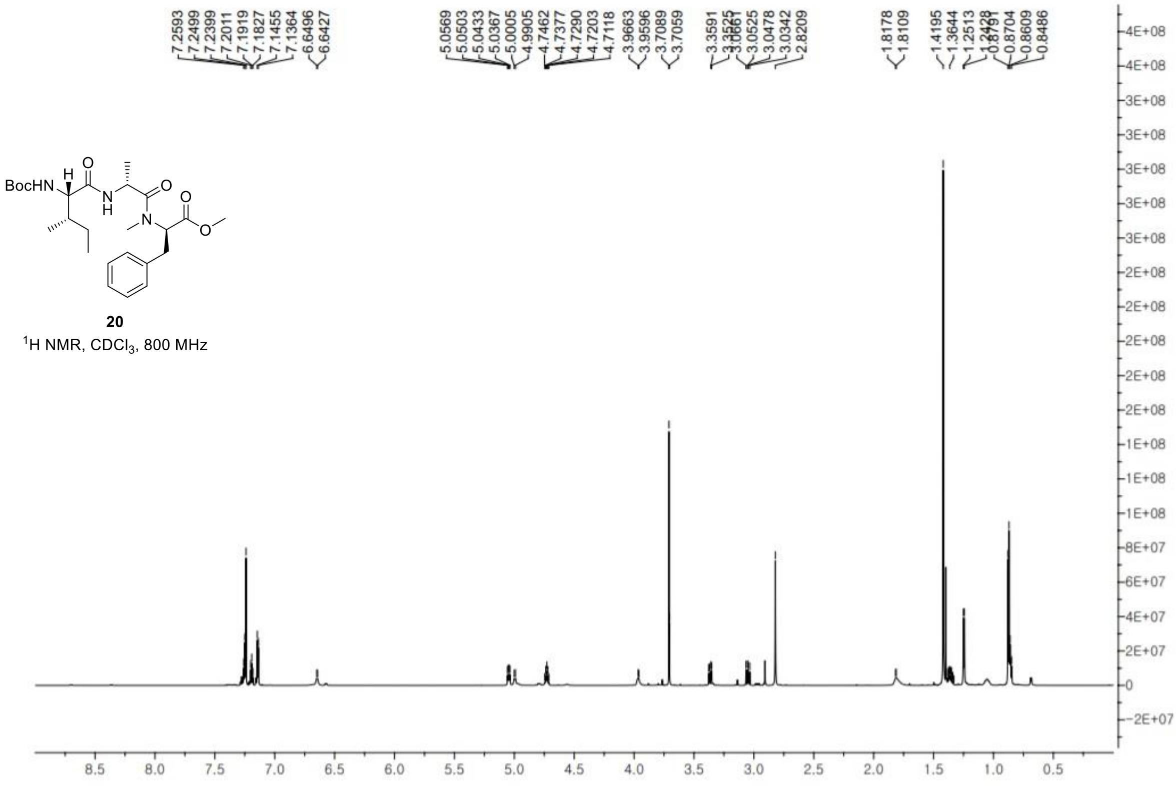

20

^{1}H NMR, $CDCl_3$, 800 MHz

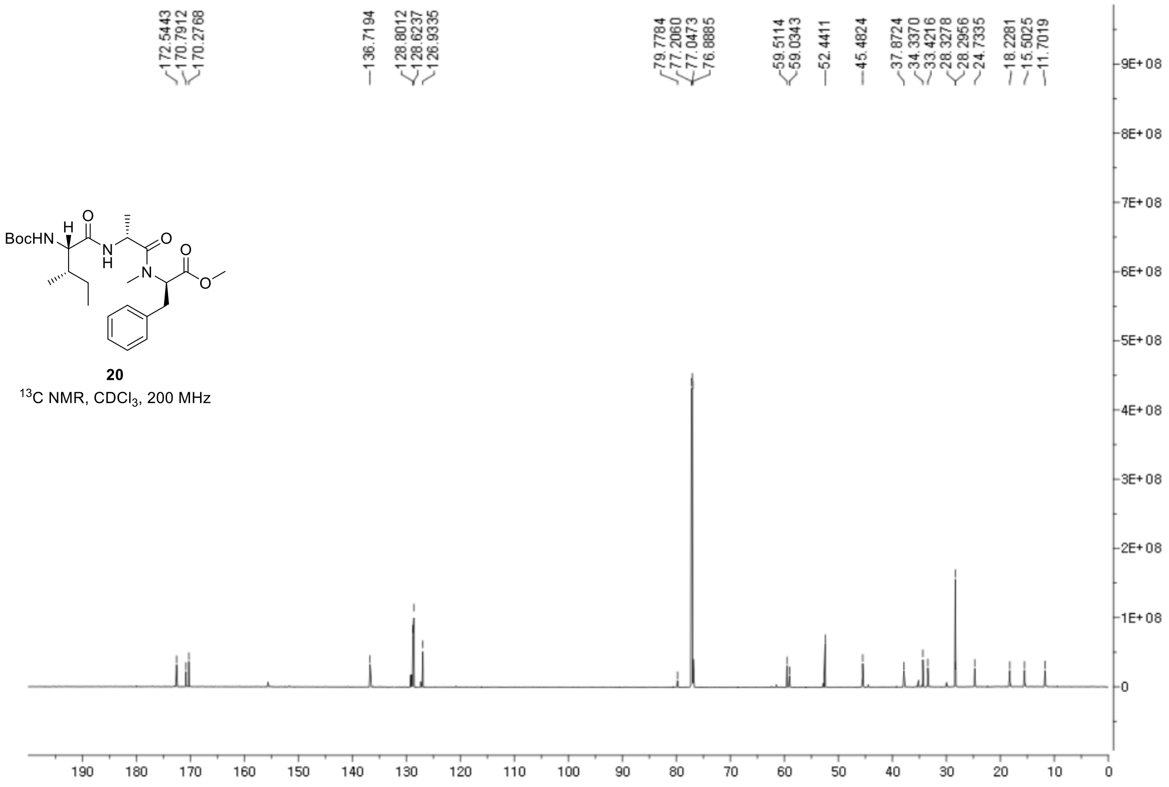

20

^{13}C NMR, $CDCl_3$, 200 MHz

^{1}H NMR and ^{13}C NMR spectra of **15**

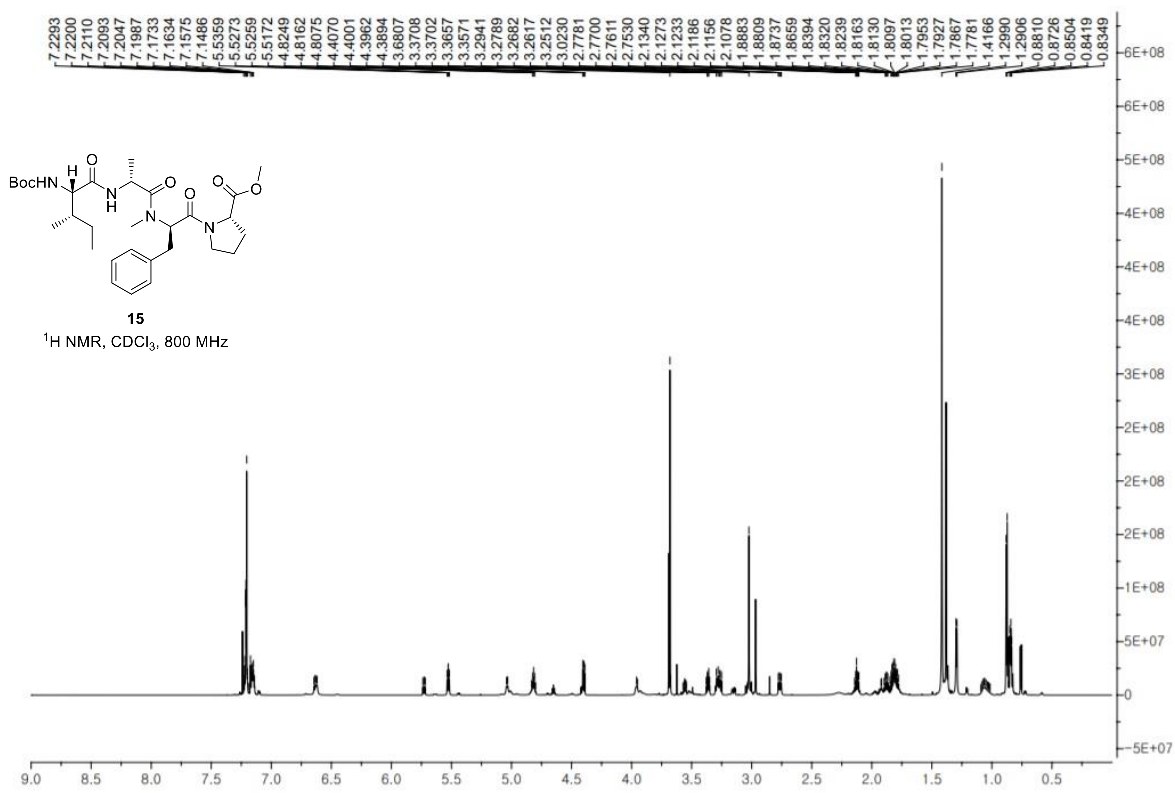

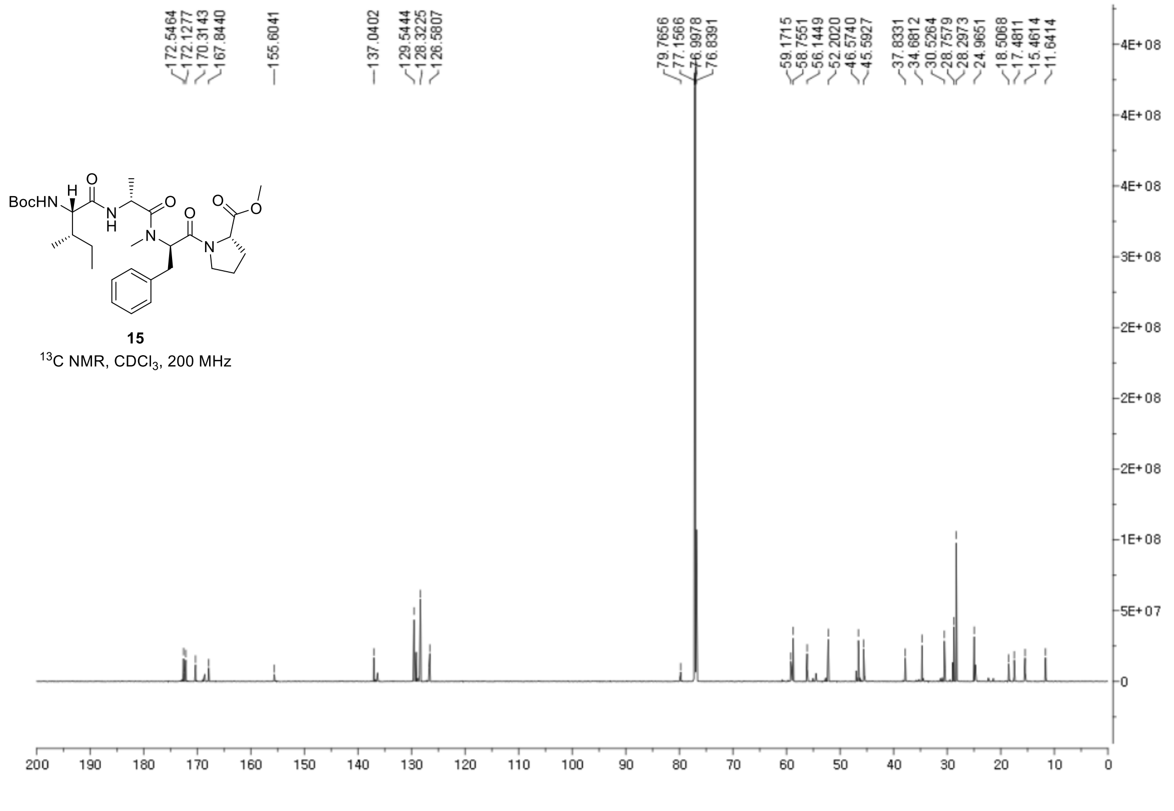

^{1}H NMR and ^{13}C NMR spectra of **33**

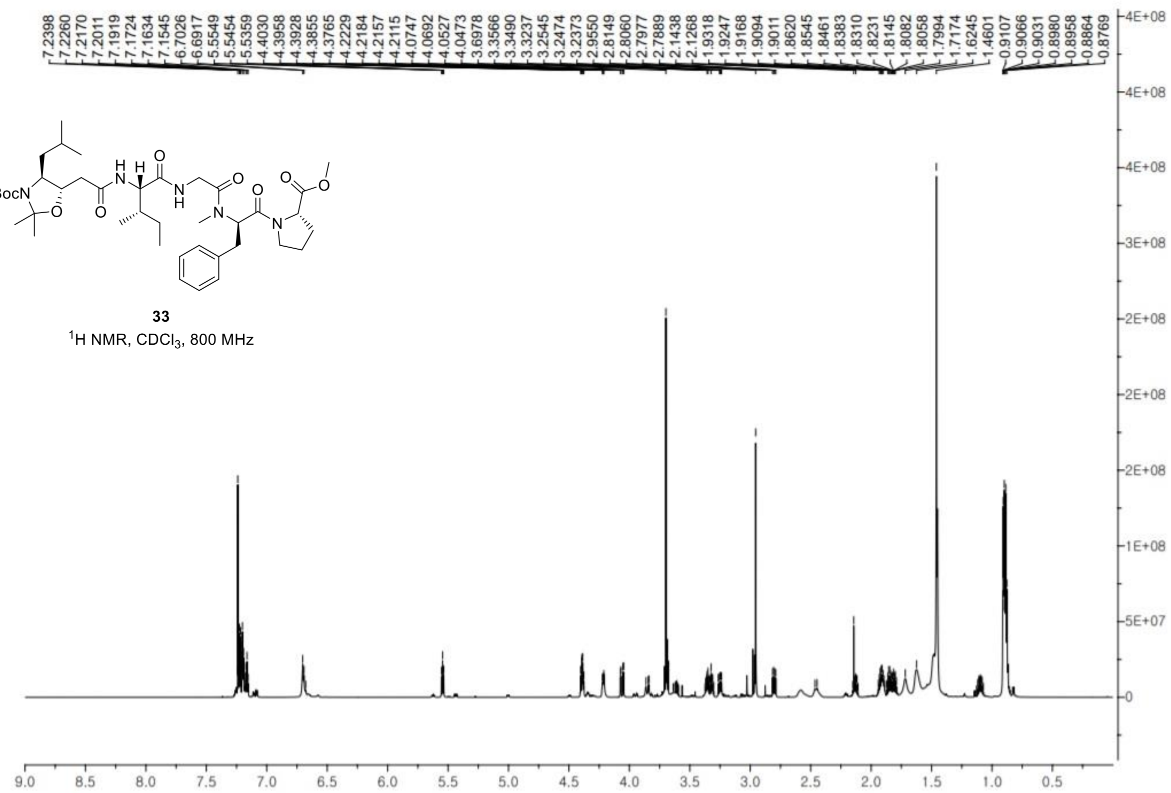

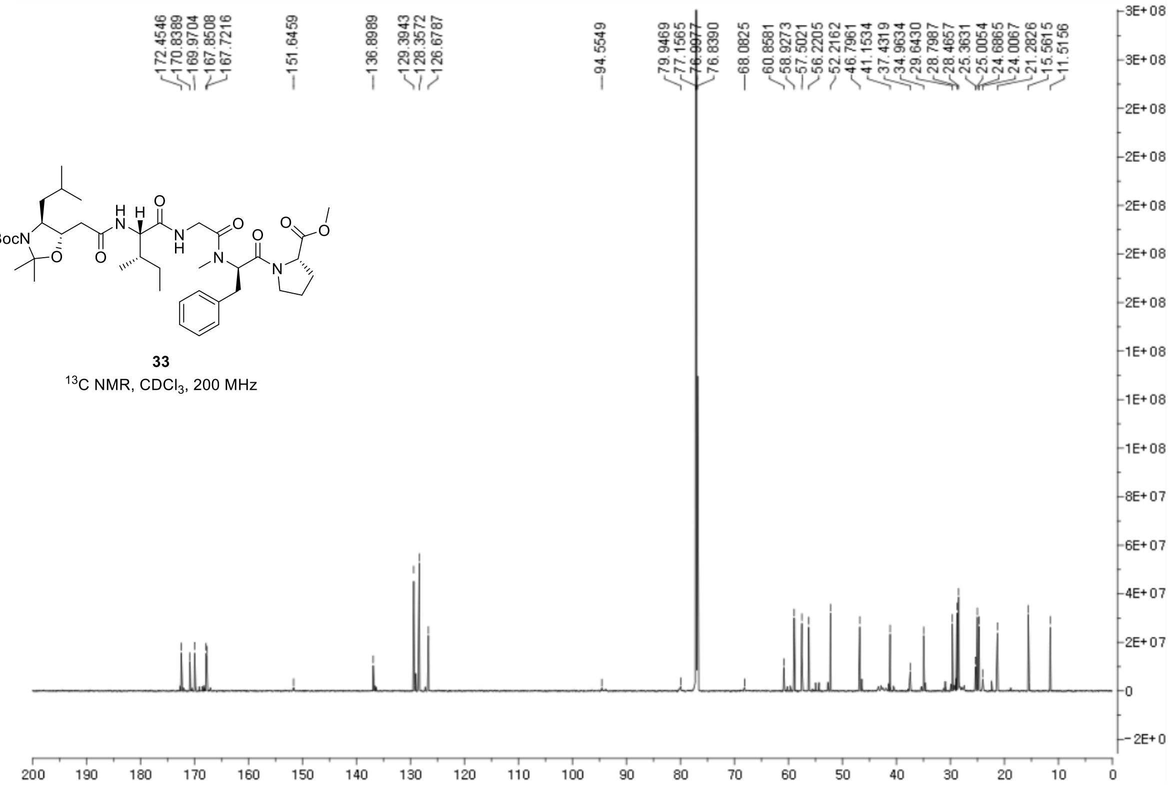

^{1}H NMR and ^{13}C NMR spectra of **34**

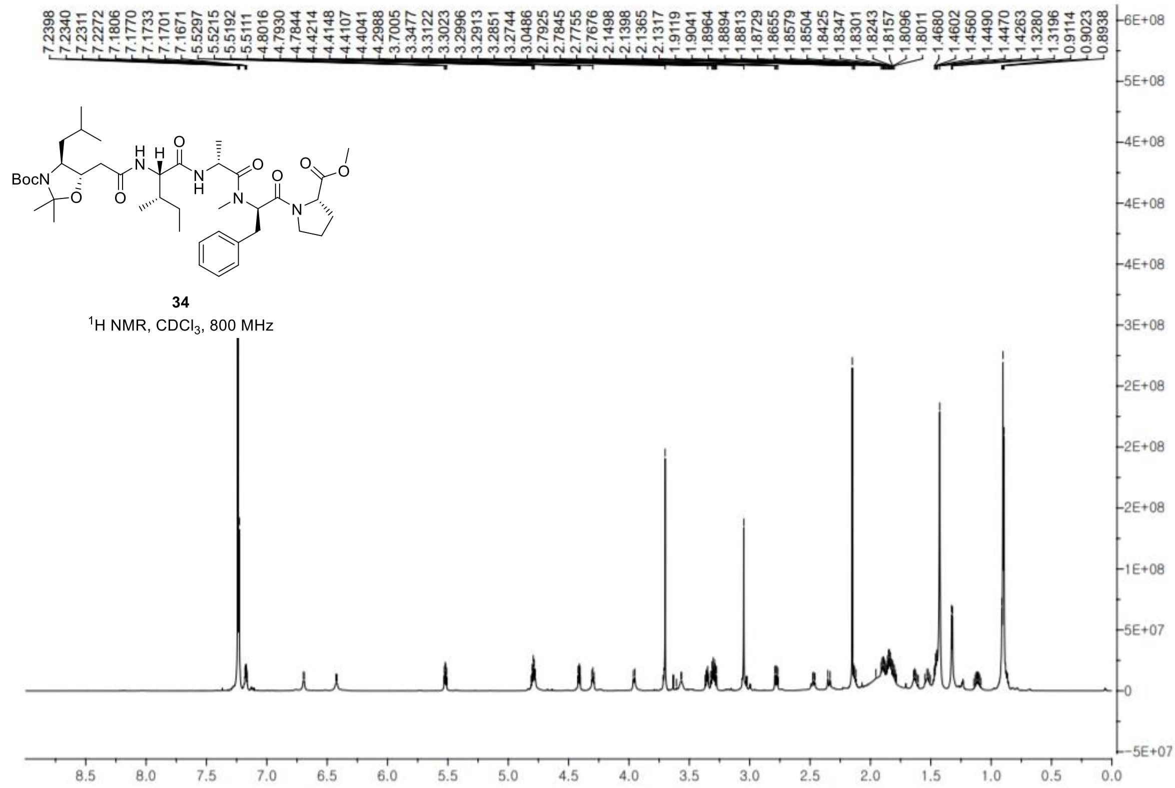

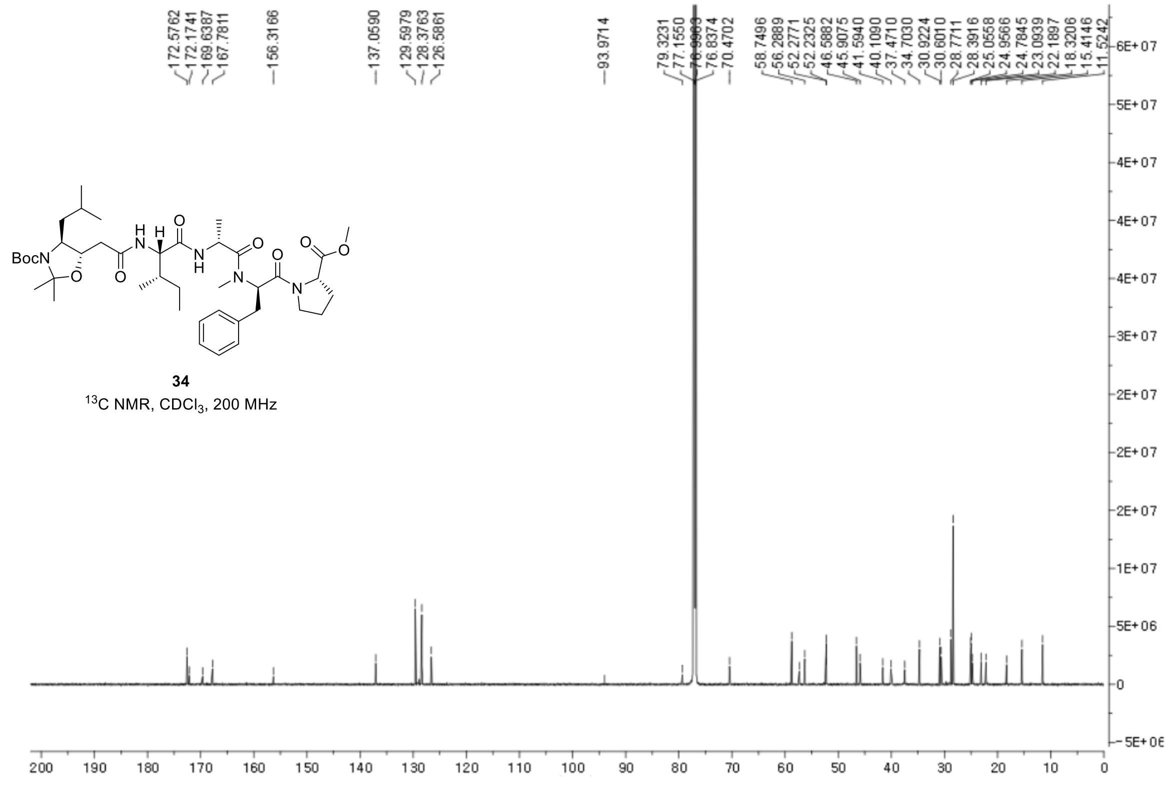

^{1}H NMR and ^{13}C NMR spectra of the Sta-incoporated izenamide C (**7**)

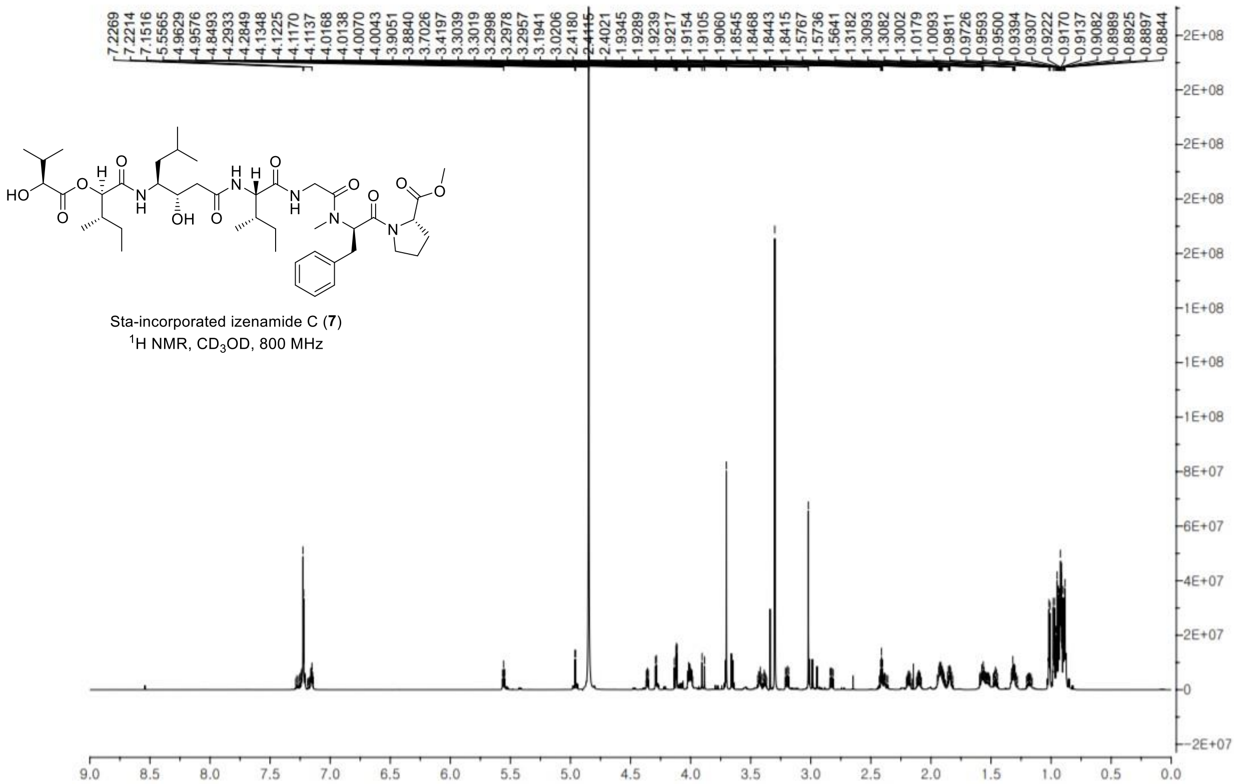

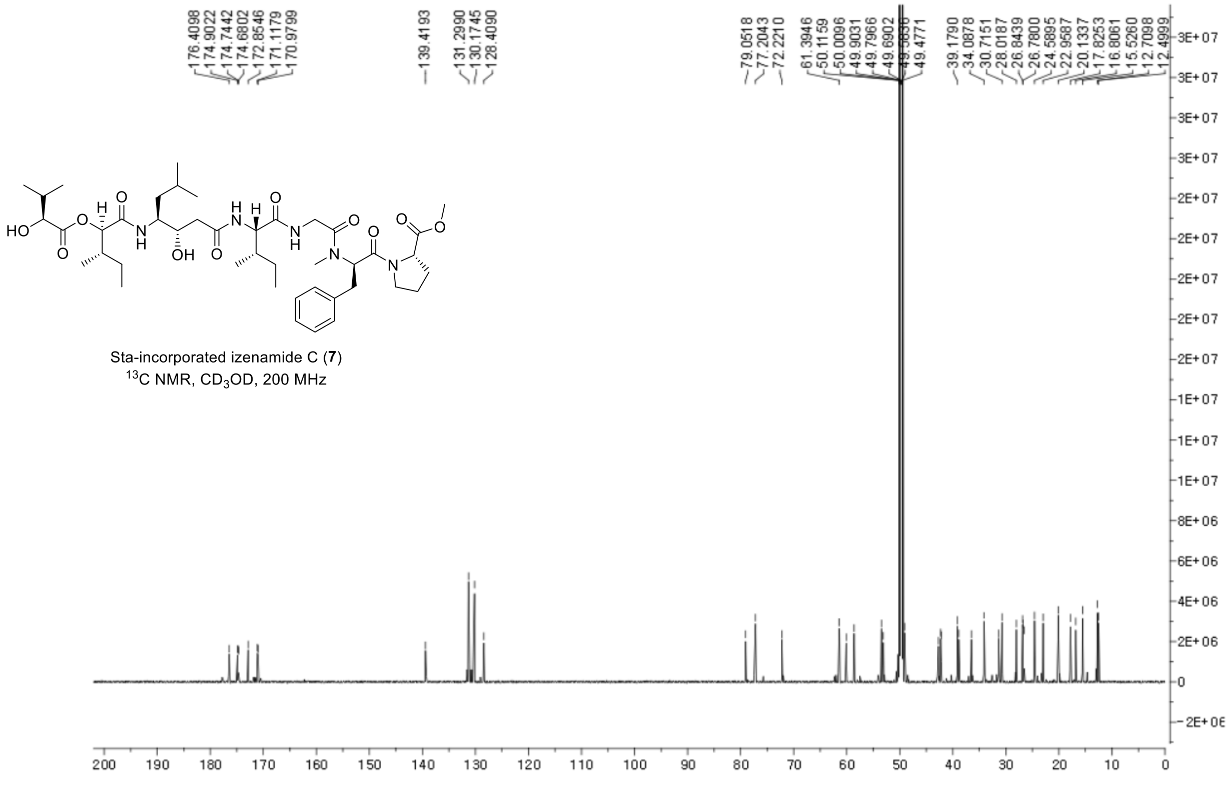

^{1}H NMR and ^{13}C NMR spectra of 18-*epi*-izenamide B (**8**)

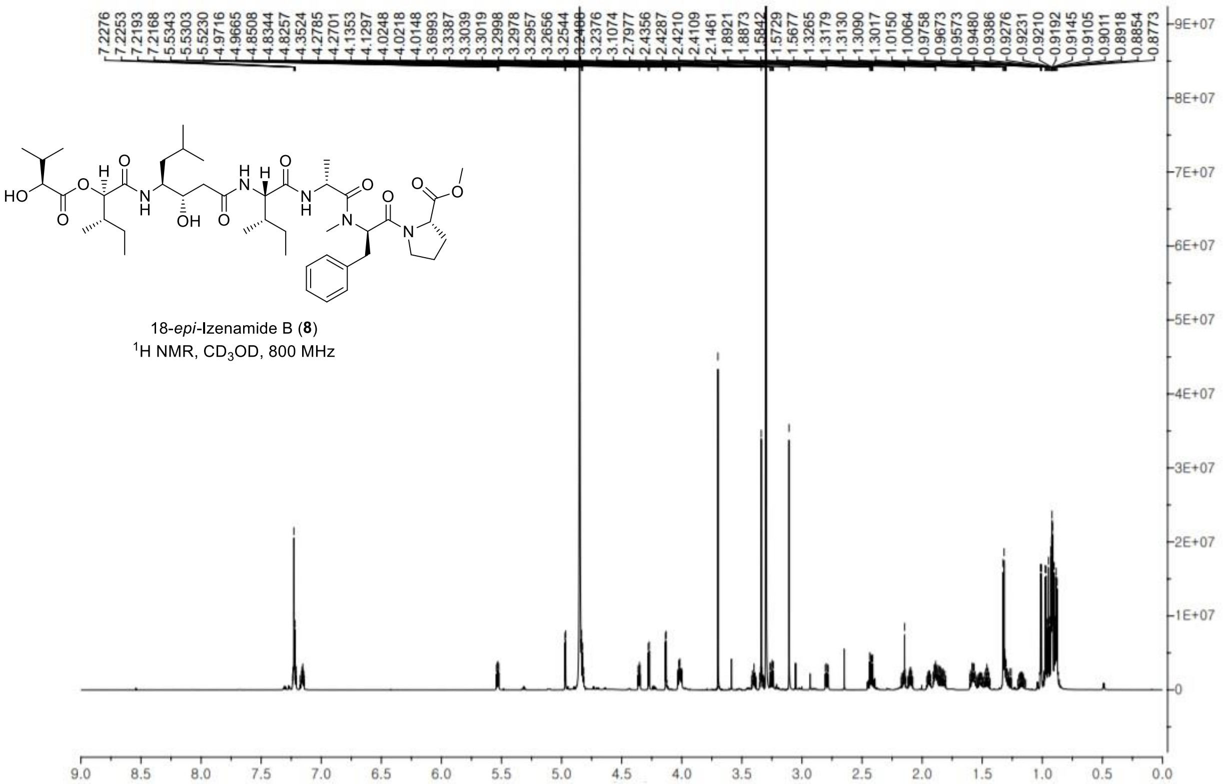

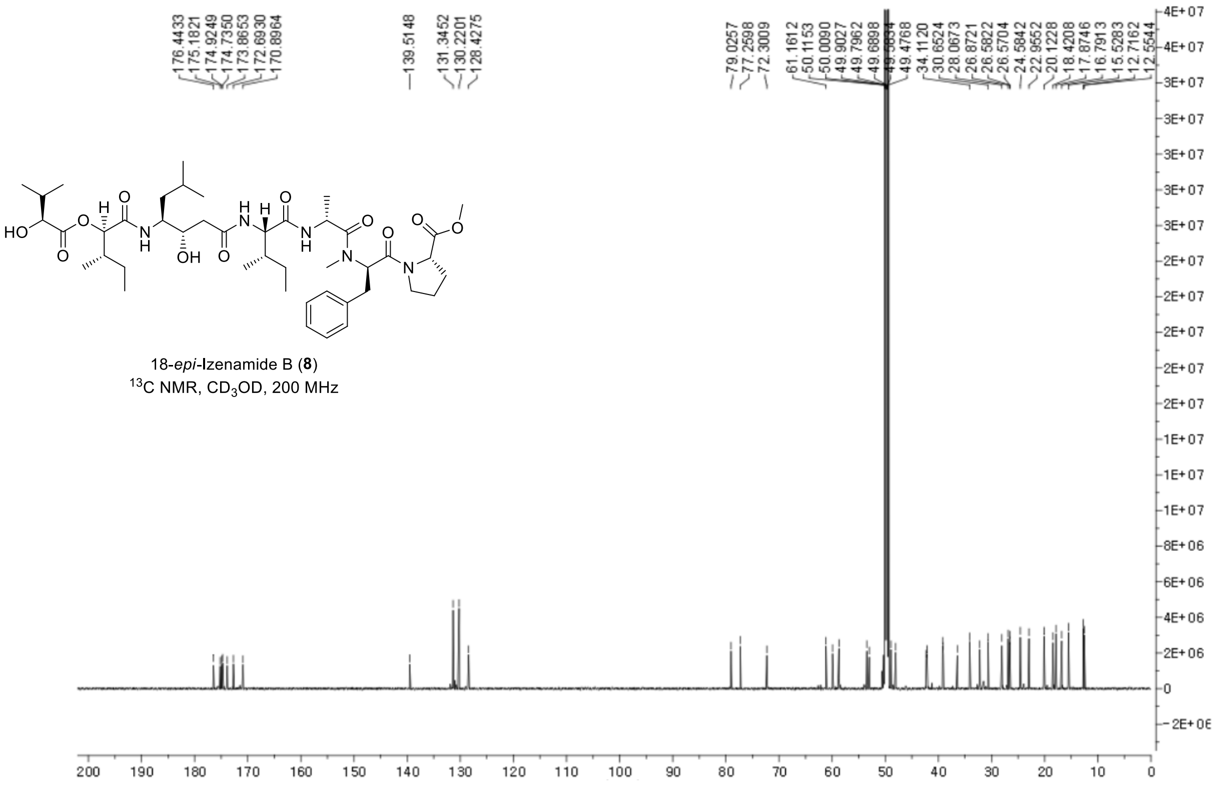

II. Comparison of ^{1}H, ^{13}C NMR spectra of acetonide 24, 27, and 28

^{1}H NMR of (**24, 27**, and **28**), $CDCl_3$, 800 MHz; δ 7.00-0.60

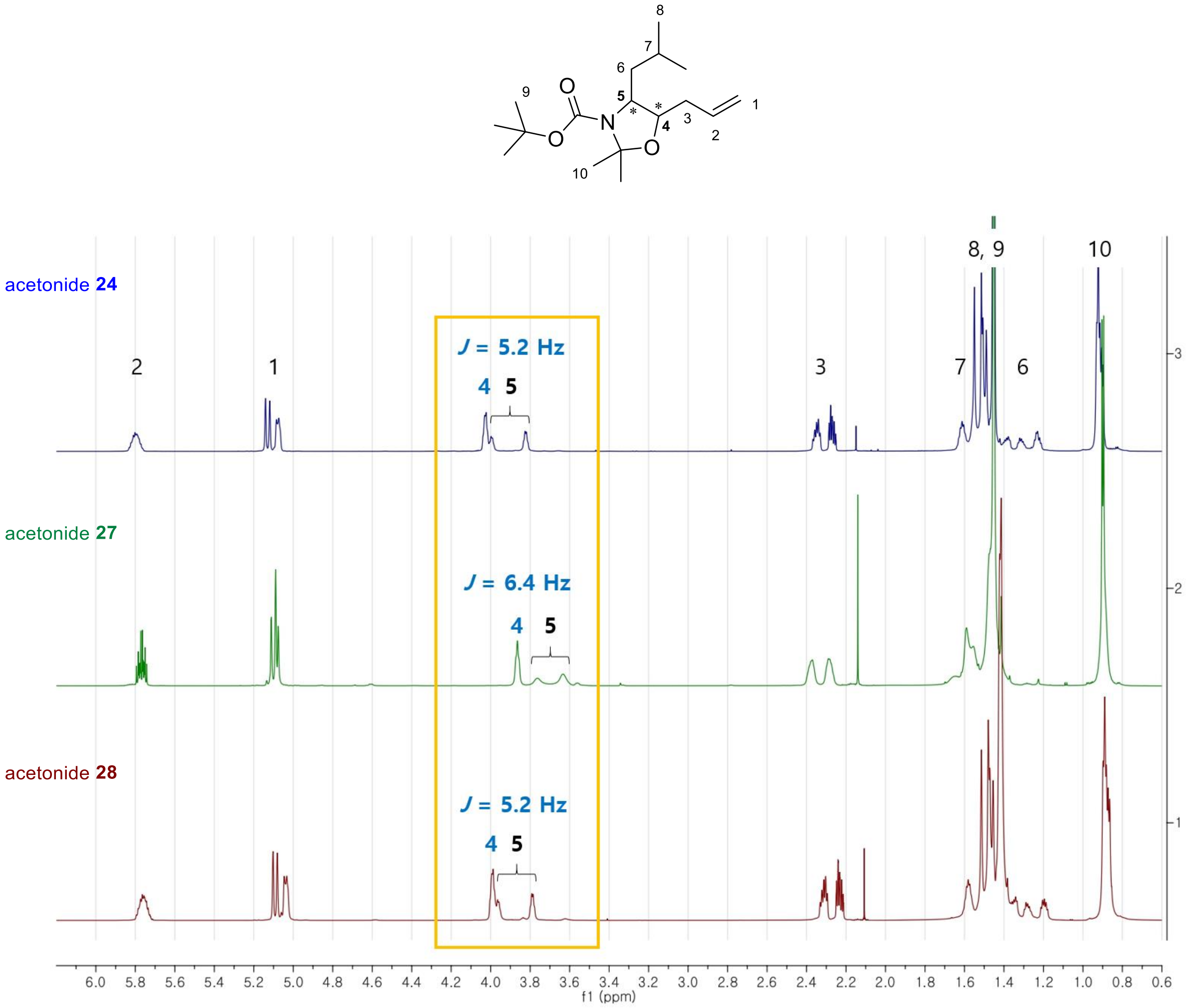

	24	27	28
C1	5.14 (s, 0.5H), 5.12 (s, 0.5H), 5.08 (d, *J* = 9.4 Hz, 1H)	5.13 (s, 0.5H), 5.09 (s, 0,5H), 5.08 (d, *J* = 10.4 Hz, 1H)	5.10 (s, 0.5H), 5.08 (s, 0.5H), 5.04 (d, *J* = 9.8 Hz, 1H)
C2	5.80-5.76 (m, 1H)	5.77 (ddt, *J* = 17.2, 10.2, 7.0 Hz, 1H)	5.80-5.72 (m, 1H)
C3	2.35 (dt, *J* = 14.1, 6.9 Hz, 1H), 2.27 (dt, *J* = 13.2, 6.4 Hz, 1H)	2.40-2.35 (m, 1H), 2.30-2.27 (m, 1H)	2.31 (dt, *J* = 14.3, 7.0 Hz, 1H), 2.25-2.21 (m, 1H),
C4	4.03 (d, *J* = 5.2 Hz, 1H)	3.86 (t, *J* = 6.4 Hz, 1H)	3.99 (d, *J* = 5.2 Hz, 1H)
C5	3.99 (d, *J* = 5.0 Hz, 0.5H), 3.82 (d, *J* = 5.1 Hz, 0.5H)	3.76 (s, 0.5H), 3.63 (s, 0.5H)	3.96 (d, *J* = 5.1 Hz, 0.5H), 3.79 (d, *J* = 5.2 Hz, 0.5H)
C6	1.43-1.36 (m, 1H), 1.33-1.21 (m, 1H)	1.50-1.42 (m, 2H)	1.38-1.31 (m, 1H), 1.30-1.18 (m, 1H)
C7	1.65-1.60 (m, 1H)	1.70-1.61 (m, 1H)	1.60-1.56 (m, 1H)
C8	1.53 (d, *J* = 28.5 Hz, 3H), 1.50 (d, *J* = 13.9 Hz, 3H)	1.60-1.53 (m, 3H) 1.50-1.42 (m, 3H)	1.50 (d, *J* = 28.0 Hz, 3H), 1.46 (d, *J* = 13.2 Hz, 3H)
C9	1.45 (s, 9H)	1.50-1.42 (m, 9H)	1.41 (s, 9H)
C10	0.94-0.89 (m, 6H)	0.90 (d, *J* = 6.4 Hz, 6H)	0.91-0.85 (m, 6H)

^{13}C NMR of (**24, 27**, and **28**), $CDCl_3$, 200 MHz; δ 200.00-10.0

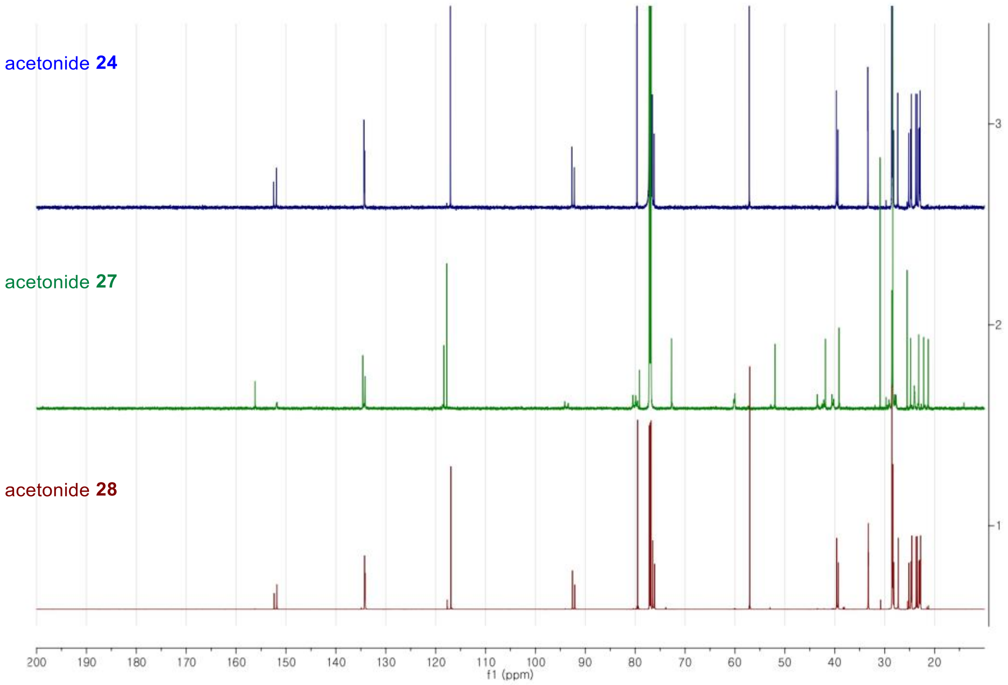

III. Comparison of NMR spectra of Izenamide B Variants (4-6, 8) and Izenamide B (2)

^{1}H NMR of (**4-6**, **8**), CD_3OD, 800 MHz; δ 6.00-2.70 and 2.60-0.60

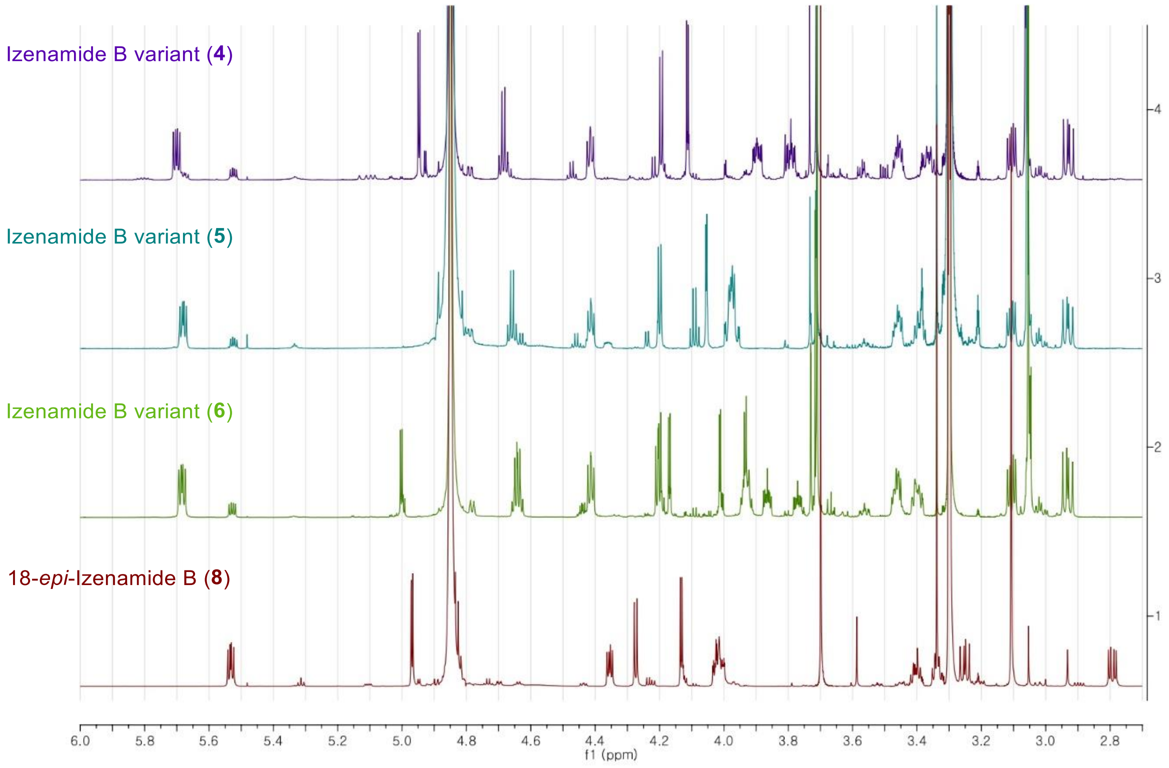

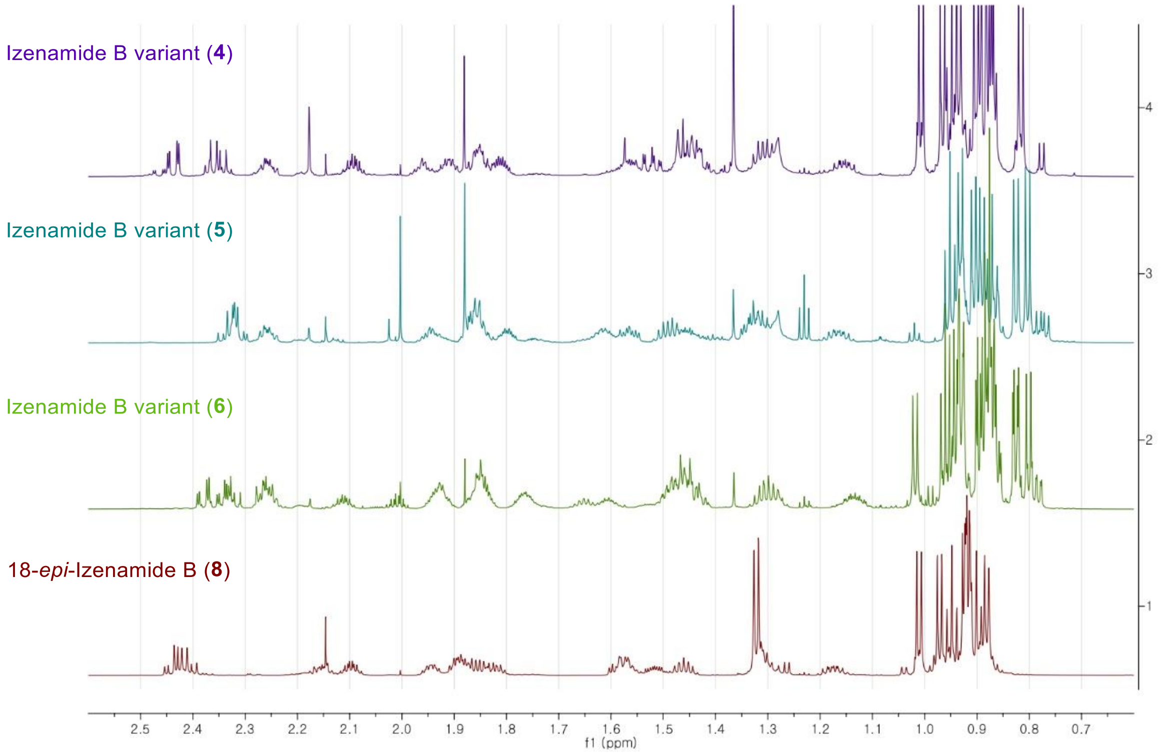

^{13}C NMR of (**4**-**6**, **8**), CD_3OD, 200 MHz; δ 190.0-120.0 and 90.0-5.0

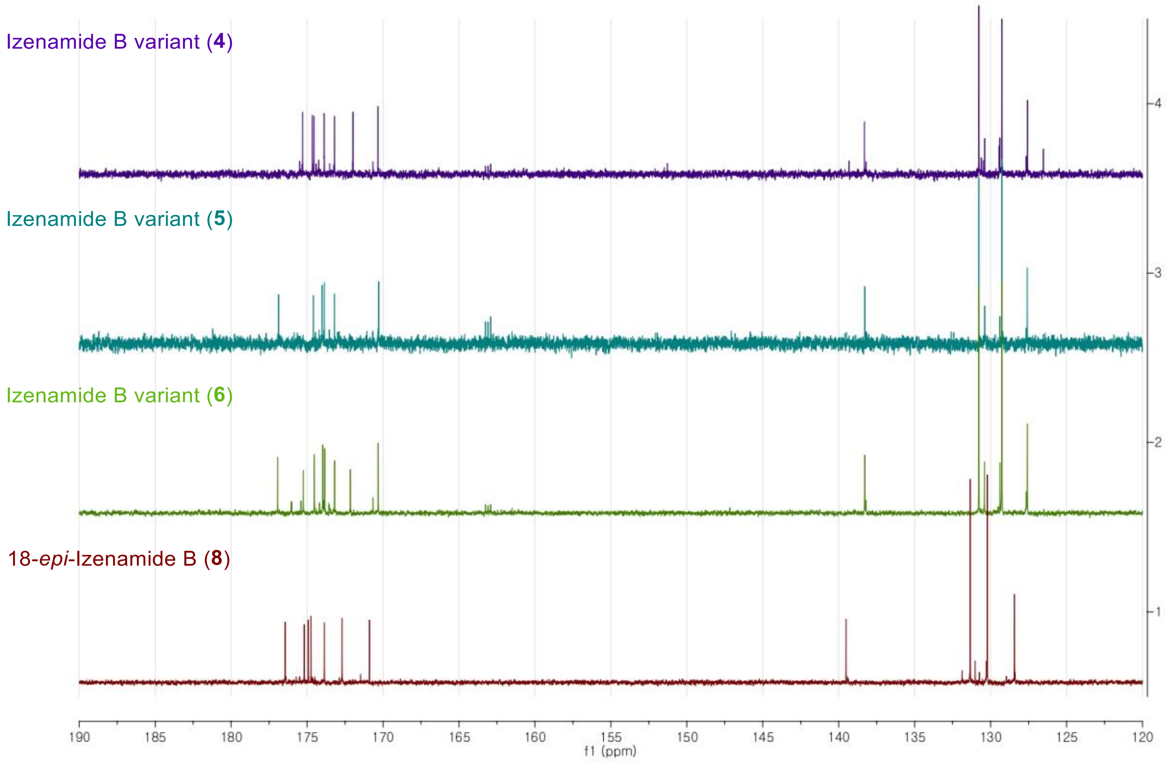

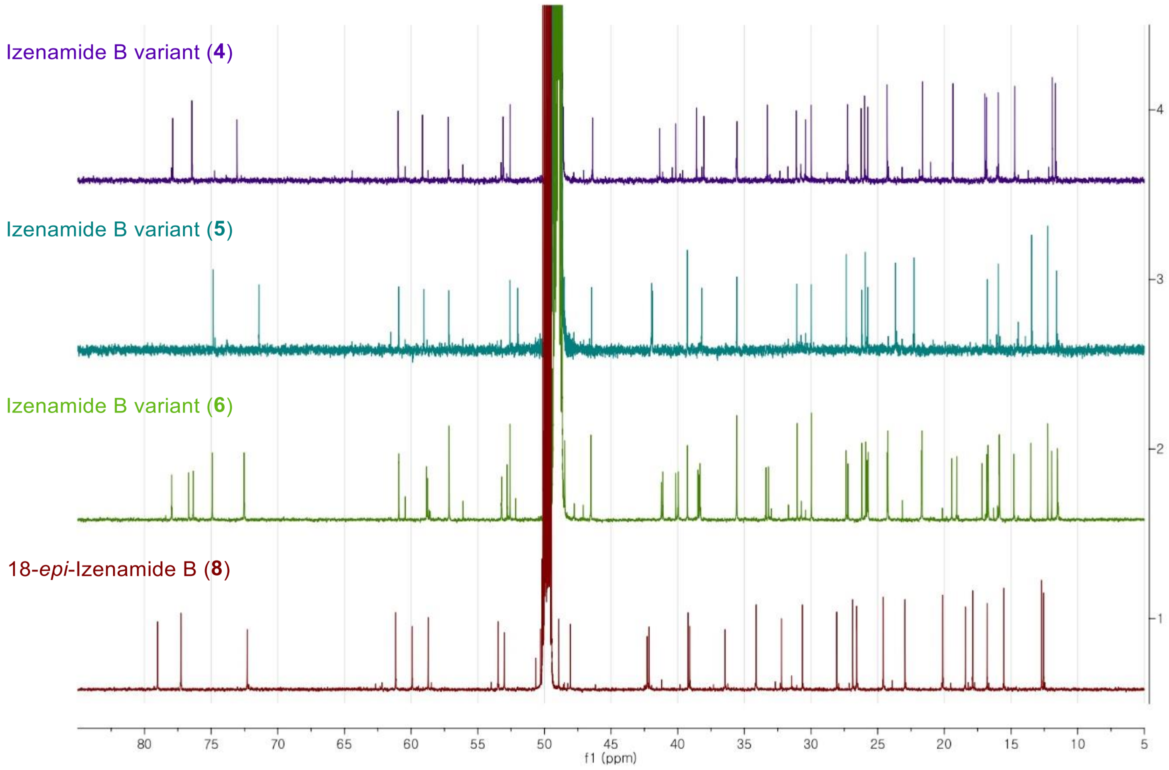

^{1}H and ^{13}C NMR of Izenamide B (**2**) (ref. *Molecules* **2019**, *24*, 3242)

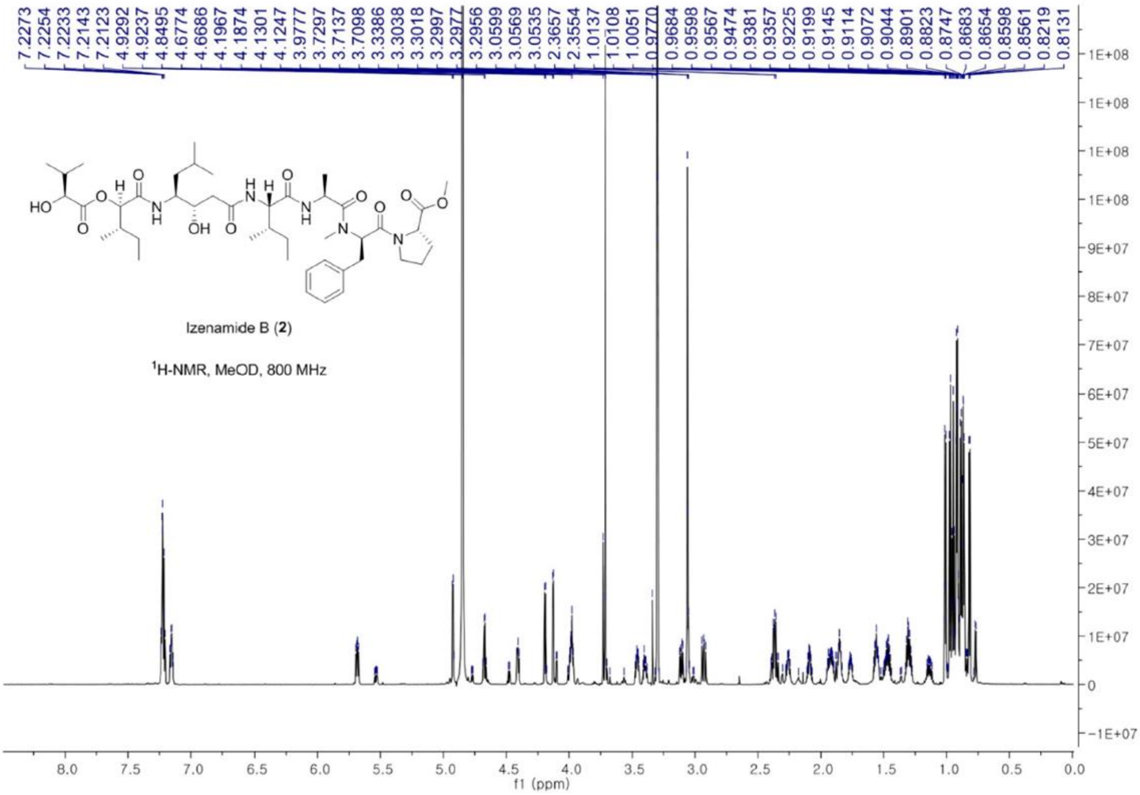

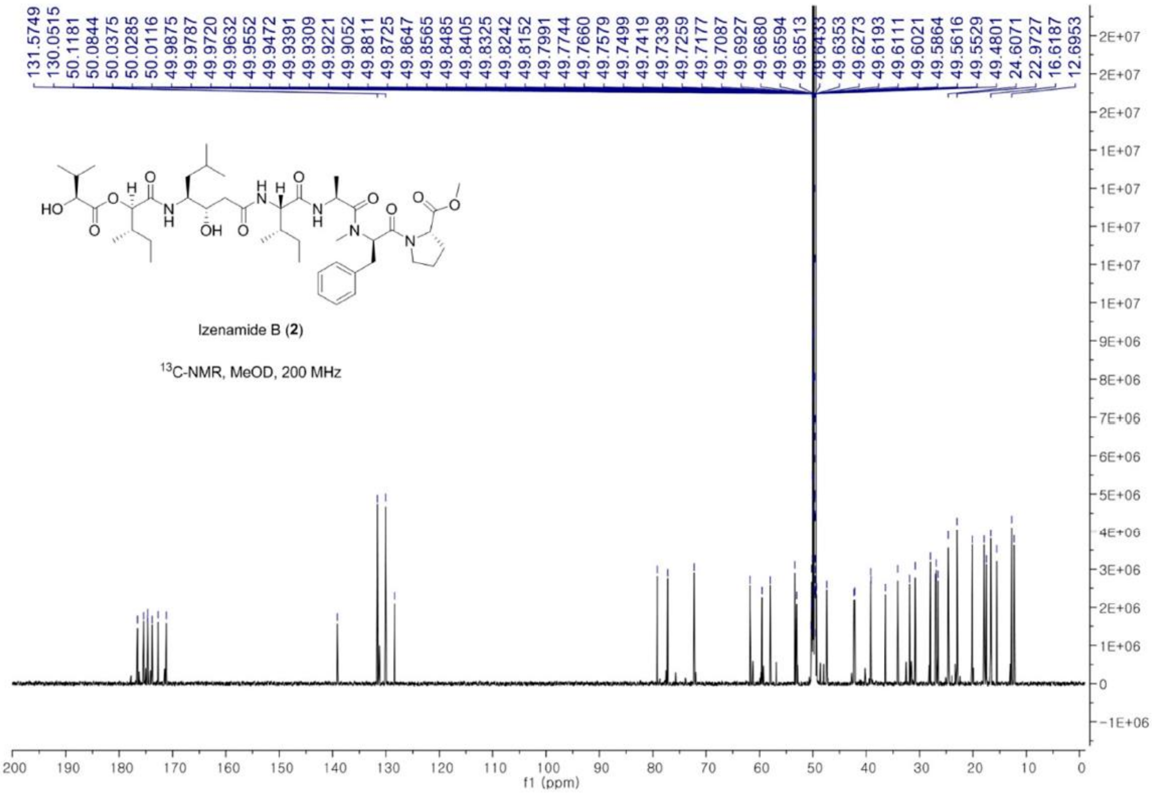

IV. HRMS spectra of Izenamide Variants (4-8)

HRMS spectra of izenamide B variant (**4**)

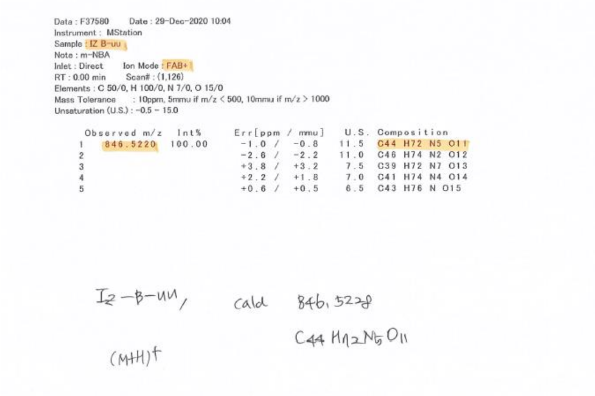

Data : F37580 Date : 29-Dec-2020 10:04
Instrument : MStation
Sample : IZ B-uu
Note : m-NBA
Inlet : Direct Ion Mode : FAB+
RT : 0.00 min Scan# : (1,126)
Elements : C 50/0, H 100/0, N 7/0, O 15/0
Mass Tolerance : 10ppm, 5mmu if m/z < 500, 10mmu if m/z > 1000
Unsaturation (U.S.) : -0.5 - 15.0

	Observed m/z	Int%	Err[ppm / mmu]	U.S.	Composition
1	846.5220	100.00	-1.0 / -0.8	11.5	C44 H72 N5 O11
2			-2.6 / -2.2	11.0	C46 H74 N2 O12
3			+3.8 / +3.2	7.5	C39 H72 N7 O13
4			+2.2 / +1.8	7.0	C41 H74 N4 O14
5			+0.6 / +0.5	6.5	C43 H76 N O15

Iz-B-uu, Cald 846.5228
$C_{44}H_{72}N_5O_{11}$
(M+H)+

HRMS spectra of izenamide B variant (**5**)

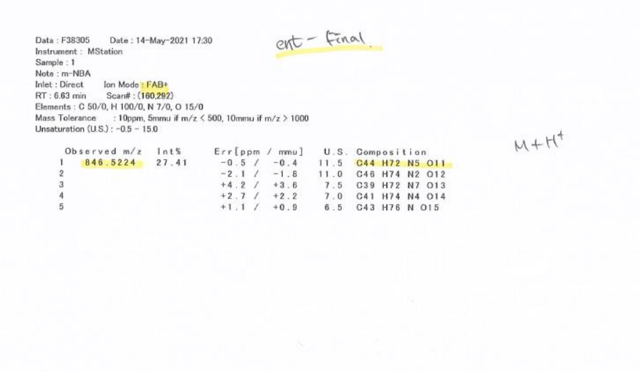

Data : F38305 Date : 14-May-2021 17:30
ent - Final
Instrument : MStation
Sample : 1
Note : m-NBA
Inlet : Direct Ion Mode : FAB+
RT : 6.63 min Scan# : (160,292)
Elements : C 50/0, H 100/0, N 7/0, O 15/0
Mass Tolerance : 10ppm, 5mmu if m/z < 500, 10mmu if m/z > 1000
Unsaturation (U.S.) : -0.5 - 15.0

M+H+

	Observed m/z	Int%	Err[ppm / mmu]	U.S.	Composition
1	846.5224	27.41	-0.5 / -0.4	11.5	C44 H72 N5 O11
2			-2.1 / -1.8	11.0	C46 H74 N2 O12
3			+4.2 / +3.6	7.5	C39 H72 N7 O13
4			+2.7 / +2.2	7.0	C41 H74 N4 O14
5			+1.1 / +0.9	6.5	C43 H76 N O15

HRMS spectra of izenamide B variant (**6**)

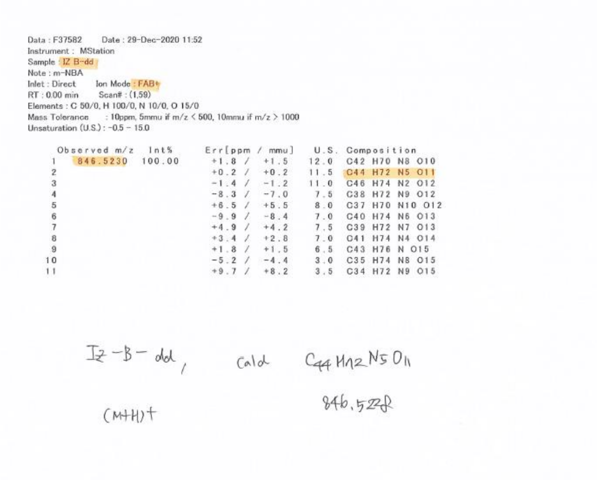

Data : F37582 Date : 29-Dec-2020 11:52
Instrument : MStation
Sample : IZ B-dd
Note : m-NBA
Inlet : Direct Ion Mode : FAB+
RT : 0.00 min Scan# : (1,59)
Elements : C 50/0, H 100/0, N 10/0, O 15/0
Mass Tolerance : 10ppm, 5mmu if m/z < 500, 10mmu if m/z > 1000
Unsaturation (U.S.) : -0.5 - 15.0

	Observed m/z	Int%	Err[ppm / mmu]	U.S.	Composition
1	846.5230	100.00	+1.8 / +1.5	12.0	C42 H70 N8 O10
2			+0.2 / +0.2	11.5	C44 H72 N5 O11
3			-1.4 / -1.2	11.0	C46 H74 N2 O12
4			-8.3 / -7.0	7.5	C38 H72 N9 O12
5			+6.5 / +5.5	8.0	C37 H70 N10 O12
6			-9.9 / -8.4	7.0	C40 H74 N6 O13
7			+4.9 / +4.2	7.5	C39 H72 N7 O13
8			+3.4 / +2.8	7.0	C41 H74 N4 O14
9			+1.8 / +1.5	6.5	C43 H76 N O15
10			-5.2 / -4.4	3.0	C35 H74 N8 O15
11			+9.7 / +8.2	3.5	C34 H72 N9 O15

Iz-B-dd, Cald $C_{44}H_{72}N_5O_{11}$
846.5228
(M+H)+

HRMS spectra of Sta-incoporated izenamide C (**7**)

Qualitative Compound Report

**Data File**	SIZ-04_8.d	**Sample Name**	SIZ-04
**Sample Type**	Sample	**Position**	P1-F1
**Instrument Name**	Instrument 1	**User Name**	
**Acq Method**	DIP method-2.m	**Acquired Time**	7/14/2019 3:52:56 PM
**IRM Calibration Status**	Success	**DA Method**	Default.m
**Comment**			
**Sample Group**		**Info.**	
**Stream Name**	LC 1		

Compound Table

Compound Label	RT	Mass	Abund	Formula	Tgt Mass	Diff (ppm)
Cpd 1: C43H69N5O11	0.21	831.5005	54036	C43H69N5O11	831.4994	1.32

Compound Label	RT	Algorithm	Mass
Cpd 1: C43H69N5O11	0.21	Find By Formula	831.5005

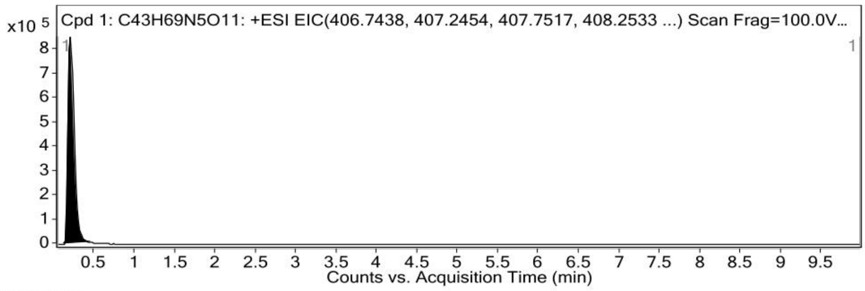

MS Spectrum

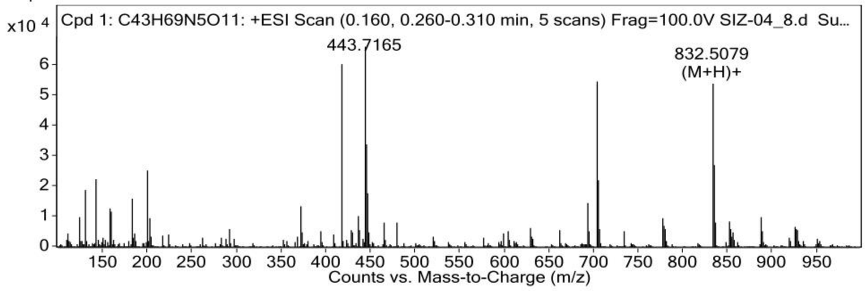

Qualitative Compound Report

MS Zoomed Spectrum

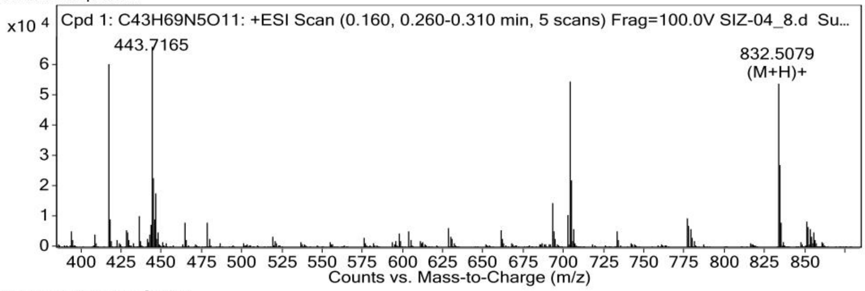

MS Spectrum Peak List

m/z	*Calc m/z*	Diff(ppm)	z	Abund	Formula	Ion
415.7504	415.7491	2.96	2	116	C43 H69 N5 O11	M+2
416.7581	416.757	2.79	2	60427	C43 H71 N5 O11	(M+2H)+2
417.2587	417.2585	0.45	2	32100	C43 H71 N5 O11	(M+2H)+2
443.7165				66157		
444.2182				34158		
444.7185				22898		
446.2639				17948		
832.5079	832.5066	1.5	1	54036	C43 H70 N5 O11	(M+H)+
833.5106	833.5098	1	1	27134	C43 H70 N5 O11	(M+H)+
854.4874	854.4886	-1.4	1	4910	C43 H69 N5 Na O11	(M+Na)+

--- End Of Report ---

HRMS spectra of 18-*epi*-izenamide B (**8**)

Qualitative Compound Report

**Data File**	SIZ-05_2.d	**Sample Name**	SIZ-05
**Sample Type**	Sample	**Position**	P1-F2
**Instrument Name**	Instrument 1	**User Name**	
**Acq Method**	DIP method-0p-1.m	**Acquired Time**	7/13/2019 4:57:47 PM
**IRM Calibration Status**	Success	**DA Method**	Default.m
**Comment**			
**Sample Group**		**Info.**	
**Stream Name**	LC 1		

Compound Table

Compound Label	RT	Mass	Abund	Formula	Tgt Mass	Diff (ppm)
Cpd 1: C44H71N5O11	0.209	845.516	17627	C44H71N5O11	845.515	1.21

Compound Label	RT	Algorithm	Mass
Cpd 1: C44H71N5O11	0.209	Find By Formula	845.516

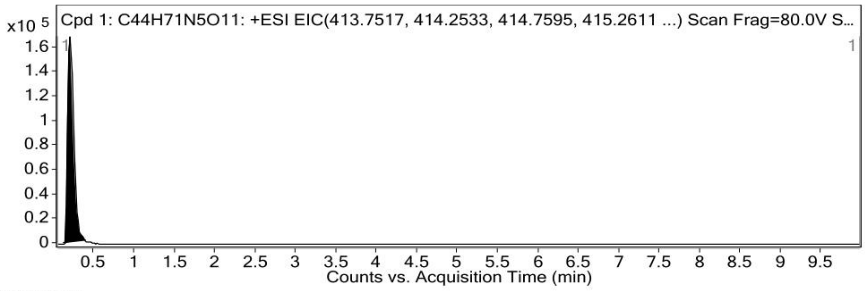

MS Spectrum

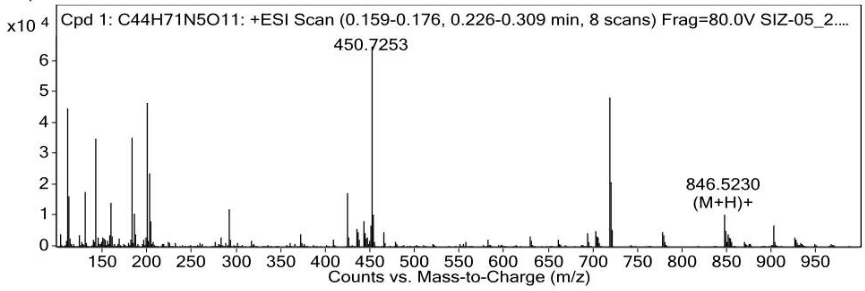

Qualitative Compound Report

MS Zoomed Spectrum

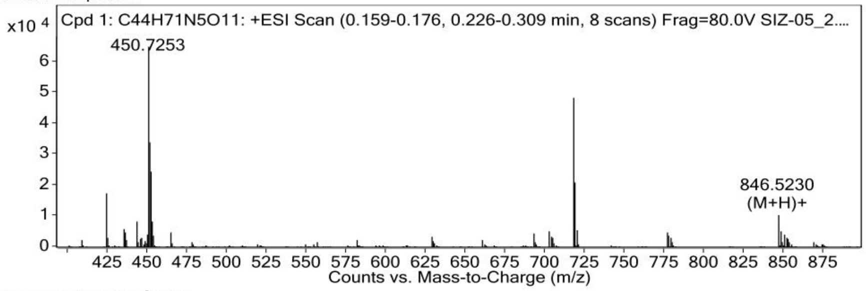

MS Spectrum Peak List

m/z	*Calc m/z*	Diff(ppm)	z	Abund	Formula	Ion
423.7658	423.7648	2.49	2	17627	C44 H73 N5 O11	(M+2H)+2
424.2676	424.2664	2.94	2	9040	C44 H73 N5 O11	(M+2H)+2
450.2268				6912		
450.7253				64820		
451.2268				34030		
451.7266				24693		
452.2283				10457		
452.7255				8556		
846.523	846.5223	0.87	1	10452	C44 H72 N5 O11	(M+H)+
868.5043	868.5042	0.03	1	1783	C44 H71 N5 Na O11	(M+Na)+

--- End Of Report ---
